# A reference collection of patient-derived cell line and xenograft models of proneural, classical and mesenchymal glioblastoma

**DOI:** 10.1038/s41598-019-41277-z

**Published:** 2019-03-20

**Authors:** Brett W. Stringer, Bryan W. Day, Rochelle C. J. D’Souza, Paul R. Jamieson, Kathleen S. Ensbey, Zara C. Bruce, Yi Chieh Lim, Kate Goasdoué, Carolin Offenhäuser, Seçkin Akgül, Suzanne Allan, Thomas Robertson, Peter Lucas, Gert Tollesson, Scott Campbell, Craig Winter, Hongdo Do, Alexander Dobrovic, Po-Ling Inglis, Rosalind L. Jeffree, Terrance G. Johns, Andrew W. Boyd

**Affiliations:** 1 QIMR Berghofer Medical Research Institute, Brisbane, Australia; 2 Royal Brisbane and Women’s Hospital, Brisbane, Australia; 3 Olivia Newton-John Cancer and Wellness Centre, Melbourne, Australia; 4 The University of Queensland, Brisbane, Australia; 5 Hudson Institute of Medical Research, Clayton, Victoria, Australia

**Keywords:** Cancer models, Cancer stem cells, CNS cancer, Cancer stem cells

## Abstract

Low-passage, serum-free cell lines cultured from patient tumour tissue are the gold-standard for preclinical studies and cellular investigations of glioblastoma (GBM) biology, yet entrenched, poorly-representative cell line models are still widely used, compromising the significance of much GBM research. We submit that greater adoption of these critical resources will be promoted by the provision of a suitably-sized, meaningfully-described reference collection along with appropriate tools for working with them. Consequently, we present a curated panel of 12 readily-usable, genetically-diverse, tumourigenic, patient-derived, low-passage, serum-free cell lines representing the spectrum of molecular subtypes of IDH-wildtype GBM along with their detailed phenotypic characterisation plus a bespoke set of lentiviral plasmids for bioluminescent/fluorescent labelling, gene expression and CRISPR/Cas9-mediated gene inactivation. The cell lines and all accompanying data are readily-accessible via a single website, Q-Cell (qimrberghofer.edu.au/q-cell/) and all plasmids are available from Addgene. These resources should prove valuable to investigators seeking readily-usable, well-characterised, clinically-relevant, gold-standard models of GBM.

## Introduction

Glioblastoma (GBM; WHO grade IV astrocytoma) is the most common, and most lethal, primary malignant adult brain cancer^1^. Despite surgery, radiotherapy and temozolomide chemotherapy, GBM patients have a median survival of <15 months and a 5-year survival rate of only 10%^2^. GBM almost always recurs following treatment and is then rapidly fatal with no current mainstay therapy effectively altering the course of the disease^1^. Two factors that contribute to treatment failure are the heterogeneity of GBM – in part represented by tumour cells with distinctly different patterns of gene expression^3,4^ (proneural, classical and mesenchymal GBM) – and the presence of glioma stem cells (GSCs), chemo-radiotherapy resistant cells that are able to reconstitute tumour architecture and are believed to be responsible for disease progression and recurrence^5–7^. Preclinical cell line and animal models that recapitulate these features accurately are essential if our understanding of GBM biology is to improve and more effective strategies to treat this almost uniformly fatal disease are to be developed.

Low-passage, serum-free, patient-derived GBM cell lines have proven to be the gold-standard in this regard. Methods to initiate and propagate such lines from patient tumour tissue are well established^8–10^. These approaches not only capture the genetic diversity of human GBM, but also help preserve the genomic fidelity of such cells and enrich for the presence of GSCs, enhancing their tumour-initiating capacity^11^. Significantly, xenograft tumours that arise from these cells are invasive and exhibit the histological hallmarks of high grade glioma, including hypercellularity, nuclear atypia and the presence of mitotic figures, with or without microvascular proliferation and (palisading) necrosis^12^. In essence, such cell lines are a highly relevant model for studying GBM, capturing the heterogeneity of this disease and recapitulating its cancer stem cell biology.

Despite their greater relevance for understanding human GBM, more widespread use of low-passage, serum-free, patient-derived cell lines in much GBM research appears not to be restricted by technical challenges but rather issues associated with the acquisition of patient tissue, unfamiliarity with existing lines, and/or much greater familiarity with alternative long-established (although poorly-representative) cell line models of GBM. Here we aspire to increase the wider use of low-passage, serum-free, patient-derived GBM cell lines amongst the cancer and cell biology research community by presenting a reference set - a curated, practical-size collection of genetically-diverse GBM cell lines established from different molecular subtypes of GBM - that form invasive xenograft tumours in immunodeficient mice. All lines have been characterised in significant practical detail, for interrogation and immediate use by researchers, with all data accessible via a user-friendly website. The availability of this data for *in silico* analysis should facilitate selection of lines suitable for hypothesis testing while the size, genetic diversity and subtype representation of the collection provides incentive for acquisition of the entire set to broadly and meaningfully inform studies of GBM.

In addition, we provide a set of tools to facilitate functional analysis of the lines. Lentiviral plasmids for bioluminescent and/or fluorescent labelling, expressing genes of interest and CRISPR/Cas9-targeted gene inactivation are presented. These further enhance the utility of the collection for investigating the biology of GBM.

## Results

### Establishment of a diverse set of low-passage, serum-free GBM cell lines from patient tumour tissue

Between November 2009 and June 2011, we established GBM cell lines as serum-free, adherent cultures from tissue collected from patients undergoing surgery at the Royal Brisbane and Women’s Hospital with a success rate of 75%. Twelve morphologically-diverse, rapidly-growing cell lines, that formed xenograft tumours when injected intracranially into NOD/SCID mice, were selected as a GBM reference collection for subsequent detailed characterisation. The patient cohort from which the cell lines were established had a median age of 70 years (range 48–84 years) and included 7 males and 5 females (Table 1). Eleven had primary GBM and one had recurrent GBM. Seven were right-sided tumours (frontal, parietal, temporal or occipital), five were left-sided (frontal or parietal). De-identified pathology reports for each tumour are attached as Supplementary Data S1. Overall survival ranged from 36 days to 7 years from the date of diagnosis of GBM – median survival was 5 months (Supplementary Fig. S1).

**Table 1:** Patient demographics.

Patient	Age (years)	Gender	Tumour type	Tumour site	Survival (days)
BAH1	75	Female	Primary GBM	Right frontal	94
FPW1	68	Male	Primary GBM	Right temporal	242
HW1	54	Female	Primary GBM	Right frontal parietal	89
JK2	75	Male	Primary GBM	Right frontal	178
MMK1	80	Female	Primary GBM	Right temporal	334
MN1	84	Female	Primary GBM	Left frontal	36
PB1	57	Male	Primary GBM	Left frontal	39
RKI1	57	Female	Primary GBM	Left temporal	Alive (7 years)
RN1	56	Male	Primary GBM	Left temporal	243
SB2b	48	Male	Recurrent GBM	Right parietal	420
SJH1	72	Male	Primary GBM	Left temporal	45
WK1	77	Male	Primary GBM	Right parietal occipital	121

Abbreviations: GBM, glioblastoma.

**Figure 1:**
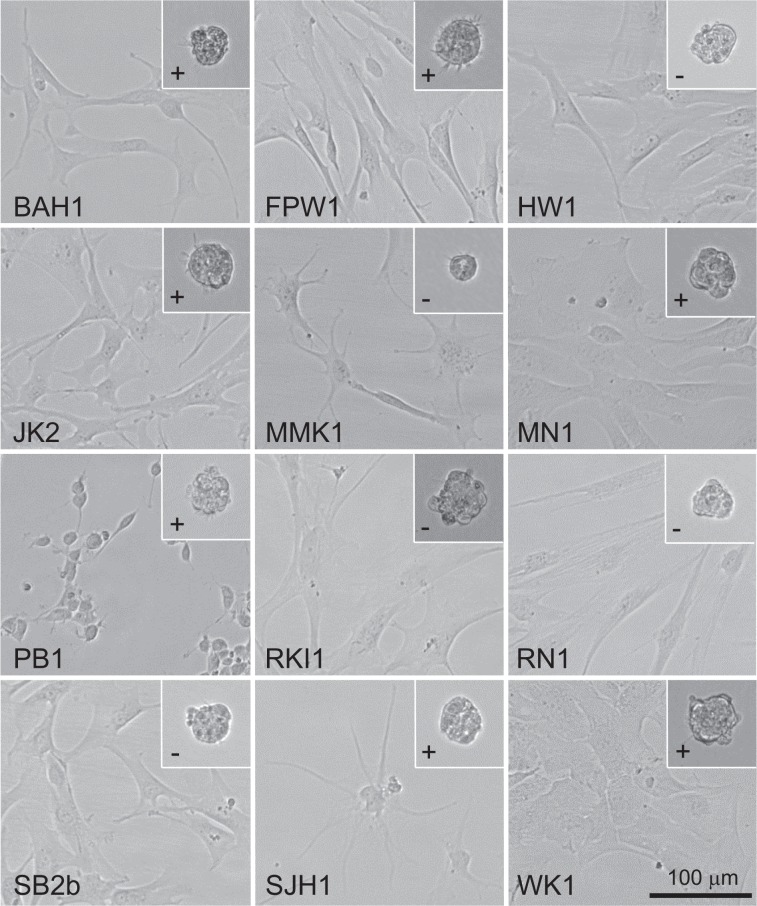
A diverse set of low-passage, serum-free, patient-derived GBM cell lines exhibiting a range of cell morphologies. Bright field images of GBM cell lines growing as adherent cultures on matrigel (main images) or as non-adherent tumourspheres (inset). Plus signs (+) indicate cell lines that readily form tumourspheres; minus signs (−) indicate cell lines that form tumourspheres poorly or not at all. Scale bar, 100 μm.

Low-passage, serum-free, patient-derived cell lines were established either from discrete pieces of tumour tissue or pooled tissue collected by an ultrasonic surgical aspirator^13^. Tumour tissue from both sources was mechanically and enzymatically dissociated and cultured on matrigel-coated plastic in serum-free RHB-A medium supplemented with epidermal growth factor and fibroblast growth factor 2. Medium was changed every three to four days and cells were washed with phosphate-buffered saline to remove tissue debris. Cells were passaged into larger flasks when nearing confluence and the first freezings were made from 75 cm^2^ flasks after continuous culture ranging from 14 to 30 days. The resultant cell lines exhibited a variety of cell morphologies (Fig. 1 and Supplementary Fig. S1). This heterogeneity was most marked among the different cell lines although also evident within many of them. Doubling times at passage 10 ranged between 38 and 109 hours (Table 2). Seven of the lines grew readily as tumourspheres when cultured without matrigel (Fig. 1); the other five lines formed tumourspheres poorly or not at all. This behaviour did not seem to correlate with GBM subtype.

**Table 2:** GBM cell line phenotypes.

Patient	Reference number	Doubling time (hours)	IDH1 status	MGMT methylation	Xenograft median survival (days)
BAH1	QIMR-B001	79.5 +/− 3.3	WT	Methylated	210 +/− 8
FPW1	QIMR-B002	48.1 +/− 4.7	WT	Unmethylated	196 +/− 4
HW1	QIMR-B003	55.8 +/− 2.7	WT	Methylated	174 +/− 14
JK2	QIMR-B004	94.2 +/− 6.0	WT	Unmethylated	147 +/− 9
MMK1	QIMR-B005	52.4 +/− 4.5	WT	Unmethylated	157 +/− 15
MN1	QIMR-B006	44.9 +/− 1.0	WT	Unmethylated	258 +/− 20
PB1	QIMR-B007	79.4 +/− 6.3	WT	Unmethylated	71 +/− 1
RKI1	QIMR-B008	72.9 +/− 5.3	WT	Methylated*	248 +/− 6
RN1	QIMR-B009	37.5 +/− 1.9	WT	Unmethylated	81 +/− 2
SB2b	QIMR-B010	108.7 +/− 6.9	WT	Methylated	120 +/− 4
SJH1	QIMR-B011	67.3 +/− 4.7	WT	Unmethylated	148 +/− 2
WK1	QIMR-B012	46.2 +/− 1.0	WT	Unmethylated	150 +/− 2

Abbreviations: *homozygous SNP rs16906252; IDH1, isocitrate dehydrogenase 1; MGMT, methyl guanine methyltransferase; WT, wild type.

Expression of O-6-methylguanine-DNA methyltransferase (MGMT) from the *MGMT* gene confers resistance in GBM cells to the alkylating chemotherapy agent temozolomide. Methylation of the *MGMT* promoter silences MGMT transcription. The *MGMT* promoter was found to be methylated in four of the GBM cell lines, one of which contained a homozygous single nucleotide polymorphism (rs16906252) (Table 2).

Mutation of the gene encoding isocitrate dehydrogenase (NADP(+)) 1, (*IDH1*) at arginine 132 or *IDH2* at arginine 172 is associated with secondary GBM, GBM that has arisen from lower grade glioma. All lines were found to be wild type for *IDH1*and *IDH2* so can be regarded as glioblastoma, IDH-wildtype.

Each cell line was short tandem repeat profiled (Supplementary Table S1). All STR profiles were unique and alleles for the AMEL locus matched the gender of the patients from which each cell line was established. There was no evidence of significant relatedness with known cell lines or evidence of cross-contamination among the 12 lines. All lines tested mycoplasma-free.

To complement the collection of GBM cell lines, we created a set of lentiviral plasmids for ectopic gene expression or CRISPR/Cas9-mediated gene inactivation (Supplementary Table S2). Expression plasmids contain the chimaeric human CMV enhancer/chicken beta-actin promoter for strong gene expression in GBM cells and confer resistance to puromycin, hygromycin B or G418. By default, they contain the firefly luciferase gene to generate bioluminescent cell lines. Lentiviral CRISPR/Cas9 plasmids were derived from lentiCRISPRv2 (Supplementary Fig. S2) and confer resistance to puromycin, hygromycin B, G418 or blasticidin S. To aid the establishment of transformed cell lines with these vectors we determined the sensitivity of each of the GBM cell lines to these antibiotics (Table 3).

**Table 3:** GBM cell line antibiotic sensitivity (μg/ml).

GBM cell line	Puromycin	Hygromycin B	G418	Blasticidin S
BAH1	0.5 (5)	100	200	1
FPW1	0.5	200 (9)	300 (9)	4
HW1	0.4	200	300	2
JK2	0.4	100	300	2
MMK1	0.5	100	200	2
MN1	0.4 (5)	200	200	2
PB1	0.2 (5)	200	200	1
RKI1	0.5	200 (10)	200 (10)	4
RN1	0.5	100	200	1
SB2b	0.5 (5)	200	400	1
SJH1	0.5	200	300	1
WK1	0.5	200 (9)	400	4

Notes: 7 days unless specified ().

### Characterisation of GBM cell line intracranial tumourigenicity in NOD/SCID mice

**Figure 2:**
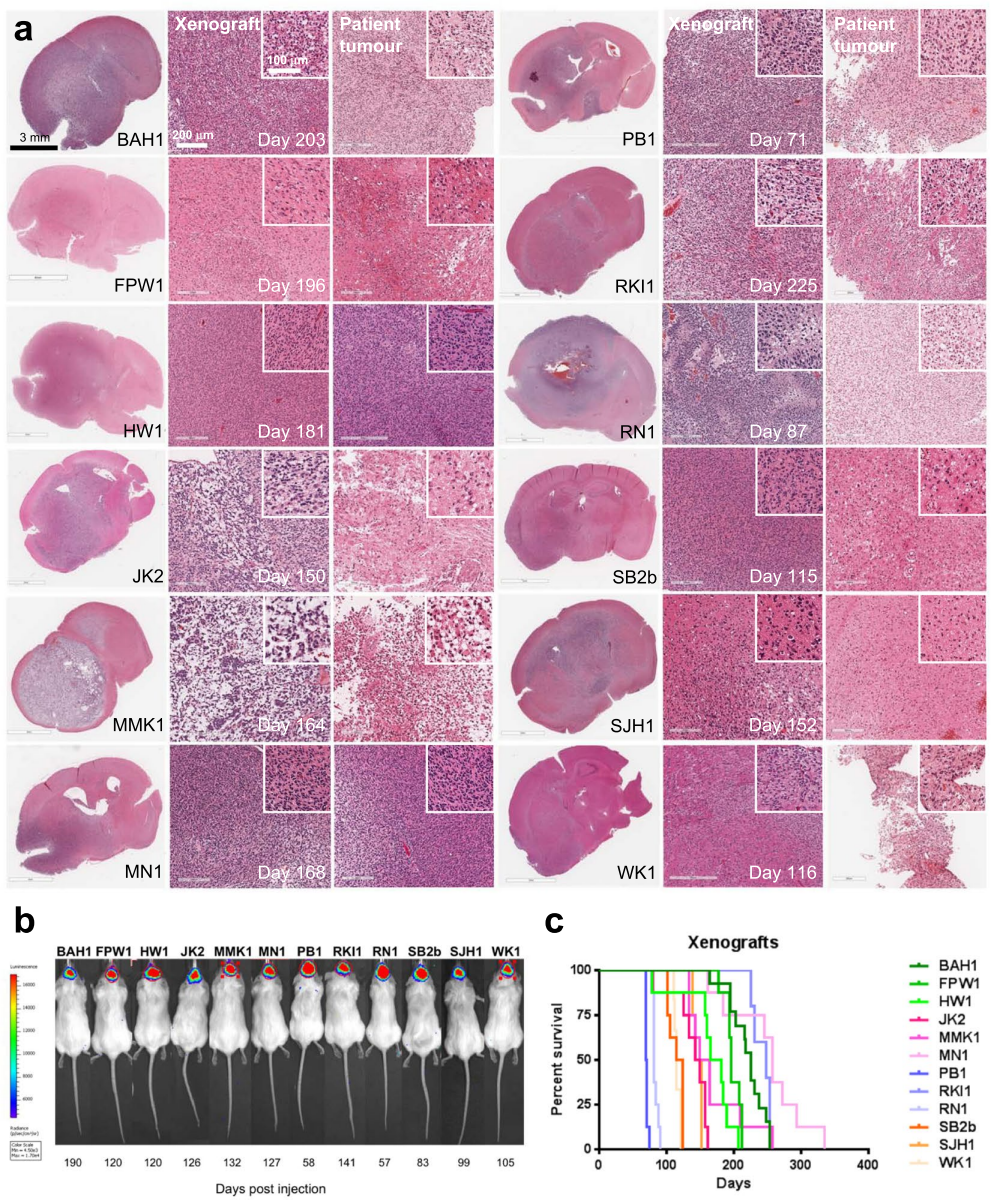
Bioluminescent xenograft tumour formation in NOD/SCID mice. **(a)** Comparative histology of xenograft tumours formed by the GBM cell lines and the patient tumours they were established from. First column, haematoxylin and eosin (H&E)-stained, coronal sections of mouse brains showing invasive xenograft tumours. Scale bar, 3 mm. Comparative H&E-stained sections of matched xenograft (middle column) and patient tumours (right column). Scale bar 200 μm, inset 100 μm. **(b)** Bioluminescent intracranial xenograft tumours produced by the GBM cell lines expressing firefly luciferase. Stably-transduced lines were created by transduction with lentivirus containing pF CAG luc puro and selection with puromycin. Time to develop the bioluminescent xenograft tumours detected is shown below each mouse. **(c)** Kaplan-Meier survival curves for NOD/SCID mice harbouring xenografts initiated by intracranial injection of each of the GBM cell lines.

To investigate the tumourigenicity of each cell line, 1.5 × 10^5^ cells were injected intracranially into the right striatum of cohorts of 5-week old NOD/SCID mice. All cell lines formed invasive tumours with similar histology to the patient tumours from which they were established (Fig. 2a), including evidence of focal necrosis and vascular proliferation. No clear correlation though was apparent between xenograft histology and median survival time.

To enable tumour growth to be monitored by bioluminescence, each cell line was also transduced with a lentiviral transfer plasmid expressing luciferase from a chimaeric CMV enhancer/chicken beta actin promoter. Luciferase-expressing cells generated bioluminescent xenograft tumours with tumour-bearing mice having median survival times ranging from 2.5–9 months (Table 2 and Fig. 2b,c). Luciferase expression was not observed to alter xenograft median survival.

### Genomic characterisation of the GBM cell lines

**Figure 3:**
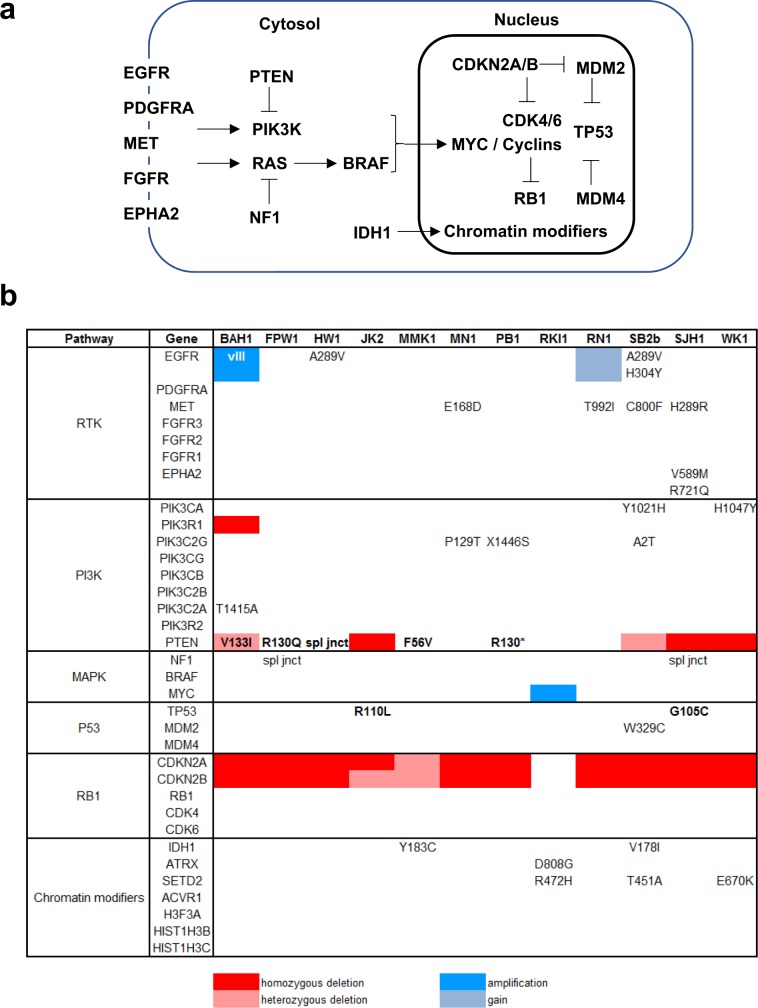
Genomic profiling of the GBM cell lines. **(a)** Pathways most frequently affected by genomic changes in GBM showing the relationships of the genes involved. **(b)** Single nucleotide variants (SNVs), intragenic deletions and gene copy number changes in genes in these pathways detected in the GBM cell lines. SNVs shown in bold are homozygous, otherwise heterozygous. Blanks indicate wild type genes.

To catalogue the genotype of each GBM cell line, we performed exome sequencing. Genomic alterations characteristic of GBM were common among the 12 lines (Fig. 3a,b). Most frequent was homozygous deletion of the tumour suppressor *CDKN2A*, exhibited by 10 (83%) of the lines. Homozygous deletion of *PTEN* was detected in three lines and homozygous single nucleotide variants (SNVs) that were predicted to be pathogenic^14^ were present in five others. *TP53* nonsynonymous SNVs were infrequent, with a homozygous predicted pathogenic missense SNV (G105C) in SJH1 and a homozygous neutral missense SNV (R110L) in JK2. At least half of the lines contained potential pathogenic changes in GBM-associated receptor tyrosine kinase (RTK) genes. Amplification of the oncogenic vIII variant of *EGFR* (EGFRvIII) was present in one of the lines (BAH1) and copy number gain of wild type *EGFR* was detected in another (RN1). Two further lines (HW1 and SB2b) contained pathogenic SNVs in *EGFR* that have only been reported previously in GBM^15^. Missense SNVs also were detected in *MET* and *EPHA2*, including a substitution in the latter that creates a variant (EphA2 R721Q) with altered signalling. Amplification of *MYC*, detected in only 1.5% of GBM^16,17^, was present in the cell line established from a long-term survivor (RKI1). Nonsynonymous single nucleotide variant calls for each of the cell lines, their zygosity and predicted measures of their pathogenicity are listed as Supplementary Data S2.

### Gene expression and molecular subtype analysis of matched patient tumours, cell lines and xenografts

**Figure 4:**
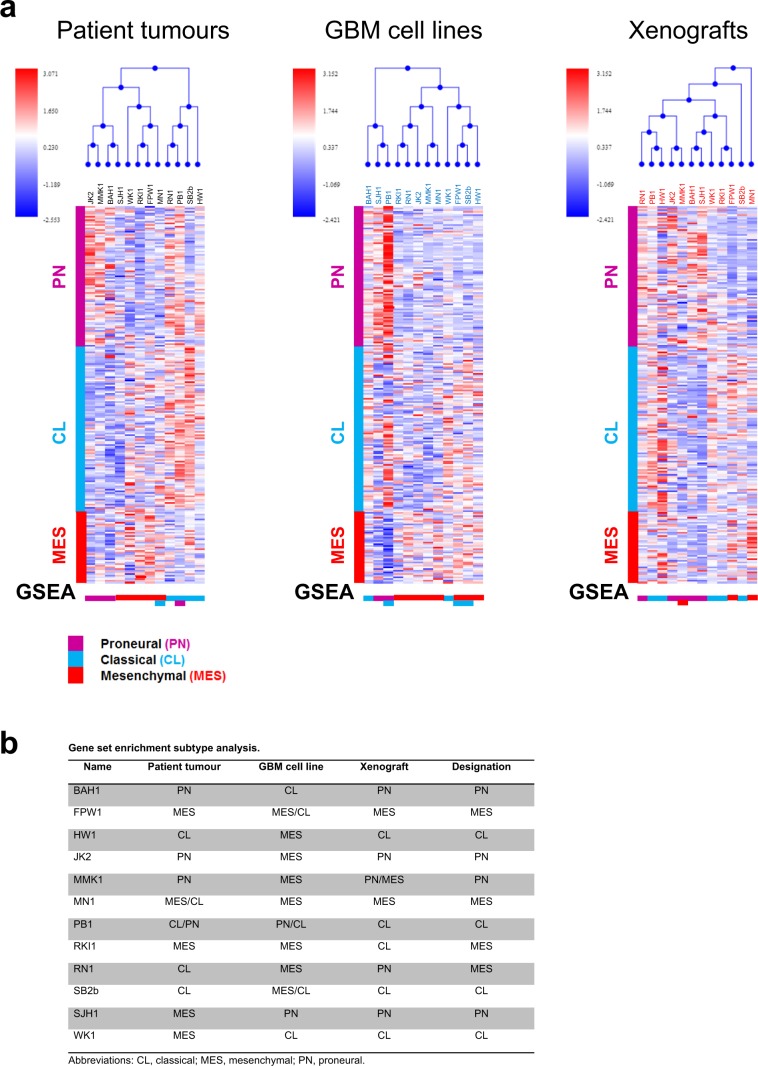
Molecular subtyping of matched patient tumours, GBM cell lines and xenografts. **(a)** Hierarchical cluster analysis (top), corresponding heatmaps (middle) and gene set enrichment analysis (GSEA) (bottom) of patient tumours, derived GBM cell lines and matched murine xenografts initiated by the cell lines identifying gene expression patterns characteristic of proneural, classical and mesenchymal GBM. **(b)** Summary of GSEA results.

Although GBM is a genetically and phenotypically heterogeneous cancer, distinct molecular subtypes can be distinguished, including on the basis of gene expression. The most widely adopted molecular classification system uses an 840-gene signature to distinguish proneural, neural, classical and mesenchymal GBM^4^. These subtypes resemble distinct normal neural cell types, have different prognoses, respond differently to treatment and are associated with characteristic genetic alterations^4^, thus these distinctions appear to have patho-biological significance. Since the introduction of this classification schema by The Cancer Genome Atlas consortium, reservations have been expressed concerning the identity of the neural GBM subtype. Greatest support in the literature is found for the proneural, classical and mesenchymal subtypes^18^, with the suggestion that neural GBM likely represents normal brain tissue containing a small percentage of tumour cells^19^, an observation that could account for this subtype lacking characteristic genetic alterations^20^. To molecularly subtype the tumour tissue from which our GBM cell lines were derived, we measured RNA expression and performed hierarchical cluster analysis (Fig. 4a) and gene set enrichment analysis (GSEA) (Supplementary Fig. S3) using the classifier gene sets for proneural, classical and mesenchymal GBM. This showed our GBM cell lines were established from tumours of each of these molecular subtypes of GBM. Consistent with the finding that individual tumours may contain subpopulations of tumour cells of different molecular subtypes^21,22^, and reflecting the heterogeneity of this disease, several of the tumour specimens exhibited gene expression profiles of more than one GBM subtype (Supplementary Fig. S3). Inclusion of the neural signature in the GSEA analysis showed several of the specimens (BAH1, HW1, RKI1 and SJH1) also exhibited significant expression of neural genes, consistent with the sample containing both tumour and normal tissue and this was confirmed by histological examination of the tumour tissue adjacent to that used to extract RNA (data not shown).

To investigate whether the GBM cell lines retained the phenotype of the tumour from which they were derived, we similarly analysed both cultured cells and the xenografts they produced (Fig. 4a and Supplementary Fig. S3). We found the majority of GBM cell lines (7/12) exhibited a mesenchymal gene expression signature. When transplanted into NOD/SCID mice, however, most cell lines (7/12) produced xenografts of the same molecular subtype as the tumour from which they arose, including five that exhibited a different molecular subtype when grown in culture. Cell lines derived from mesenchymal patient tumours most often (4/5) gave rise to xenografts of a different subtype (most often classical GBM) despite retaining their mesenchymal subtype in culture. Notably, in only two cases was the same molecular subtype observed for patient tumour, GBM cell line and xenograft: MN1 (mesenchymal) and SB2b (classical).

Our final designation of the subtype of each GBM cell line and a summary of the results for patient tumours, cell lines and xenografts is shown in Fig. 4b. Most designations match the subtype of the primary tumour. In the three that did not (RN1, SJH1 and WK1), the significantly higher gene set enrichment scores for the cell lines and/or xenografts led us to designate these as mesenchymal, proneural and classical respectively.

We also noted MGMT expression in the GBM cell lines was consistent with MGMT promoter methylation status. Interestingly, although the MGMT promoter was unmethylated in the MN1 cell line, no MGMT expression was detected. As exome sequencing showed there were no alterations in the MGMT gene, this indicated factors besides promoter methylation also determine MGMT expression.

As a reference resource, microarray gene expression data for all patient tumours, GBM cell lines and xenografts are provided as Supplementary Data S3. RNAseq analysis of gene expression for the GBM cell lines also is provided as Supplementary Data S4. Microarray raw data have been deposited with the GEO repository with the series accession number GSE118793 and RNAseq raw data is available for download from the NCBI sequence read archive with SRA accession number PRJNA508446.

### GBM-associated protein expression

**Figure 5:**
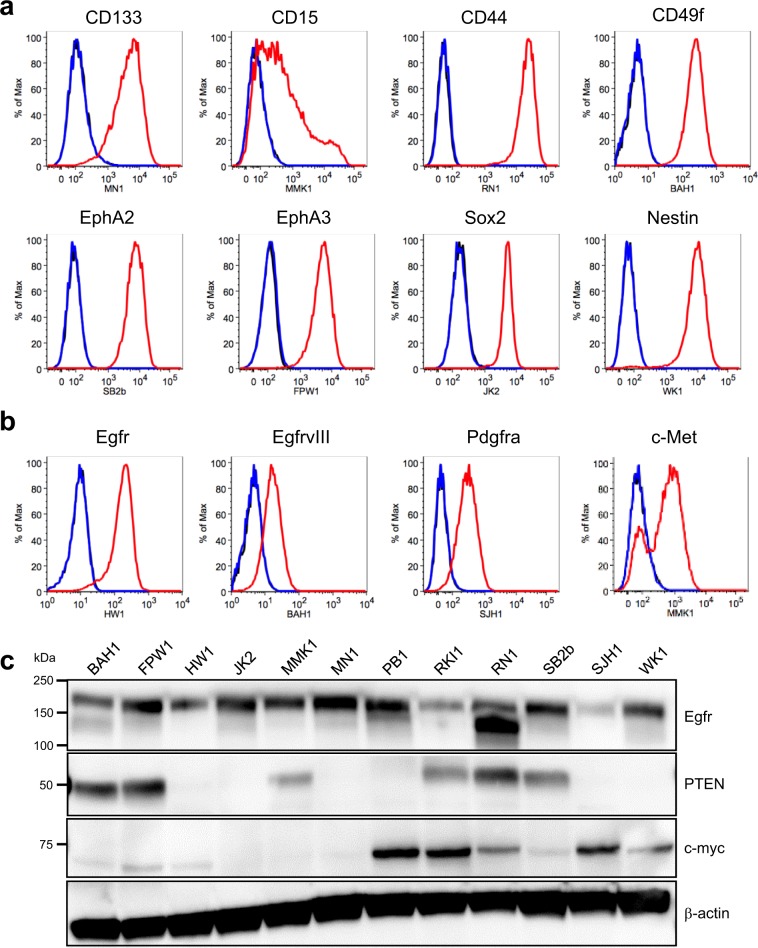
GBM-associated protein expression in the GBM cell lines. Representative flow cytometry analysis of **(a)** stem cell markers and **(b)** selected receptor tyrosine kinases in the GBM cell lines cultured as adherent monolayers on matrigel. (**c**) Western blot analysis of Egfr, Pten and c-myc expression in the GBM cell lines. kDa, kilodalton.

Several cellular proteins are used as markers of glioblastoma stem and progenitor cells (GSCs) and are employed to isolate these cells by flow cytometry or affinity capture. We therefore investigated the expression of GSC markers by flow cytometry in cultured GBM cell lines (Fig. 5a and Supplementary Data S5), including the GSC marker CD133, whose protein level is independent of CD133 mRNA expression^23^, CD15, CD44, CD49f, EphA2^24^ and EphA3^25^, and the progenitor cell markers Sox2 and Nestin^7^. All were detected among the 12 GBM cell lines, consistent with the culture conditions maintaining the presence of GSCs. Some markers were detected only in some cell lines, others in a subset of cells in a given line. We also investigated expression of several other GBM-associated RTKs, in addition to EphA2 and EphA3, including Egfr, EgfrvIII, Pdgfrα and c-Met (Fig. 5b and Supplementary Data S5). Our findings were in accord with our exome sequencing (EgfrvIII) and mRNA expression analysis, with widespread expression of Egfr and EphA2 and more restricted expression of the other RTKs. Widespread expression of Egfr also was detected by western blot (Fig. 5c and Supplementary Fig. S4); interestingly *EGFR* copy number gain in the RN1 cell line was associated with strong expression of a low molecular weight form of Egfr which did not appear to be EgfrvIII as this was not detected by flow cytometry using an EgfrvIII-specific monoclonal antibody (Supplementary Data S5). Similarly, western blot (Fig. 5c and Supplementary Fig. S4) confirmed lack of expression of PTEN in lines in which *PTEN* was homozygously deleted (JK2, SJH1 and WK1), contained a splice junction mutation (HW1) and showed gain of a premature STOP codon (PB1, R130*). PTEN expression also was not detected in a line (MN1) in which no mutations were detected in the coding sequence or splice junctions of the PTEN gene by exome sequencing. Increased expression of c-myc in the cell line (RKI1) with *MYC* amplification also was confirmed by western blot (Fig. 5c and Supplementary Fig. S4). Interestingly, several other lines (PB1, SJH1, RN1 and WK1) also showed evidence of increased c-myc expression, suggesting they may be c-myc-driven GBMs. Protein expression (or lack thereof) from other GBM-associated genes, including *CDKN2A*, *CDKN2B*, *FGFR1*, *NF1*, *TP53*, *RB1* and *IDH1* (Fig. 3b), was confirmed by proteomic analysis (Supplementary Data S6).

## Discussion

Many preclinical and laboratory investigations of human GBM use cell lines. Historically these have been maintained in serum-supplemented medium that, while providing growth factors and other mitogens, introduces genotoxic stress. This, coupled with extensive passaging, produces cell line and xenograft models whose phenotypes diverge significantly from their tumour of origin. Low passage, patient-derived GBM cell lines that are maintained in serum-free medium supplemented with EGF and FGF2, have more recently been adopted by many investigators to circumvent these pressures and produce models that more accurately recapitulate the cardinal features of GBM^11^. Despite their clear benefits and ease of use, the wider adoption of such cell lines is often restrained by several factors. Initiating the necessary collaborations, applying for human research ethics approval and establishing cell lines from patient tumour tissue requires the investment of significant time and effort. Alternatively, sourcing such lines from other laboratories often comes with limited understanding regarding the origin and phenotype of the cells received as well as a lack of familiarity with working with them. Having experienced both scenarios, we have tried to increase investigator access to, and confidence with using, such cell lines by compiling a meaningfully-sized reference collection of extensively-characterised, low-passage, serum-free, patient-derived GBM cell lines, along with bespoke tools for working with them, which we hope will lead to more widespread use of this more relevant resource for investigating human GBM. While a number of laboratories have reported GBM cell line and mouse xenograft models of GBM, and a larger publicly-available, partially-phenotyped, GBM cell line collection exists^26^, we believe the practical size of our collection, coupled with the extent and practical-focus of its genotypic and phenotypic characterisation, meets a need in the GBM research community for a reference collection suitable for acquiring *en bloc* for laboratories yet to make the switch to using such cell lines or alternatively as a reliable source of individual lines with defined phenotypes and genotypes from which other laboratories can supplement their existing resources.

During the preparation of this manuscript, a revised TCGA classification system was reported for IDH-wild type GBM^27^. This retains the proneural, classical and mesenchymal nomenclature (and drops the neural subtype) and uses a smaller number of genes (50 for each subtype) to subtype GBM. When this smaller classifying gene set was used to subtype our tumour samples (all IDH-wild type), all were classified with much lower (and often nonsignificant) normalised enrichment scores. In light of this experience, there may be advantages in using the original TCGA classification system, with its larger number of classifier genes, to distinguish proneural, classical and mesenchymal GBM, with the inclusion of the neural classifier to identify samples that contain few tumour cells.

The key finding of our subtype analysis of matched patient tumours, GBM cells lines and xenografts was that low-passage, serum-free cell lines most often gave rise to xenografts of the same molecular subtype as the patient tumours from which they were derived, although frequently exhibited a different GBM subtype during *in vitro* culture. That most of our GBM cell lines exhibited a mesenchymal gene expression signature, regardless of the subtype of their tumour of origin, was striking yet is an inconsistent finding in the literature^4,26,28^. That serum-free cell lines derived from mesenchymal patient tumours often produced xenografts of a different subtype, despite retaining their mesenchymal subtype in culture, has been observed independently^26,28^. Yet to be determined is whether the apparent subtype switching is the result of the emergence of pre-existing subpopulations of cells^29^ or represents inherent plasticity^28^. While our patient tumours may contain GBM cells of more than one molecular subtype, which could account for the subtype “switch” observed between patient tumours and the derived GBM cell lines (*in vitro* culture potentially selecting for one subtype over another), subtype differences between the cell lines and the xenografts they produce suggest some GBM cells may be plastic and that their phenotype can change in response to their environment. We anticipate that the heterogeneity inherent in this collection of low-passage, serum-free GBM cell lines will be helpful for resolving this and other issues of GBM cell biology.

Interestingly, only one EgfrvIII-expressing and one Egfr-amplified cell line was established despite *EGFR* alterations occurring in over half of GBM tumours^20^. This is a common observation and may be related to a requirement *in vivo* that is lost in culture. As we observed that omission of recombinant Egf from the culture medium increased expression of EgfrvIII in the BAH1 cell line (data not shown), it may be worthwhile to routinely establish GBM cell lines both in the presence and absence of Egf in the culture medium in order to attempt to increase the frequency at which Egfr/EgfrvIII-amplified cell lines are established.

Investigations to characterise our GBM cell lines were performed between passage 8 and 15, at which time we considered them “established”. Each line has been passaged at least 30 to 50 times now without notable change in tumourigenicity, growth rate, morphology or antibiotic sensitivity. While proneural tumours are typically less aggressive, our proneural xenograft-forming cell lines did not have the longest median xenograft survival (166  +/− 9 days for proneural xenografts versus 162 +/− 8 days for classical and mesenchymal combined) suggesting this behaviour may not apply to xenograft models. However, our sample size is small and larger numbers will be required to confirm this.

In our hands, as well as in other laboratories, the GBM cell lines have proven useful for a variety of purposes. These include the identification and validation of GBM- and GSC-associated therapeutic targets^25,30–32^, investigations of GBM metabolism^33^, microRNA function in GBM^34^, GBM sensitivity to ionising radiation^35^, PET/MRI imaging of GBM^36,37^, GBM cell response to environmental stiffness^38^ and the evaluation of novel agents for treating GBM^39–45^. In light of this demonstrated utility, we anticipate this GBM cell line reference collection, and accompanying set of lentiviral plasmids, will both prove to be valuable resources for the scientific research community.

## Methods

### Tumour tissue and cell culture

Patient tumour tissue was collected from patients undergoing surgery at the Royal Brisbane and Women’s Hospital (RBWH), with informed consent and human ethics approval from the QIMR Berghofer Medical Research Institute and RBWH human research ethics committees. All human studies have been performed in accordance with the ethical standards laid down in the 2013 version of the 1964 Declaration of Helsinki. Tumour tissue was examined by a neuropathologist (TR) to determine tumour type and grade. Patient-derived cell lines were established as reported previously^13^. These were cultured as adherent monolayers in matrigel (BD Biosciences)-coated vessels using RHB-A stem cell culture medium (StemCells Inc) supplemented with 20 ng/ml EGF (Gibco) and 10 ng/ml FGFb (Gibco) and containing 100 U/ml penicillin and 100 μg/ml streptomycin, or as tumourspheres using StemPro NSC SFM (Invitrogen). Cells were cultured in 5% CO_2_/95% humidified air at 37 °C. All cell lines were routinely tested for mycoplasma and confirmed to be mycoplasma-free.

### Short tandem repeat profiling

Short tandem repeat (STR) profiling to establish the unique genomic identity of each cell line and exclude cross-contamination was performed by the QIMR Berghofer Medical Research Institute Analytical Facility. Alleles for 10 human loci (THO1, D21S11, D5S818, D13S317, D7S820, D16S539, CSFIPO, AMEL, vWA and TPOX) were assayed and compared with the ATCC STR database (https://www.atcc.org/STR_Database.aspx) using the standard match threshold of 80%.

### *MGMT* promoter methylation detection

MGMT promoter methylation status was determined by bisulphite pyrosequencing as described^46^.

### *IDH1* mutation status

The mutation status of Idh1 at R132 was determined by PCR amplification of *IDH1* from genomic DNA using the oligonucleotide primers IDH1 forward: CGGTCTTCAGAGAAGCCATT and IDH1 reverse: GCAAAATCACATTATTGCCAAC and direct Sanger sequencing of the resultant PCR product using the IDH1 forward primer. *IDH1* and *IDH2* mutation status also was confirmed by exome sequencing.

### Proliferation assays and doubling time

Cells were seeded at a density of 2 × 10^3^ cells per well in 100 μl of medium in matrigel-coated 96-well plates and MTS [3-(4,5-dimethylthiazol-2-yl)-5-(3-carboxymethoxyphenyl)-2-(4-sulfophenyl)-2H-tetrazolium] proliferation assays were performed using a CellTiter 96 AQ_ueous_ One Solution cell proliferation assay kit (Promega). Cells were incubated with 20 μl of MTS reagent and incubated for one hour at 37 °C. The ratio of absorbance in the test wells to control wells (containing culture medium and no cells) was calculated as relative MTS activity and used as a surrogate for the number of viable cells in culture. Average relative MTS activity from three replicate wells was plotted against time and the doubling time calculated using GraphPad Prism software v.6.0 (GraphPad software, LaJolla, CA, USA).

### Plasmid construction

Lentiviral expression plasmids were derived from pF_U_MCS_SV40Puro and pF_U_G147EV16_PGK_Hygro^47^ (a gift from John Silke). The chimaeric human CMV enhancer/chicken beta-actin promoter from pCAGIG^48^ (a gift from Connie Cepko) was subcloned in place of the ubiquitin promoter in both vectors and firefly luciferase was subcloned from pGL2 Basic (Promega) to generate pF CAG luc puro and in place of G147EV16 to produce pF CAG luc hygro. pF CAG luc IRES neo was created by cloning an IRES-neo cassette into pF CAG luc hygro in place of PGK-hygro. The IRES sequence was amplified by PCR from pIRES2-DsRed-Express (Clontech) and the neo sequence was amplified from pSuperior neo + GFP (OligoEngine). Genes of interest can be subcloned between the unique *Bam*HI and *Nhe*I restriction sites in place of luciferase. pF CAG BFP2 IRES neo was created by cloning PCR-amplified BFP2 from pBAD-mTagBFP2^49^ (a gift from Vladislav Verkhusha, Addgene plasmid #34632) between the *Bam*HI and *Nhe*I restriction sites of pF CAG luc IRES neo.

CRISPR/Cas9 plasmids were constructed from lentiCRISPRv2^50^ (a gift from Feng Zhang, Addgene plasmid #52961) and are designed to confer resistance to puromycin, hygromycin B, G418 or blasticidin S. These were created by first destroying the *Mlu*I restriction site of lentiCRISPRv2 at nucleotide position 231 by *Mlu*I digestion followed by Klenow end-filling and re-ligation to create lentiCRISPRv2 puro, then cloning P2A-antibiotic resistance cassettes between the *Mlu*I and *Bam*HI restriction sites of lentiCRISPRv2 puro. The P2A sequence was from lentiCRISPRv2, the hygromycin resistance gene cassette from pF CAG luc hygro, the neomycin resistance gene cassette from pF CAG luc IRES neo, and the blasticidin resistance gene cassette from pcDNA6TR (Invitrogen). Gene fusions were created by overlapping PCR. Short guide sequences can be cloned between the *Bsm*BI restriction sites as previously described^50^. Cas9-only and sgRNA-only derivatives were constructed as shown in Supplementary Fig. S2. All plasmids have been deposited with, and their sequence verified by, Addgene (see Supplementary Table S2 for Addgene reference numbers or addgene.org/Brett_Stringer/). Instructions for using the lentiCRISPRv2 plasmids are as described by the Zhang laboratory (https://media.addgene.org/cms/filer_public/53/09/53091cde-b1ee-47ee-97cf-9b3b05d290f2/lenticrisprv2-and-lentiguide-oligo-cloning-protocol.pdf).

### Lentivirus production

Lentivirus was produced in 10 cm diameter petri dishes from 70–80% confluent HEK293FT cells transfected with 3 μg lentiviral plasmid, 2 μg pVSV-G (Addgene #8454) and 5 μg psPAX2 (Addgene #12260), using 72 μl of lipofectamine 2000 in 3 ml of optiMEM medium. Opti-MEM was added to a final volume of 8 ml, containing 25 μM chloroquine, 1 × L-glutamine, 1 × non-essential amino acids, 1 × sodium pyruvate and 10% foetal bovine serum. The transfection medium was replaced the next day with 10 ml Opti-MEM supplemented with 1 × L-glutamine, 1 × non-essential amino acids and 1 × sodium pyruvate. Lentiviral supernatant was harvested 24 hours later, filtered through a 0.4 μm syringe filter and stored as 1 ml aliquots at −80 °C.

### Cell transduction

GBM cell lines were transduced with lentivirus in matrigel-coated 6-well tissue culture plates in 4 ml of RHB-A medium by adding 1 ml of unconcentrated lentivirus and 1 μg/ml polybrene followed by centrifugation at room temperature for 45 minutes at 440 g. Virus-containing medium was replaced next day with fresh RHB-A medium and antibiotic selection commenced 48 hours after lentiviral transduction began.

### GBM xenograft models

GBM xenograft models were initiated in 5-week old NOD/SCID mice housed under pathogen-free conditions. For intracranial cell injections 150,000 cells in 2 μl of 10 ng/ml laminin in PBS were injected 3 mm below the brain surface, 1.6 mm rostral of the bregma and 0.8 mm right of the midline, using a 25G Hamilton needle and a 2 μl syringe in a stereotaxic frame. Mice were anaesthetised with 2% isoflurane (Abbott) in oxygen at a flow rate of 0.8 litres per minute and given 100 μg of carprofen (Pfizer) subcutaneously for analgesia. The burr hole through which the cells were injected was sealed with bone wax and the midline scalp incision was closed with Vetbond (3M) tissue adhesive. Mice were euthanased when they exhibited signs of significant morbidity (hunching, weight loss, rough coat, ataxia, head tilt, paralysis). All studies were conducted according to protocols approved by the Animal Ethics Committee of the QIMR Berghofer Medical Research Institute. The “Principles of laboratory animal care” (NIH publication No. 86–23, revised 1985) were followed as well as the “Australian code of practice for the care and use of animals for scientific purposes”, 8^th^ edition 2013 and the “Queensland Animal Care and Protection Act 2001”.

### Bioluminescence imaging of xenograft tumours

Bioluminescence imaging of intracranial xenograft tumours was performed 5 minutes after injecting 0.5 mg of luciferin in 100 μl of DPBS (Dulbecco’s Phosphate-Buffered Saline, without Mg_2_^+^ and Ca_2_^+^) intraperitoneally into isoflurane-anaesthetised NOD/SCID mice using an IVIS Spectrum *In Vivo* Imaging System (Perkin Elmer).

### Exome sequencing

Genomic DNA was prepared from cultured cells using a QIAmp DNA mini kit (Qiagen) with RNase A treatment. Exome sequencing was performed by the Beijing Genomics Institute (BGI) using Truseq (BAH1 and WK1) or SureSelect (the remainder) exon capture and Illumina HiSeq 2000 technology. Data filtering, read alignment, SNV calling, annotation and statistics were performed by BGI.

### Copy number determination

Where investigated, gene copy number was determined by inspection of exon reads using Integrative Genomics Viewer^51,52^.

### Microarray analysis of gene expression

Microarray analysis of gene expression was performed using HumanHT-12_v4_BeadChip arrays (Illumina). RNA was extracted from patient tumour tissue, adherent cell cultures and xenograft tumours using RNeasy mini kits (Qiagen). Biotinylated cRNA was prepared using TotalPrep RNA amplification kits (Ambion). Microarray hybridisation and Cy3-streptavidin labelling was performed according to recommended protocols (Illumina). BeadChips were imaged with an iScan beadchip reader (Illumina) running BeadStudio software (Illumina). Raw data was exported to GenomeStudio (Agilent) where it was quantile normalised and background subtracted. Illumina beadchips allow a detection *P* value to be calculated as an estimate of gene measurements relative to background. *P* < 0.05 for all samples on a beadchip for a given probe was used as a cutoff to compile gene expression datasets. Where genes were represented by multiple probes, the highest value probe was used for gene-level summaries (tumour 11,267 genes; cell lines 10,435 genes; xenografts 7,079 genes). Illumina idat files are available for download (qimrberghofer.edu.au/q-cell/) and GenomeStudio-processed probe-level datasets (Supplementary Data S3) are provided for independent validation of our findings and as a reference for gene expression measurement.

### GBM molecular subtyping

GBM molecular subtyping was performed by hierarchical cluster analysis using MeV (http://mev.tm4.org) with gene sets reported for proneural, classical and mesenchymal GBM^4^, a Pearson distance metric and average linkage criteria, and also by gene set enrichment analysis^53,54^ (GSEAv2.2.4) in the pre-ranked mode using the proneural, classical and mesenchymal.rnk files in the Molecular Signatures Database v6.0. Gene ranking was according to z-scores for RNA expression determined by microarray analysis. Heatmaps were created using Excel.

### RNAseq

RNA was extracted from frozen cell pellets using a Qiagen AllPrep DNA/RNA Minikit (Cat No: 80204). RNA integrity was assessed using an Agilent RNA 6000 Eukaryote Total RNA kit (Cat. No: 5067–1511) and Bioanalyzer 2100 instrument. All RNA samples had an RNA Integrity Number (RIN) >9.0. RNA libraries were prepared using an Illumina TruSeq Stranded mRNA Library Preparation Kit Set A (Cat. No: RS-122-2101). RNA libraries were sequenced using an Illumina NexSeq® 500/550 High Output Kit v2 (150 cycles) (Cat. No: FC-404-2002) and NexSeq® 550 instrument.

Paired-end fastq files had adapters trimmed and flanking “N” bases removed for each paired-end read using Cutadapt v1.18. Trimmed files were aligned to the human GRCh37_ICGC_standard_v2 reference using STAR v2.5.2a. Quality control was performed using RNA-SeQC v1.1.8. Expected read counts and FPKM were calculated using RSEM v1.2.30, with a forward probability of 0 as the reads were stranded and derived from reverse strand. Finally, expected counts and FPKM for both genes and isoforms, were matched to gene/transcript annotation information using in-house scripts to provide expected counts and FPKM summary files for each sample.

### Western blotting

Cells (~70–90% confluent, passage 15) were washed 3 × in ice-cold PBS, then lysed on ice with ice-cold RIPA buffer (500 μl per plate, ThermoFisher Scientific) containing cOmpleteTM, Mini, EDTA-free Protease Inhibitor Cocktail (Merck) and PhosSTOP (Merck). Cells were disrupted by repeated passage through a 27G needle and crude lysates centrifuged at 20,000 g for 15 mins at 4 °C. Supernatants were collected, and protein content determined by Bio-Rad Protein Assay. 80 μg of total protein was loaded for each cell line per lane and proteins separated on 8–16% Criterion^TM^ TGX^TM^ Precast Midi Protein Gels (12 + 2 well, 45 μl, Bio-Rad) and transferred onto Immun-Blot® PVDF Membrane (Bio-Rad). Membranes were blocked in 5% BSA/TBST, washed in TBST, and protein levels determined using the following antibodies: EGFR (Abcam, ab52894, 1:1000), c-myc (CST, #5605, 1:1000), PTEN (CST, #9188, 1:1000) and β-actin (CST, #3700, 1:3000).

### Proteomics

GBM cell lines were grown as tumourspheres for three days. Cells were lysed in buffer containing 2% SDS, 100 mM Tris pH 7.6, protease and phosphatase inhibitors followed by precipitation with acetone. The protein precipitate was resuspended in 8 M urea, followed by reduction with DTT, alkylation with iodoacetamide and digested with trypsin using a standard protocol (https://www.nature.com/articles/ni.3693). Peptides were desalted and separated on a C18 analytical column (Waters) interfaced to an Orbitrap Elite mass spectrometer (Thermo Fisher Scientific). All raw files were searched against human proteome sequences from UniProt using the based label-free quantification algorithm in MaxQuant (https://www.ncbi.nlm.nih.gov/pmc/articles/PMC4159666/) (protein and peptide FDR = 0.01). Proteins of interest were extracted manually from the total list.

### Flow cytometry

Flow cytometry was performed with an LSR Fortessa (BD Biosciences) for data acquisition and fluorochrome-conjugated antibodies targeting CD133 (Miltenyi Biotec, AC133, 1:20), CD15 (BD Biosciences, 1:100), CD44 (in-house Hermes 3, 1:500), CD49f (Jomar Biosciences, 14-0495, 1:100), EGFR (in-house, M528, 0.5 μg/ml), EGFRvIII (in-house, 806, 0.5 μg/ml), PDGFRα (R&D Systems, MAB1264, 0.5 μg/ml), EphA2 (in-house, 1F7, 0.5 μg/ml), EphA3 (in-house, IIIA4, 0.5 μg/ml), EphA4 (in-house, 1F9, 0.5 μg/ml), FGFR1 (Abcam, ab823, 1:100), FGFR2 (Abcam, ab89476, 1:100), FGFR3 (R&D Systems, MAB766, 0.5 μg/ml), c-Met (R&D Systems, MAB3582, 0.5 μg/ml), Sox2 (R&D Systems, MAB2018, 0.5 μg/ml), Nestin (R&D Systems, MAB1259, 0.5 μg/ml), GFAP (Dako, Z0334, 1:100), β-III tubulin (Promega, G7121, 1:100) and MBP (Sigma Aldrich, M3821, 1:100). Cells were incubated with antibody on ice for 30 minutes in PBS containing 2% FBS. Where required, cells were fixed and permeabilised using Intracellular Fixation & Permeabilization Buffer (eBioscience) and stained with antibodies in 1 × Permeabilization Buffer. Data analysis was performed using Flowjo (Treestar) software.

### Statistical analysis

Unless otherwise noted, data are presented as the mean and standard error of the mean. GSEA reports normalised enrichment scores with the associated false discovery rate and familywise-error rate. *P* < 0.05 was considered statistically significant.

### Materials availability

All GBM cell lines are available to research groups with a standard materials transfer agreement and can be obtained by contacting the corresponding author or via the website, qimrberghofer.edu.au/q-cell/. Cell lines (not expressing luciferase) will be distributed from passage 10 onwards. Lentiviral plasmids can be obtained from Addgene at https://www.addgene.org/Brett_Stringer/.

## Supplementary information

Supplementary Figures S1-4

Supplementary Tables S1-2

Dataset 1

Dataset 2

Dataset 3

Dataset 4

Dataset 5

Dataset 6

## Data Availability

The datasets generated during the current study are available for download at qimrberghofer.edu.au/q-cell/ and/or as Supplementary Data S1–6. Microarray data have been deposited with the GEO repository with the series accession number GSE118793. RNAseq raw data is available for download from the NCBI sequence read archive with SRA accession number PRJNA508446.
