# Publisher Correction: A reference collection of patient-derived cell line and xenograft models of proneural, classical and mesenchymal glioblastoma

**DOI:** 10.1038/s41598-020-57850-w

**Published:** 2020-01-21

**Authors:** Brett W. Stringer, Bryan W. Day, Rochelle C. J. D’Souza, Paul R. Jamieson, Kathleen S. Ensbey, Zara C. Bruce, Yi Chieh Lim, Kate Goasdoué, Carolin Offenhäuser, Seçkin Akgül, Suzanne Allan, Thomas Robertson, Peter Lucas, Gert Tollesson, Scott Campbell, Craig Winter, Hongdo Do, Alexander Dobrovic, Po-Ling Inglis, Rosalind L. Jeffree, Terrance G. Johns, Andrew W. Boyd

**Affiliations:** 1 QiMR Berghofer Medical Research institute, Brisbane, Australia; 2 Royal Brisbane and Women’s Hospital, Brisbane, Australia; 3 Olivia Newton-John Cancer and Wellness Centre, Melbourne, Australia; 4 The University of Queensland, Brisbane, Australia; 5 Hudson institute of Medical Research, Clayton, Victoria Australia

Correction to: *Scientific Reports* 10.1038/s41598-019-41277-z, published online 20 March 2019

The version of this Article previously published contained lower resolution images for Figures 1, 2, 3, 4 and 5. This has now been corrected in the PDF and HTML versions of the paper.

