# Supplementary material for: A reference collection of patient-derived cell line and xenograft models of proneural, classical and mesenchymal glioblastoma
Source: Sci Rep. 2019 Mar 20;9:4902. doi: 10.1038/s41598-019-41277-z (PMC6427001; doi:10.1038/s41598-019-41277-z)
Supplement: Supplementary file 1 — Supplementary Figures S1-4 [file 41598_2019_41277_MOESM1_ESM.pdf]

A reference collection of patient-derived cell line and xenograft models of proneural, classical and mesenchymal glioblastoma

Brett W Stringer[1,*], Bryan W Day[1,*], Rochelle C J D'Souza[1], Paul R Jamieson[1], Kathleen S Ensbey[1], Zara C Bruce[1], Yi Chieh Lim[1], Kate Goasdoué[1], Carolin Offenhäuser[1], Seçkin Akgül[1], Suzanne Allan[1,2], Thomas Robertson[2], Peter Lucas[2], Gert Tollesson[2], Scott Campbell[2], Craig Winter[2], Hongdo Do[3], Alexander Dobrovic[3], Po-Ling Inglis[1,2], Rosalind L Jeffree[2,4], Terrance G Johns[5,+], Andrew W Boyd[1,4,+]

[1]QIMR Berghofer Medical Research Institute, Brisbane, Australia.
[2]Royal Brisbane and Women's Hospital, Brisbane, Australia.
[3]Olivia Newton-John Cancer and Wellness Centre, Melbourne, Australia.
[4]The University of Queensland, Brisbane, Australia.
[5]Hudson Institute of Medical Research, Clayton, Victoria, Australia.

[*]Joint first authors.
[+]Joint senior authors.

Correspondence should be addressed to B.W.S. (brett.w.stringer@gmail.com).

a

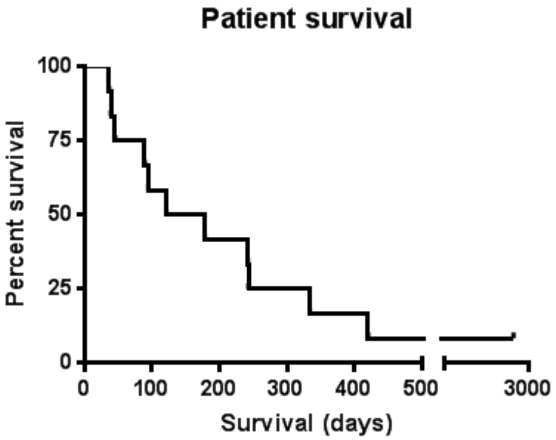

b

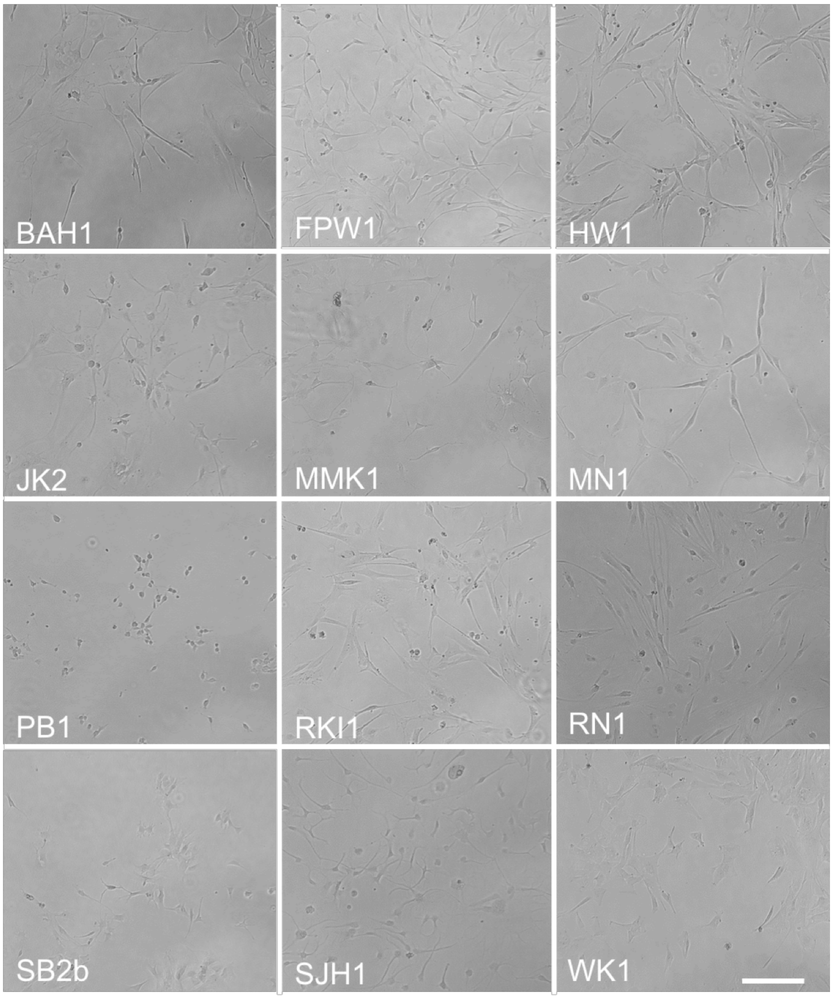

Supplementary Figure S1. Patient survival and morphology of low-passage, serum-free, patient-derived GBM cell lines. **(a)** Kaplan-Meier survival analysis of patients from whose tumours the GBM cell lines were established. **(b)** Bright field images of serum-free, adherent cultures of the GBM cell lines illustrating heterogeneity within as well as between the lines. Scale bar, 100 μm.

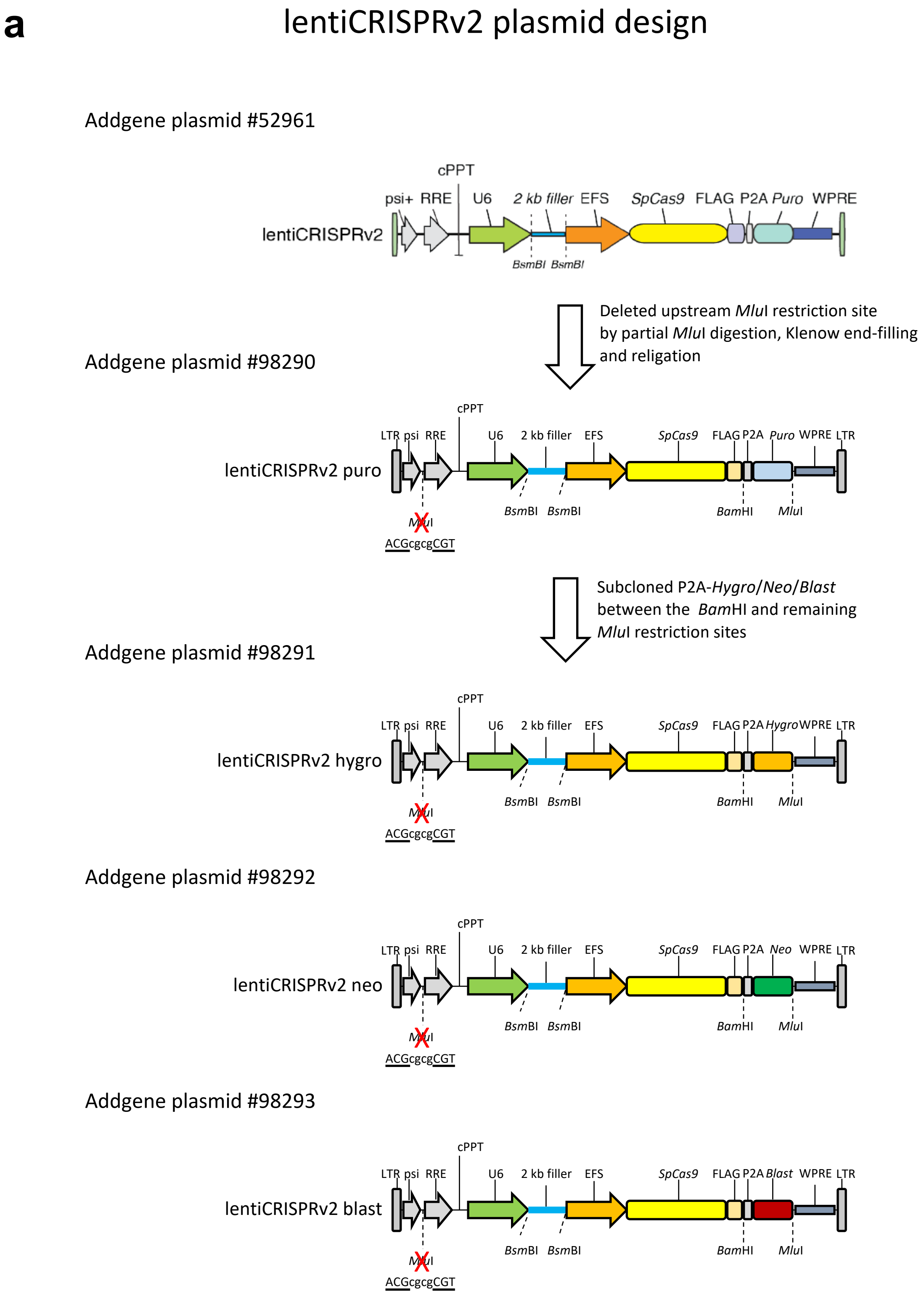

Supplementary Figure S2. Construction of lentiviral CRISPR/Cas9 plasmids. All plasmids were derived from lentiCRISPRv2 as shown after deleting the first of two *Mlu*I restriction sites. **(a)** Construction of the lentiCRISPRv2 series. **(b)** Construction of the lenti-sgRNA and lenti-SpCas9 series.

b

lenti-sgRNA and lenti-SpCas9 plasmid design

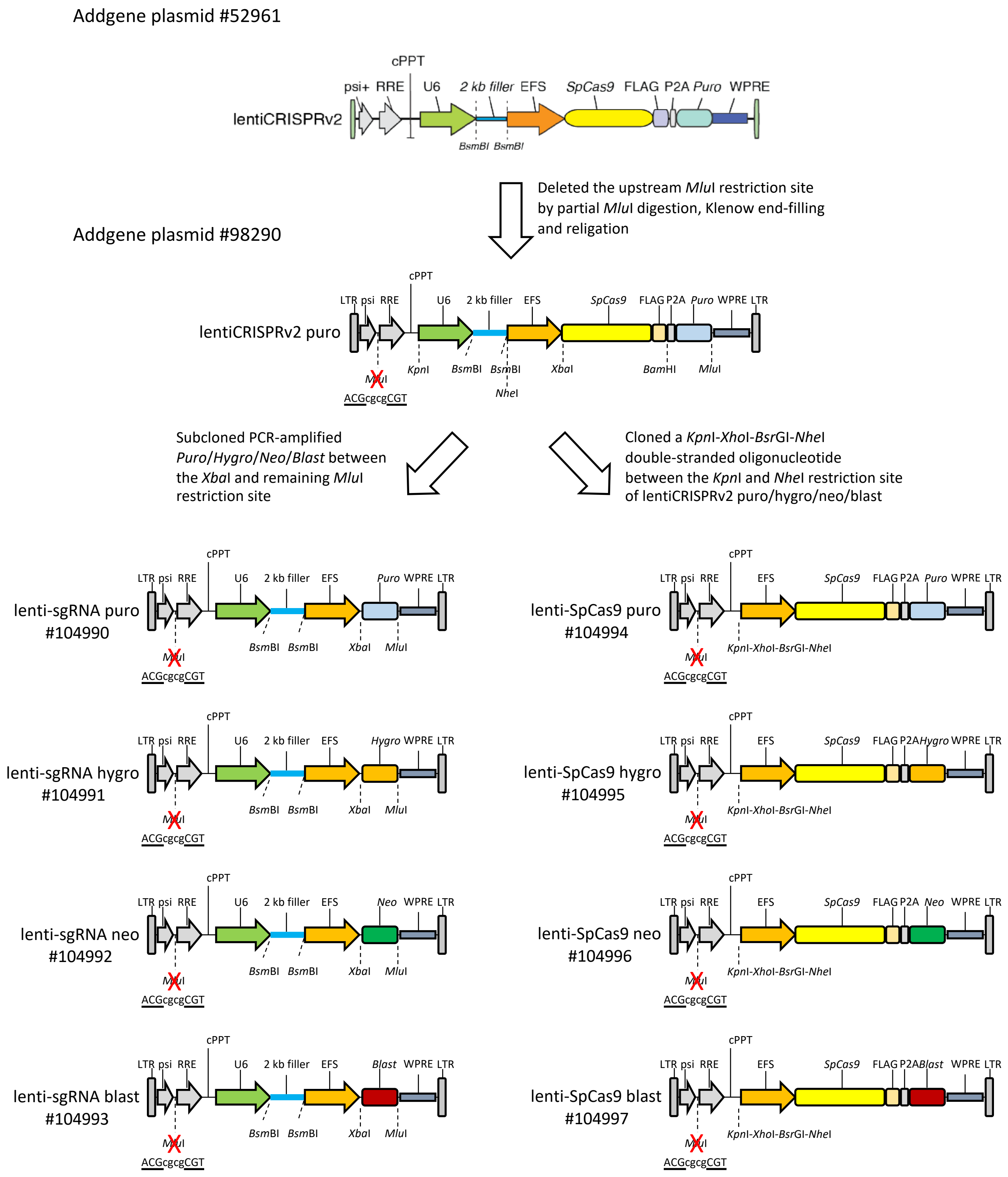

Supplementary Figure S2. (Cont.)

a Patient tumours

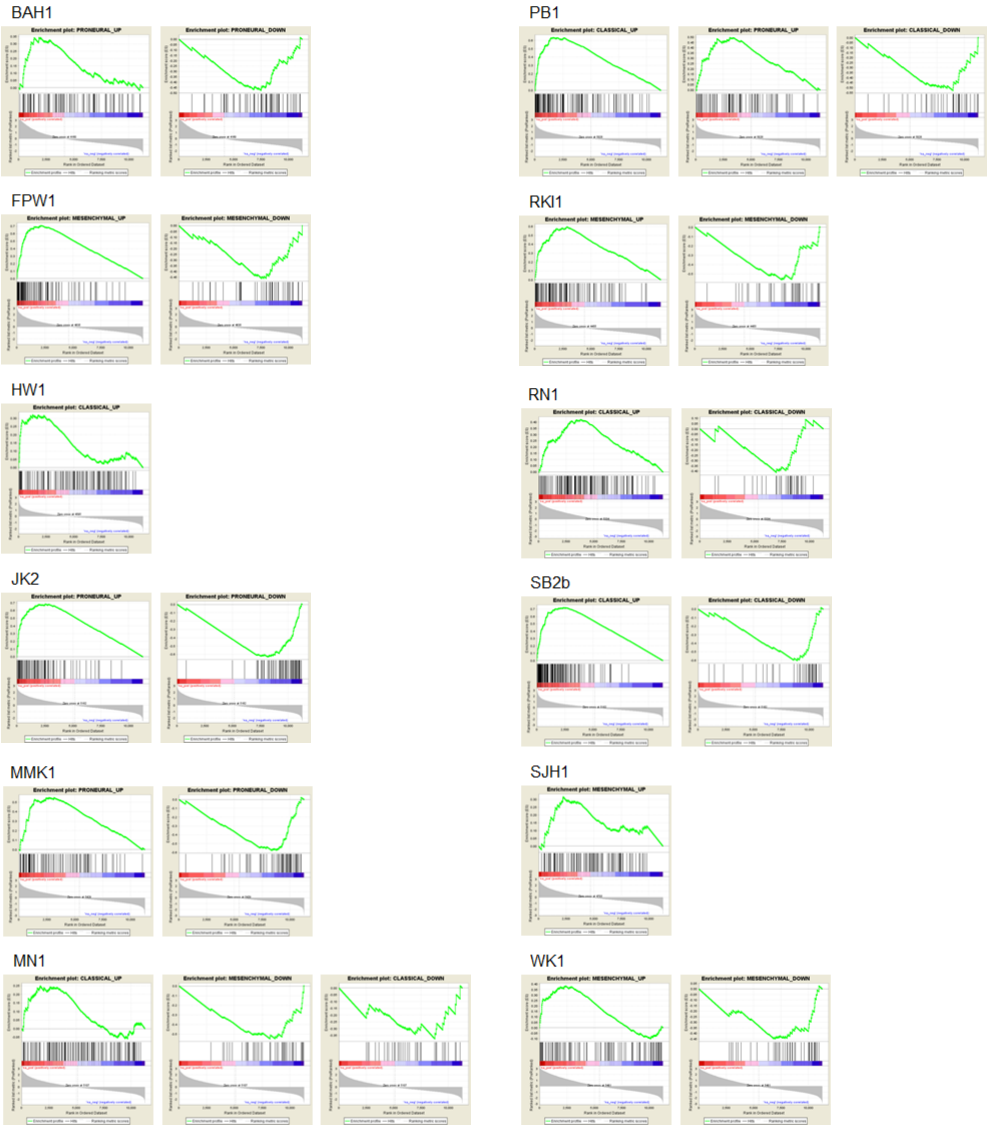

Gene set enrichment analysis				
Patient tumour	GBM subtype	NES	FDR q-value	FWER p-Value
BAH1	PN	2.165	0.000	0.000
FPW1	MES	3.062	0.000	0.000
HW1	CL	1.501	0.036	0.067
JK2	PN	2.901	0.000	0.000
MMK1	PN	2.581	0.000	0.000
MN1	MES/CL	2.199/1.482	0.000/0.017	0.000/0.026
PB1	CL/PN	2.810/2.053	0.000/0.000	0.000/0.001
RKI1	MES	2.658	0.000	0.000
RN1	CL	1.912	0.000	0.001
SB2b	CL	3.119	0.000	0.000
SJH1	MES	1.384	0.054	0.257
WK1	MES	1.907	0.005	0.002

Abbreviations: NES, normalised enrichment score; FDR, false discover rate; FWER, family-wise error rate;
CL, Classical; MES, Mesenchymal; PN, Proneural.

Supplementary Figure S3. Molecular subtyping by gene set enrichment analysis of patient tumours, GBM cell lines and xenografts initiated by the GBM cell lines. Molecular subtyping as proneural, classical or mesenchymal GBM by gene set enrichment analysis of matched **(a)** patient tumours, **(b)** derived GBM cell lines and **(c)** xenograft tumours initiated by the GBM cell lines following intracranial injection into NOD/SCID mice. Subtyping results along with the associated normalised enrichment scores and false discovery and error rates are summarised in the accompanying tables.

b GBM cell lines

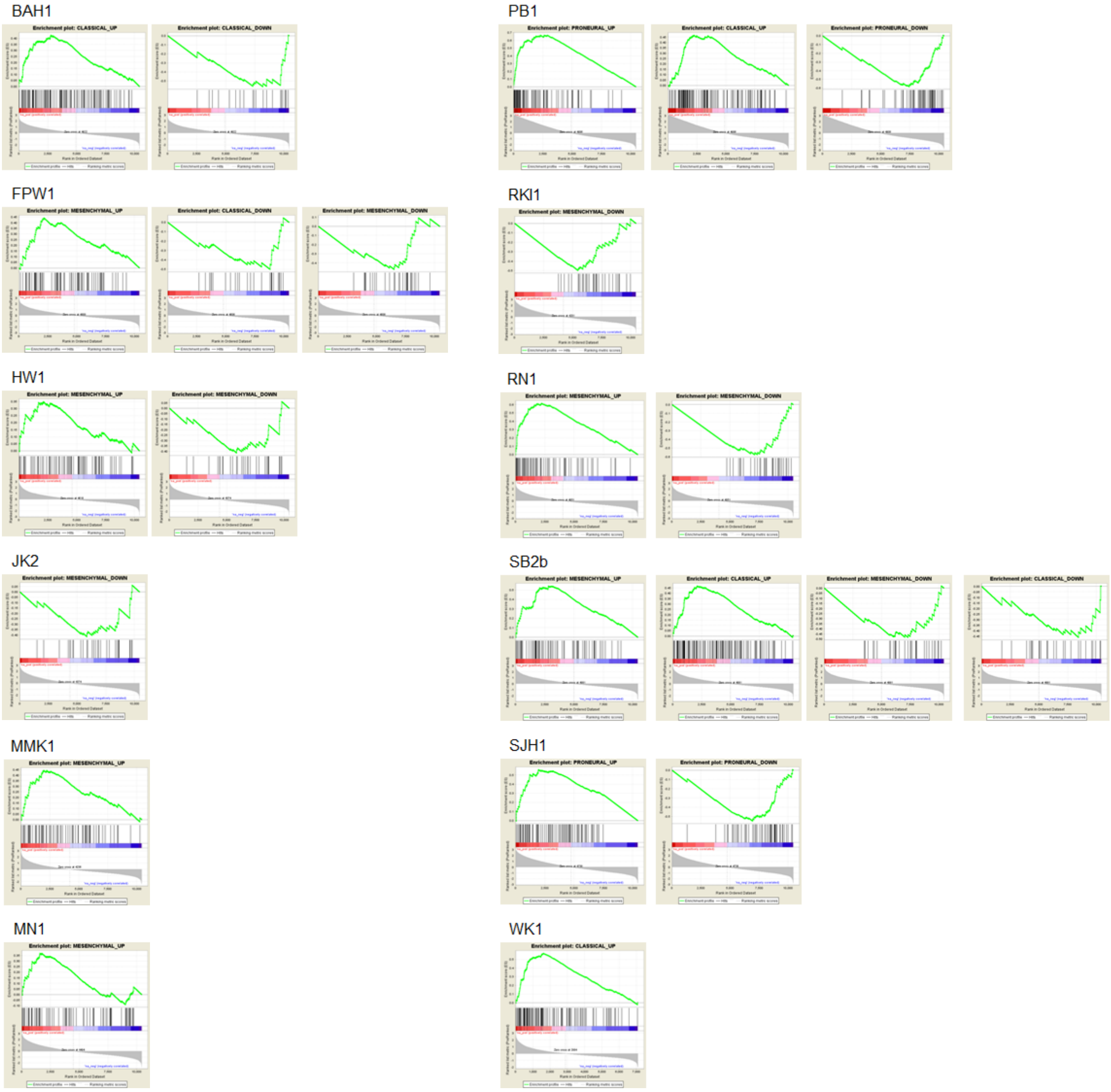

Gene set enrichment analysis				
**Primary GBM cell line**	**GBM subtype**	**NES**	**FDR q-value**	**FWER p-Value**
BAH1	CL	1.975	0.002	0.001
FPW1	MES/CL	1.749/1.822	0.001/0.003	0.005/0.003
HW1	MES	1.389	0.133	0.265
JK2	MES	1.563	0.024	0.039
MMK1	MES	1.851	0.005	0.005
MN1	MES	1.516	0.044	0.116
PB1	PN/CL	2.876/1.959	0.000/0.000	0.000/0.001
RKI1	MES	1.943	0.003	0.002
RN1	MES	2.690	0.000	0.000
SB2b	MES/CL	2.087/1.929	0.000/0.001	0.001/0.003
SJH1	PN	2.479	0.000	0.000
WK1	CL	2.383	0.000	0.000

Abbreviations: NES, normalised enrichment score; FDR, false discover rate; FWER, family-wise error rate;
CL, Classical; MES, Mesenchymal; PN, Proneural.

Supplementary Figure S3 (cont)

c Xenografts

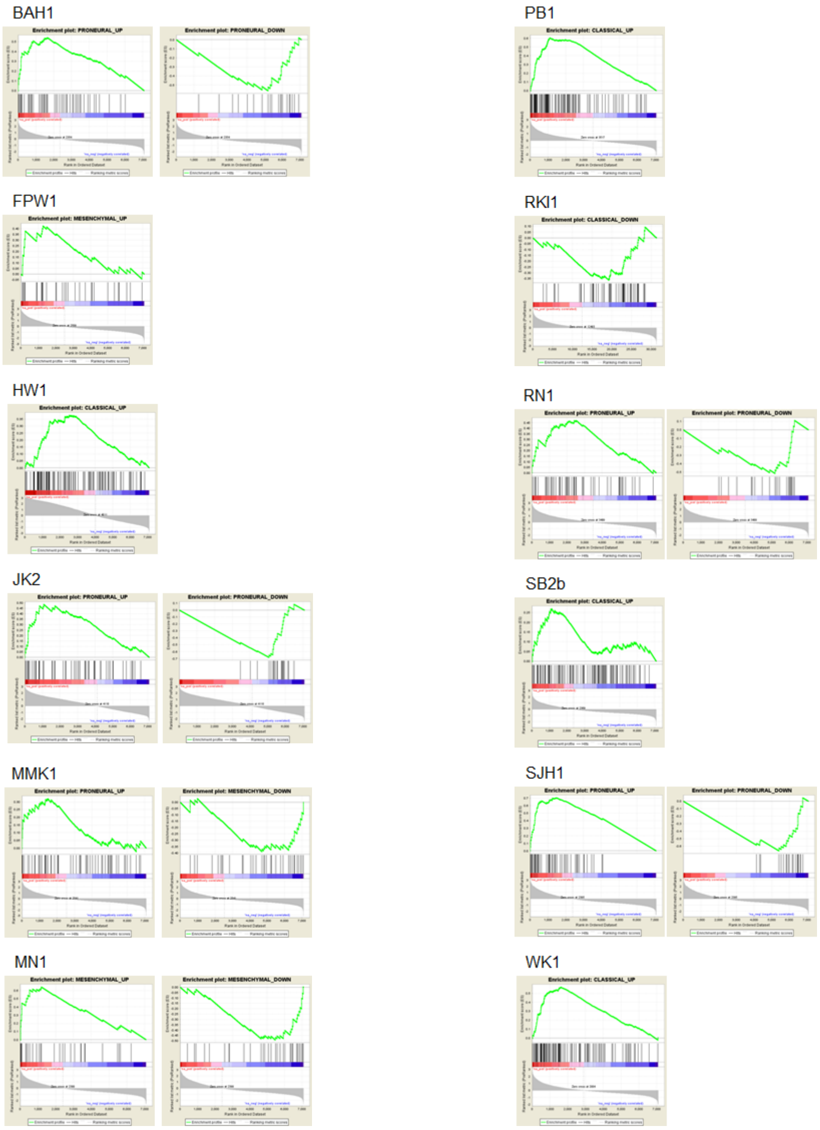

Gene set enrichment analysis				
Xenograft	GBM subtype	NES	FDR q-value	FWER p-Value
BAH1	PN	2.363	0.000	0.000
FPW1	MES	1.506	0.038	0.107
HW1	CL	1.541	0.075	0.092
JK2	PN	2.761	0.000	0.000
MMK1	PN/MES	1.312/1.508	0.112/0.076	0.386/0.081
MN1	MES	2.386	0.000	0.001
PB1	CL	2.205	0.000	0.000
RKI1	CL	1.502	0.049	0.037
RN1	PN	1.773	0.003	0.004
SB2b	CL	1.369	0.110	0.249
SJH1	PN	2.969	0.000	0.000
WK1	CL	2.316	0.000	0.000

Abbreviations: NES, normalised enrichment score; FDR, false discover rate; FWER, family-wise error rate;
CL, Classical; MES, Mesenchymal; PN, Proneural.

Supplementary Figure S3 (cont)

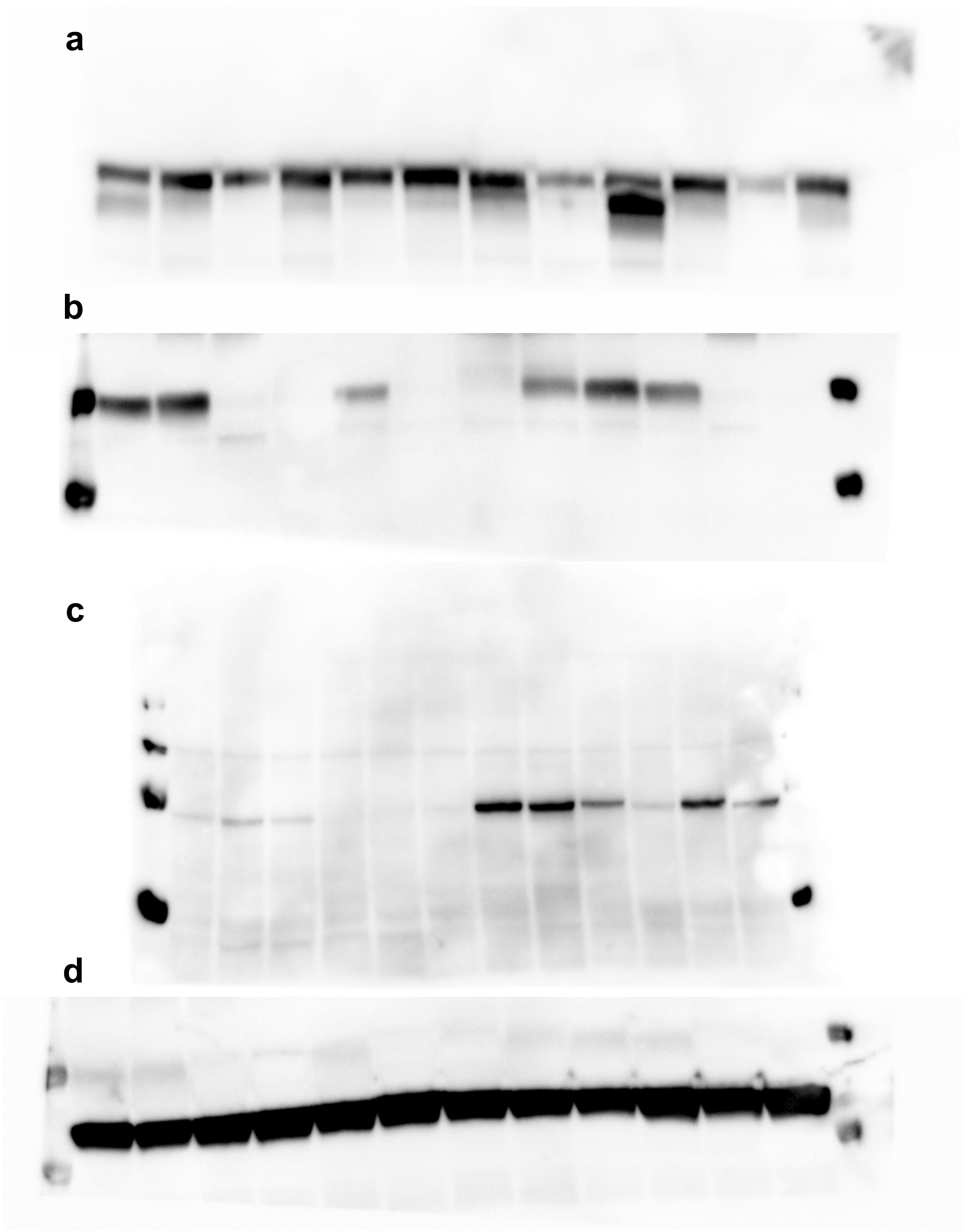

Supplementary Figure S4. Full length western blots for Figure 5. **(a)** Egfr, **(b)** PTEN, **(c)** c-myc, **(d)** β-actin.
