# Supplementary material for: A reference collection of patient-derived cell line and xenograft models of proneural, classical and mesenchymal glioblastoma
Source: Sci Rep. 2019 Mar 20;9:4902. doi: 10.1038/s41598-019-41277-z (PMC6427001; doi:10.1038/s41598-019-41277-z)
Supplement: Supplementary file 2 — Supplementary Tables S1-2 [file 41598_2019_41277_MOESM2_ESM.pdf]

A reference collection of patient-derived cell line and xenograft models of proneural, classical and mesenchymal glioblastoma

Brett W Stringer[1,*], Bryan W Day[1,*], Rochelle C J D'Souza[1], Paul R Jamieson[1], Kathleen S Ensbey[1], Zara C Bruce[1], Yi Chieh Lim[1], Kate Goasdoué[1], Carolin Offenhäuser[1], Seçkin Akgül[1], Suzanne Allan[1,2], Thomas Robertson[2], Peter Lucas[2], Gert Tollesson[2], Scott Campbell[2], Craig Winter[2], Hongdo Do[3], Alexander Dobrovic[3], Po-Ling Inglis[1,2], Rosalind L Jeffree[2,4], Terrance G Johns[5,+], Andrew W Boyd[1,4,+]

[1]QIMR Berghofer Medical Research Institute, Brisbane, Australia.
[2]Royal Brisbane and Women's Hospital, Brisbane, Australia.
[3]Olivia Newton-John Cancer and Wellness Centre, Melbourne, Australia.
[4]The University of Queensland, Brisbane, Australia.
[5]Hudson Institute of Medical Research, Clayton, Victoria, Australia.

[*]Joint first authors.
[+]Joint senior authors.

Correspondence should be addressed to B.W.S. (brett.w.stringer@gmail.com).

Supplementary Table S1. Short tandem repeat profiles of GBM cell lines

Cells	TH01	D21S11	D5S818	D13S317	D7S820	D16S539	CSF1PO	AMEL	vWA	TPOX
BAH1	6,8	30	11	12,14	9,11	9,11	10,11	X	17	8,11
FPW1	9.3	27,30	13	12	11	9,11	11	X,Y	16,17	8
HW1	6,9	28,29	11,12	12	8,10	13	10,12	X	15,18	8
JK2	7,9	28	10,11	8,11	8	12	12	X,Y	15,20	8,11
MMK1	6,10	27,33.2	12	13	9,10	11,13	10,11	X	16,17	8
MN1	6	29,31.2	12,13	8	9,12	12,13	12	X	17	10,11
PB1	7,9	30	10,11	12	9,11	12	11,13	X,Y	17	8
RKI1	7,8	29,33.2	12,13	8	10,12	11,12	10	X	16	8
RN1	6,9.3	29	12	11,12	9	9	11,12	X,Y	14,18	8,11
SB2b	6,7	28,32	11,13	12	11,12	9,12	10	X,Y	17	9,10
SJH1	8,9.3	29	12,13	11	11	11,12	10,13	X,Y	15	8
WK1	6,9.3	27,28	D5S818	12,13	8,11	11,12	10,11	X,Y	16,18	8

Abbreviations: STR=short tandem repeat. Profiles comprise the alleles at 10 STR loci. No significant match (>80% relatedness) was found when compared with profiles in the ATCC cell database (https://www.atcc.org/STR_Database.aspx).

Supplementary Table S2. Lentiviral plasmids deposited with Addgene

Plasmid name	Addgene reference	Use	Antibiotic resistance
pF CAG luc puro	67501	gene expression	puromycin
pF CAG luc hygro	67502	gene expression	hygromycin
pF CAG luc IRES neo	98294	gene expression	G418
pF CAG BFP2 IRES neo	108175	fluorescent labelling	G418
lentiCRISPRv2 puro	98290	CRISPR/Cas9 cutting	puromycin
lentiCRISPRv2 hygro	98291	CRISPR/Cas9 cutting	hygromycin
lentiCRISPRv2 neo	98292	CRISPR/Cas9 cutting	G418
lentiCRISPRv2 blast	98293	CRISPR/Cas9 cutting	blasticidin S
lenti-sgRNA puro	104990	sgRNA expression	puromycin
lenti-sgRNA hygro	104991	sgRNA expression	hygromycin
lenti-sgRNA neo	104992	sgRNA expression	G418
lenti-sgRNA blast	104993	sgRNA expression	blasticidin S
lenti-SpCas9 puro	104994	Cas9 expression	puromycin
lenti-SpCas9 hygro	104995	Cas9 expression	hygromycin
lenti-SpCas9 neo	104996	Cas9 expression	G418
lenti-SpCas9 blast	104997	Cas9 expression	blasticidin S
