# Supplementary material for: A reference collection of patient-derived cell line and xenograft models of proneural, classical and mesenchymal glioblastoma
Source: Sci Rep. 2019 Mar 20;9:4902. doi: 10.1038/s41598-019-41277-z (PMC6427001; doi:10.1038/s41598-019-41277-z)
Supplement: Supplementary file 3 — Dataset 1 [file 41598_2019_41277_MOESM3_ESM.pdf]

A reference collection of patient-derived cell line and xenograft models of proneural, classical and mesenchymal glioblastoma

Brett W Stringer[1,*], Bryan W Day[1,*], Rochelle C J D'Souza[1], Paul R Jamieson[1], Kathleen S Ensbey[1], Zara C Bruce[1], Yi Chieh Lim[1], Kate Goasdoué[1], Carolin Offenhäuser[1], Seçkin Akgül[1], Suzanne Allan[1,2], Thomas Robertson[2], Peter Lucas[2], Gert Tollesson[2], Scott Campbell[2], Craig Winter[2], Hongdo Do[3], Alexander Dobrovic[3], Po-Ling Inglis[1,2], Rosalind L Jeffree[2,4], Terrance G Johns[5,+], Andrew W Boyd[1,4,+]

[1]QIMR Berghofer Medical Research Institute, Brisbane, Australia.
[2]Royal Brisbane and Women’s Hospital, Brisbane, Australia.
[3]Olivia Newton-John Cancer and Wellness Centre, Melbourne, Australia.
[4]The University of Queensland, Brisbane, Australia.
[5]Hudson Institute of Medical Research, Clayton, Victoria, Australia.

[*]Joint first authors.
[+]Joint senior authors.

Correspondence should be addressed to B.W.S. (brett.w.stringer@gmail.com).

BAH1 - Primary Glioblastoma

Pathology report:

HISTORY
Right frontal tumour for frozen section. No known primary. Contrast enhancing lesion. Cholecystectomy.

FROZEN SECTION REPORT
Right frontal tumour: The specimen consists of multiple tan tissue pieces in aggregate measuring 15×9×2mm. Representative section in Block (1A).
Frozen section diagnosis: Intermediate grade glioma.

MACROSCOPIC
1. Right frontal tumour (FS remnant): Specimen blocked in toto in block (1A).

2. Right frontal tumour (formalin): The specimen consists of multiple irregular tan and friable soft tissue pieces, which in aggregate measure 40×30×30mm.
Representative sections taken in blocks (2A) to (2C).

MICROSCOPIC
Paraffin sections show features of a malignant glioma that demonstrates a variety of morphologic growth patterns. In some areas the tumour shows relatively monotonous cytology with some perinuclear clearing however this is not accompanied by other features of oligodendroglioma and in other areas the tumour demonstrates a more typical astrocytic phenotype. Prominent areas of serpiginous palisaded tumour necrosis are present and there is evidence of vascular proliferation.

SUMMARY
Right frontal brain tumour: Glioblastoma multiforme; WHO grade IV.

FPW1 - Primary Glioblastoma

Pathology report:

HISTORY
?GBM.

MACROSCOPIC
1. The specimen jar is labelled **Brain tumour frozen section** and consists of two fragments of soft cream tissue measuring 15×10×6mm. Two smears prepared, one rep section for frozen section block (1A). Frozen section diagnosis: High-grade glioma; probably GBM.
Block (1A) 2TS all embedded.

2. The specimen jar is labelled **Brain tumour** and consists of multiple pieces of creamy tissue, the largest piece measuring 20×12×5mm.
Block (2A) – (2B) all embedded.

MICROSCOPIC
1. and 2. Brain tumour frozen section and brain tumour: The sections show a glial proliferation with large areas of necrosis, glomeruloid vascular hyperplasia and large atypical glial cells with frequent mitoses.

SUMMARY
1. and 2: Brain tumour frozen section and brain tumour: Glioblastoma multiforme.

HW1 - Primary Glioblastoma

Pathology report:

HISTORY
Frozen section: Right frontal/temporal lesion. History of expressive dysphasia.

MACROSCOPIC
1. The specimen jar is labelled **Right frontal parietal tumour** and consists of soft gelatinous tissue measuring 10mm in extent. Frozen section remanent (1A); remainder of the tissue blocked in toto in (1B).

Frozen section diagnosis: Malignant tumour, possibly metastatic malignancy.

2. The specimen jar is labelled **Right frontal parietal tumour** and consists of two grey pink soft pieces of tissue, the larger measuring 40×25×15mm and the smaller measuring 12×7×5mm. Reserved tissue blocked in (2A - 2F).

MICROSCOPIC
Right frontal/parietal tumour: The cerebral cortex tumour is a primary glial tumour showing the histomorphology of a glioblastoma multiforme. The tumour presents has a mass lesion with a relatively circumscribed margin. The tumour is not a metastatic malignancy as was suggested in the frozen section report.

SUMMARY
Right frontal/parietal tumour: Glioblastoma multiforme, WHO grade IV.

JK2 - Primary Glioblastoma

Pathology report:

HISTOLOGY
Clinical notes: 3/52 left weakness. 3/12 personality change. Heterogeneous right frontal tumour.

MACROSCOPIC
1. The container is labelled **R Frontal lesion.** The specimen consists of pale areas of tissue measuring 10×8×2mm for smear and frozen section.

Frozen section diagnosis: Necrosis with adjacent high grade glioma.

2. The second specimen is labelled **Right frontal tumour.** The specimen consists of a piece of brain measuring 60×55×50mm. The gyri look flattened and there are some excrescences on the surface. The cut surface looks largely necrotic and there are some clots within the brain parenchyma. There is also expansion of the white matter adjacent to the necrotic margin focally with thinning of the overlying grey matter and blurring of the white grey junction.

There is a separate piece of tissue in the pot measuring 20×10×6mm. (2A) contains the separate piece of tissue, (2B) – (2F) transverse slice with (2B) margin, (2C) cortex, (2D) margin, (2E) cortex and (2F) margin adjacent to cortex; (2G) – (2K) are a parallel section 1cm from the first transverse section; (2L) and (2M) sections taken perpendicular to this to demonstrate margins at the edge of the excision. Representative sections. Annotated diagram attached.

MICROSCOPIC
Paraffin sections confirm the frozen section diagnosis of a high grade glioma. The tumour exhibits features of a Glioblastoma multiforme. Areas with small cell morphology are present and there is prominent serpiginous palisaded necrosis and areas of vascular endothelial proliferation.

SUMMARY
Right frontal lobe resection: Glioblastoma multiforme: WHO grade IV.

MMK1 - Primary Glioblastoma

Pathology report:

HISTORY
Multiple request forms received.
No. 1: Histology frozen section please. Bone is an abnormal colour? Theatre 2.
No. 2: histology. There is also a CNS proforma attached to the back.
Presenting symptom: nausea, headache and fatigue 2/52. Clinical history: PHx: Cerebellar, atrophy or MS. (Dx uncertain). 40yrs – ataxia.
Imagining findings: Anterior right temporal ring enhancing lesion approximately 4×4×4cm.
Contrast enhancement: Present.
Current and previous treatment: Dexamethasone.
Anatomical site: Intra-axial: Temporal lobe.
Laterality of tumour: Right
Specimen type: resection.
Clinical or differential diagnosis: GBM, Met.

MACROSCOPIC
1. The specimen container is labelled **brain tumour:** the specimen consists of three pieces of 3mm wide pink tissue. One piece submitted for smear, two pieces for frozen section (1A). Specimen blocked in toto. Frozen section remnant all embedded block (1A).
Frozen section diagnosis no. 1: Large atypical cells: ?melanoma, ?lymphoma (?GBM)

2. The specimen container is labelled **abnormal bone:** The specimen consists of two fragments of tissue 15mm and 5mm wide. Macroscopically the tissue appears necrotic. Half of the specimen is submitted for frozen section (2A).
Frozen section diagnosis No. 2: Bone, possibly necrotic bone.
Frozen section remnant all embedded (2A); remainder of specimen 2 all embedded (2B). Wrapped and blocked.

3. The specimen container is labelled **brain tumour 2:** The specimen consists of several 5mm fragments of soft white-haemorrhagic tissue. Frozen section diagnosis NO.3: Suggestive of GBM.
Frozen section remnant wrapped and blocked in toto (3A): all remainder tissue in specimen pot 3 embedded in (3A) including frozen section remnant.

4. The specimen container is labelled **brain tumour**: The specimen consists of a single piece of irregularly shaped haemorrhagic soft tissue measuring 33×27×14mm. The external surface is inked blue. The specimen is entirely embedded as serial transverse sections (4A to 4D). Sectioning reveals a heterogeneous cut surface with firm cream areas interspersed with gelatinous areas.

5. The specimen container is labelled **right temporal lobe**: the specimen consists of two portions of tissue. The specimen weights 17.7g. One appears to represent a portion of external brain tissue with vascular channels, gyri and sulci. This part of the specimen measures 42×42mm by up to 23mm. The apposing surface is irregularly roughened and haemorrhagic. There is also a second small piece of tissue in the specimen container measuring 20×8×5mm. The smaller piece inked bisected, all embedded (5A), the larger piece of tissue is photographed, inked blue and divided into serial transverse sections and all embedded from one side to the other as per the photograph. What is possible false cavity margin is inked black (5B to 5L). (5E, 5F, 5I & 5G) each contain piece bisected. Refer to photograph for blocking information. Specimen has been entirely embedded.

6. The specimen container is labelled **abnormal bone**: The specimen consists of a single fragment of bone measuring 10x5x5mm. Specimen is inked and blocked in toto (6A).

MICROSCOPIC
1 – 4. Brain tumour: There is a high grade glial tumour composed of anaplastic, highly atypical round and spindly glial cells. Tumour cells show remarkable nuclear enlargement, nuclear hyperchromasia and nuclear pleomorphism together with increased mitotic rate, multi-nucleated tumour cells with bizarre nuclei and also karhyorectic cells. There is extensive coagulative tumour necrosis in areas with characteristic palisading necrosis. There is microvascular proliferation with glomeruloid structures. Tumour cells show positive reaction for GFAP and mild focal positivity for cytokeratin. The features are in keeping with glioblastoma multiforme (WHO Grade IV).

5. Right temporal lobe: the specimen is composed of brain tissue infiltrated by a Grade 2 diffused astrocytoma which shows transition to anaplastic astrocytoma (WHO Grade 3) and glioblastoma (WHO Grade 4). The later features suggest differentiation of a Grade 2 astrocytoma.

5. Abnormal bone: There is compact cortical bone with no evidence of tumour invasion.

SUMMARY
1 - 6. Brain tumour: Glioblastoma multiforme (WHO Grade IV).

MN1 - Primary Glioblastoma

Pathology report:

HISTORY
Two request forms and two frozen sections forms.

The first request form reads:
Frozen section L frontal lobe lesion. Presents with expressive dysphasia. The surgeon gives a history of colorectal carcinoma.

There is another request form which reads:
Formal histology left frontal lobe lesion. Presents with expressive dysphasia.

MACROSCOPIC
1. The first frozen section reads: Container labelled **Brain tumour**. The specimen consists of a piece of cream to pink soft tissue measuring 5×4×3mm. Smear x1 and remaining tissue for frozen section. (1A) frozen section remnant.

Frozen section diagnosis: Glioma, favour high grade.

2. There is a second frozen section form which reads: Container labelled **Brain lesion number 2.** The specimen consists of three to four pieces of cream to pink tissue measuring 2-3mm each. Smear x 1 and one piece of frozen section. (2A) frozen section remnant of specimen 2; (2B) remainder of the tissue in specimen 2.

Frozen section diagnosis: Glioma.

3. The specimen is labelled **Brain lesion** and consists of fragments of brain tissue measuring 13×12×10mm in aggregate. Wrapped and blocked in toto in (3A).

4. The specimen in labelled **Brain tumour deep margin** and consists of four fragments of pale tissue, in aggregate measuring 8x7x3mm. Wrapped and blocked in toto in (4A).

MICROSCOPIC
1. 2. & 3. Brain tumour: The paraffin sections confirm the frozen section diagnosis of high-grade diffusely infiltrating glioma. The tumour cells appear astocytic and are relatively monomorphous with focal perivascular orientation of tumour cells. There evidence of palisaded necrosis and frequent mitoses are present in some areas. Vascular endothelial proliferation is also present. The features are consistent with a glioblastoma.

4. Brain tumour deep margin: the sections show infiltration of the biopsy by glioma cells.

SUMMARY
Left frontal lobe brain tumour: Glioblastoma (WHO grade IV).

PB1 - Primary Glioblastoma

Pathology report:

HISTORY
Left frontal tumour for frozen section. Contrast enhancing. No primary renal transplant.

MACROSCOPIC
1. Brain tumour: The specimen consists of blood stained tissue fragments measuring 10mm in aggregate. Frozen section remnant (1A) transferred to (1A). Remaining tissue blocked in (1B).

Frozen section diagnosis: high grade glial tumour.

2. Brain tumour: the specimen consists of multiple fragments of grey/brown tissue measuring 40×25×4mm in aggregate. Wrapped and blocked in toto (2A-2B)

MICROSCOPIC
Paraffin sections confirm the frozen section diagnosis of high grade glial tumour with features of glioblastoma multiforme (WHO grade IV).

SUMMARY
Left frontal tumour: Glioblastoma multiforme (WHO grade IV).

RKI1 - Primary Glioblastoma

Pathology report:

HISTORY
Frozen section. Left temporal lobe tumour.

MACROSCOPIC
1. The specimen is labelled **Brain tumour**. The specimen consists of two fragments of cream to pink tissue measuring in aggregate 5×4×2mm. Two smears are prepared and one rep, section per piece for frozen section. Block (1A) frozen section remnants; (1B) remaining tissue. Specimen blocked in toto.

Frozen section diagnosis: High-grade glioma, haemorrhage/necrotic, bled into tumour clinically.

2. The specimen is labelled **Brain tumour**. The specimen consists of multiple fragments of cream to maroon tissue measuring in aggregate 20×10×5mm. The largest fragment measures 12×3×3mm. Blocks (2A) and (2B) specimen wrapped and blocked in toto.

MICROSCOPIC
1. Brain tumour: the paraffin sections confirm the frozen section diagnosis of a high grade glioma. There are large areas of coagulative necrosis which shows pseudo-palisading in one of the two fragments. There is crowding of pleomorphic glial cells with hyperchromatic irregular nuclei. There is focal endothelial proliferation.

2. Brain tumour: some of the fragments show diathermy artefact which limits interpretation, however the appearances are similar to those in Specimen 1 with crowding of pleomorphic glial cells with hyperchromatic irregular nuclei. There is a proliferation of blood vessels of small to medium diameter, some of which show endothelial proliferation. There are focal areas of haemorrhage and calcification, as well as areas of coagulative necrosis.

COMMENT: the appearances in both specimens are consistent with Glioblastoma multiforme, WHO Grade IV.

SUMMARY
1 & 2. Brain tumour: Glioblastoma multiforme, WHO Grade IV.

RN1 - Primary Glioblastoma

Pathology report:

HISTORY
Left temporal lesion.
Past medical history: Metastatic bowel adenocarcinoma.
? metastases. ? GBM.

MACROSCOPIC
1.The specimen is labelled **Brain tumour** and consists of three fragments of pink cream tissue measuring 6×3×3mm, 6×3×2mm and 3×2×2mm. One H&E smear from smallest fragment and one representative section each larger two fragments. GBM. (1A) frozen section remnant; (1B) remaining tissue blocked in toto.

2. The specimen is labelled **Brain tumour** and consists of multiple pieces of light tan tissue measuring 18×12×4mm in aggregate. (2A) specimen blocked in total.

MICROSCOPIC
1. and 2. Brain tumour and brain tumour: The sections show a densely cellular proliferation composed of medium-sized to large cells in a background of smaller oligodendrocyte-like cells and fibrillary stroma. There is vascular hyperplasia and areas of necrosis. There are frequent foci of vascular hyperplasia. Mitoses are frequent. There are some areas of degenerative tissue, but no real tumour necrosis.

SUMMARY
1. and 2. Brain tumour and brain tumour: the lesion is best regarded as a diffuse infiltrative malignant glioma.

Further studies (p53 and FISH) are pending to allow a better categorisation.

SUPPLEMENTARY REPORT
Thank you for the opportunity to review this case. In some areas this tumour demonstrates oligodendroglioma features with perinuclear halo formation, an arborescent vascular pattern and punctuate calcifications. However, other areas demonstrate astrocytic features with background fibrillar tumour cell processes, perivascular orientation and scattered bizarre giant cells. Frequent mitoses are present and there is evidence of vascular proliferation. Focal necrosis is identified at the edge of one biopsy fragment. Immunohistochemical staining for p53 is normal with only focal positive immunoreactivity.

SUMMARY
Left temporal brain biopsy: Glioblastoma multiforme with oligodendroglioma component; 1p/19q deletions not present; WHO grade IV.

SB2b - Recurrent Glioblastoma

Pathology report:

HISTORY
R parietal intrinsic brain lesion.
GBM resection in Feb 2010. Post op chemoradiation.

MACROSCOPIC
1. The specimen is labelled **Brain lesion**. The specimen consists of four haemorrhagic pieces of tissue measuring 15×5×4mm in aggregate. Smears are taken. 3RS for FS.
Frozen section diagnosis: Glioma, probably high grade.
(1A) frozen section remnant; (1B) remainder of tissue. Specimen blocked in toto.

2. The specimen is labelled **Brain lesion histo**. The specimen consists of eight pieces of cream tissue measuring in aggregate 50×20×12mm. (2A) 3TS; (2B)-(2C) 2TS; (2D) remainder of tissue. Specimen is blocked in toto.

MICROSCOPIC
1. & 2. Brain lesion: Paraffin sections show a high-grade glioma. It is a highly cellular tumour with cellular pleomorphism, mitotic activity and endothelial proliferation. There is sclerosis, haemosiderin deposition and foam cells consistent with treatment effects. The appearances are consistent with recurrent glioblastoma multiforme.

SUMMARY
Glioblastoma multiforme.

SJH1 - Primary Glioblastoma

Pathology report:

HISTORY
Frozen section left temporal lobe biopsy. Past history – SCC tongue. SCC lung. Histo, HSV PCR. Brain Bx + dural Bx. Past history SCC skin lesion. Left temporal lobe lesion. Reactive astrocytes with atypia. MRI ? glioma, ? HSV encephalitis.

MACROSCOPIC
1. The specimen is labelled **Brain tumour** and consists of two cores of tissue. One core is cream and measures 10×2×2mm. A second core is cream-red and measures 9×1×1mm. A smear is performed by samples from each end of core 1. The remainder of core 1 is submitted for frozen section. Block (1A) frozen section remnant; (1B) remaining tissue (second core).

Frozen section diagnosis: No evidence of metastatic SCC. Atypical and reactive astrocytes – consistent with edge of glial lesion.

2. The specimen is labelled **Brain tumour**. The specimen is received in formalin and consists of seven strips of cream to pink tissue measuring in aggregate 12×10×2mm. The largest strip of tissue measures 11×2×2mm. Block (2A) specimen wrapped and blocked in toto.

3. The specimen is labelled **Dura**. The specimen is received fresh and consists of a single fragment of pink tissue measuring 2×1×1mm. Block 3(A) specimen wrapped and blocked in toto.

Note: Specimens 2 & 3 – HSV PCR to be performed on paraffin embedded tissue after the histology sections are cut.

MICROSCOPIC
1. Brain tumour and 2. Brain tumour: The paraffin sections on the tissue cores show hypercellular cerebral tissue with the atypical astrocytes showing marked pleomorphorism with hyperchromatic nuclei and cells with multiple nuclei. In the second set of cores the hypercellularity is more marked and there are several atypical mitotic figures. Endothelial proliferation is not evident and definite necrosis is not apparent.

Comment: The appearance is at least that of an anaplastic astrocytoma (WHO III). Given the degree of anaplasia and the atypia of the mitoses this case will be reviewed.

3. Dura: This specimen consists of a small fragment of dense fibrous tissue consistent with dura. There is no evidence of inflammation or tumour.

SUMMARY
1. Brain tumour and **2. Brain tumour:** Anaplastic astrocytoma (WHO III); see above comment.

3. Dura: Non-inflamed dura.

ADDITIONAL REPORT
Thank you for the opportunity to review this case. I agree with the diagnosis of malignant astrocytic diffuse glioma. In some of the biopsy fragments of the tumour demonstrates marked increased cellularity and bizarre cytologic atypia. No vascular endothelial proliferation or necrosis is identified in the paraffin sections. However, some thickened blood vessels showing changes suspicious for vascular proliferation are seen in the intra-operative smear, suspicious for a Glioblastoma multiforme.

Please see subsequent biopsy report on this patient.

HISTORY
Formal histo. Left temporal lobe lesion. Bx anaplastic astro, WHO III

MACROSCOPIC

The specimen is labelled **Brain tumour.** The specimen consists of four separate pieces of brain tissue measuring 3×2×2mm, 4×2×2mm, 4x2x2mm, 4x2x2mm and up to 2x2x<1mm. The four pieces of tissue are wrapped and blocked in toto, (1A).

MICROSCOPIC
Brain tumour: Sections show a malignant diffuse glioma with astrocytic morphology. Frequent mitoses including atypical forms are present. In one of the fragments there is evidence of vascular endothelial proliferation but no necrosis is seen in the biopsy material.

COMMENT
Based on this material the tumour is best regarded as Glioblastoma multiforme.

SUMMARY
Left temporal lobe biopsy: Glioblastoma multiforme; WHO grade IV.

WK1 - Primary Glioblastoma

Pathology report:

HISTORY
Frozen section R parietal tumour, no known primary, contrast enhancing on MRI.

MACROSCOPIC
1. Brain tumour: The specimen consists of multiple pieces of pale pink and haemorrhagic tissue measuring in aggregate 15×12×7mm. Blocked in toto for frozen section (1A) and (1B).
Frozen section diagnosis: Frozen section necrotic. Smear very cellular but is probably tumour. Another frozen section is being performed to exclude an infarct.
Paraffin section blocking details: (1A) and (1B) frozen section remnants.

2. Parietooccipital tumour: The specimen consists of three pieces of dark red and pale pink tissue ranging in size from 10mm to 15mm in maximal diameter.
Frozen section blocking details: (2A) rep sections.
Frozen section diagnosis: Glioblastoma multiforme.
Paraffin section blocking details: Frozen section remnant (2A); remainder of specimen wrapped and blocked in toto (2B).

3. Parietal/occipital tumour: The specimen consists of pieces of pink, haemorrhagic tissue measuring in aggregate 27×32×15mm.
Blocking details: Specimen wrapped and blocked in toto (3A) to (3C).

MICROSCOPIC
1-3. Parietooccipital tumour: The paraffin sections confirm the frozen section diagnosis of glioblastoma multiforme. There is brain tissue which is infiltrated and replaced by a cellular astrocytic tumour. There is florid endothelial proliferation along with large tracts of necrosis. In areas there is pseudopalisading around the necrotic regions. Mitoses are numerous and the tumour cells are atypical. The features are those of a glioblastoma multiforme.

SUMMARY
1-3. Parieto-occipital tumour: Glioblastoma (WHO grade IV).
