# Supplementary material for: A reference collection of patient-derived cell line and xenograft models of proneural, classical and mesenchymal glioblastoma
Source: Sci Rep. 2019 Mar 20;9:4902. doi: 10.1038/s41598-019-41277-z (PMC6427001; doi:10.1038/s41598-019-41277-z)
Supplement: Supplementary file 7 — Dataset 5 [file 41598_2019_41277_MOESM7_ESM.pdf]

A reference collection of patient-derived cell line and xenograft models of proneural, classical and mesenchymal glioblastoma

Brett W Stringer[1,*], Bryan W Day[1,*], Rochelle C J D'Souza[1], Paul R Jamieson[1], Kathleen S Ensbey[1], Zara C Bruce[1], Yi Chieh Lim[1], Kate Goasdoué[1], Carolin Offenhäuser[1], Seçkin Akgül[1], Suzanne Allan[1,2], Thomas Robertson[2], Peter Lucas[2], Gert Tollesson[2], Scott Campbell[2], Craig Winter[2], Hongdo Do[3], Alexander Dobrovic[3], Po-Ling Inglis[1,2], Rosalind L Jeffree[2,4], Terrance G Johns[5,+], Andrew W Boyd[1,4,+]

[1]QIMR Berghofer Medical Research Institute, Brisbane, Australia.
[2]Royal Brisbane and Women's Hospital, Brisbane, Australia.
[3]Olivia Newton-John Cancer and Wellness Centre, Melbourne, Australia.
[4]The University of Queensland, Brisbane, Australia.
[5]Hudson Institute of Medical Research, Clayton, Victoria, Australia.

[*]Joint first authors.
[+]Joint senior authors.

Correspondence should be addressed to B.W.S. (brett.w.stringer@gmail.com).

CD133

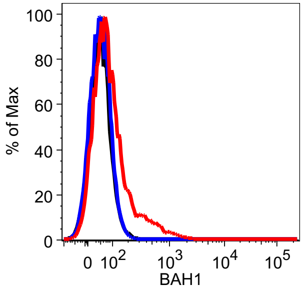

Sample	Mean:PE-A
Unfixed_CD133.fcs	127
Unfixed_IgG1.fcs	*46.8*
Unfixed_Secondary Only.fcs	*47.1*

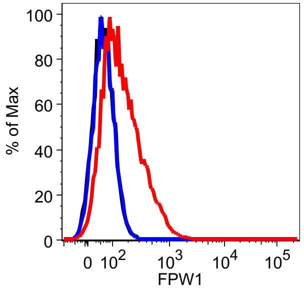

Sample	Mean:PE-A
Unfixed_CD133.fcs	201
Unfixed_IgG1.fcs	*62.9*
Unfixed_Secondary Only.fcs	*61.4*

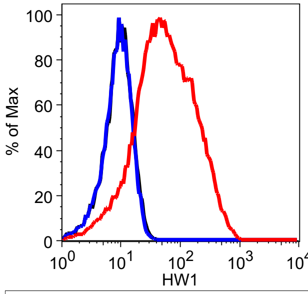

Sample	Mean:PE-A
Unfixed Cells_CD133.fcs	99.2
Unfixed Cells_IgG1.fcs	*9.75*
Unfixed Cells_Secondary Only.fcs	*9.96*

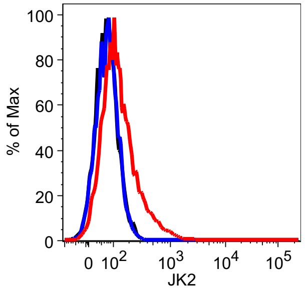

Sample	Mean:PE-A
Unfixed_CD133.fcs	169
Unfixed_IgG1.fcs	*73.9*
Unfixed_Secondary Only.fcs	*73.1*

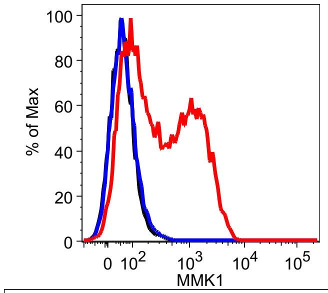

Sample	Mean:PE-A
MMK1_CD133.fcs	697
MMK1_IgG1.fcs	*64*
MMK1_Secondary Only.fcs	*58.2*

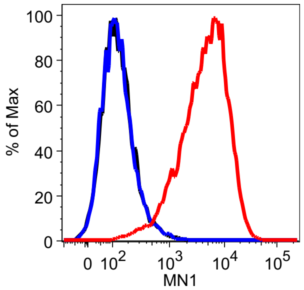

Sample	Mean:PE-A
Unfixed_CD133.fcs	6439
Unfixed_IgG1.fcs	*153*
Unfixed_Secondary only.fcs	*154*

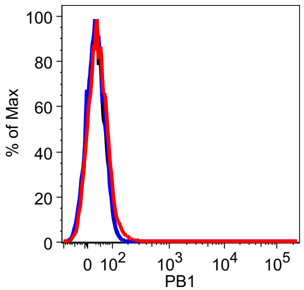

Sample	Mean:PE-A
Unfixed_CD133.fcs	38.4
Unfixed_IgG1.fcs	*27.9*
Unfixed_Secondary Only.fcs	*29*

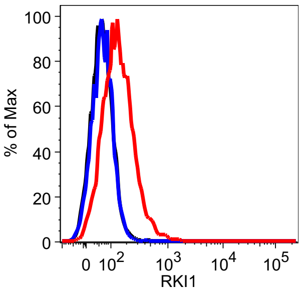

Sample	Mean:PE-A
Unfixed_CD133.fcs	167
Unfixed_IgG1.fcs	*63.8*
Unfixed_Secondary only.fcs	*63.1*

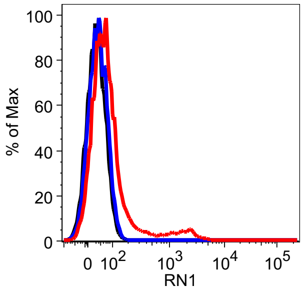

SampleName	Mean:PE-A
Unfixed_CD133.fcs	174
Unfixed_IgG1.fcs	*36.7*
Unfixed_Secondary only.fcs	*30.7*

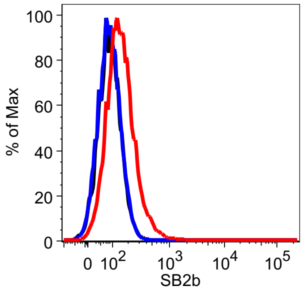

Sample	Mean:PE-A
Unfixed_CD133.fcs	168
Unfixed_IgG1.fcs	*89.5*
Unfixed_Secondary only.fcs	*90.5*

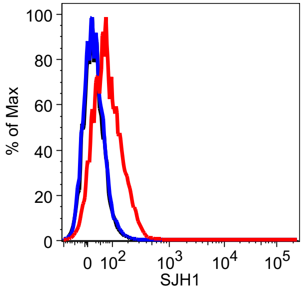

Sample	Mean:PE-A
Unfixed_CD133.fcs	85.6
Unfixed_IgG1.fcs	*22.3*
Unfixed_Secondary Only.fcs	*21.8*

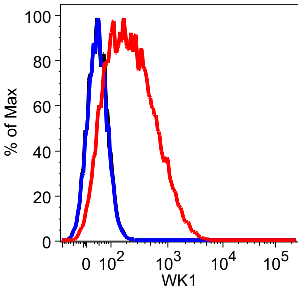

Sample	Mean:PE-A
Unfixed_CD133.fcs	360
Unfixed_IgG1.fcs	*42.8*
Unfixed_Secondary only.fcs	*43.1*

CD15

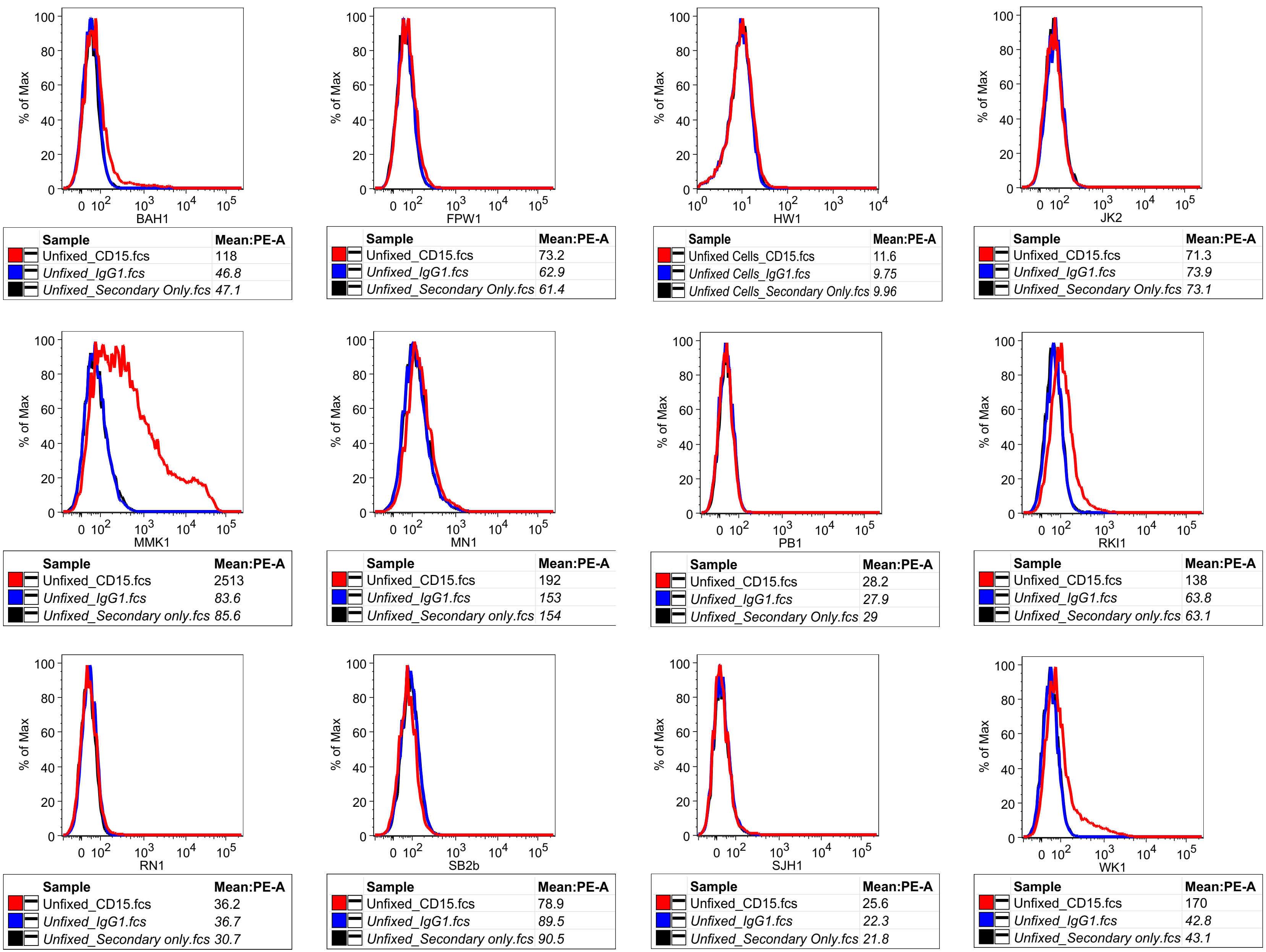

% of Max
BAH1
Sample | Mean:PE-A
Unfixed_CD15.fcs 118
Unfixed_IgG1.fcs 46.8
Unfixed_Secondary Only.fcs 47.1
FPW1
Unfixed_CD15.fcs 73.2
Unfixed_IgG1.fcs 62.9
Unfixed_Secondary Only.fcs 61.4
HW1
Unfixed Cells_CD15.fcs 11.6
Unfixed Cells_IgG1.fcs 9.75
Unfixed Cells_Secondary Only.fcs 9.96
JK2
Unfixed_CD15.fcs 71.3
Unfixed_IgG1.fcs 73.9
Unfixed_Secondary Only.fcs 73.1
MMK1
Unfixed_CD15.fcs 2513
Unfixed_IgG1.fcs 83.6
Unfixed_Secondary only.fcs 85.6
MN1
Unfixed_CD15.fcs 192
Unfixed_IgG1.fcs 153
Unfixed_Secondary only.fcs 154
PB1
Unfixed_CD15.fcs 28.2
Unfixed_IgG1.fcs 27.9
Unfixed_Secondary Only.fcs 29
RKI1
Unfixed_CD15.fcs 138
Unfixed_IgG1.fcs 63.8
Unfixed_Secondary only.fcs 63.1
RN1
Unfixed_CD15.fcs 36.2
Unfixed_IgG1.fcs 36.7
Unfixed_Secondary only.fcs 30.7
SB2b
Unfixed_CD15.fcs 78.9
Unfixed_IgG1.fcs 89.5
Unfixed_Secondary only.fcs 90.5
SJH1
Unfixed_CD15.fcs 25.6
Unfixed_IgG1.fcs 22.3
Unfixed_Secondary Only.fcs 21.8
WK1
Unfixed_CD15.fcs 170
Unfixed_IgG1.fcs 42.8
Unfixed_Secondary only.fcs 43.1

CD44

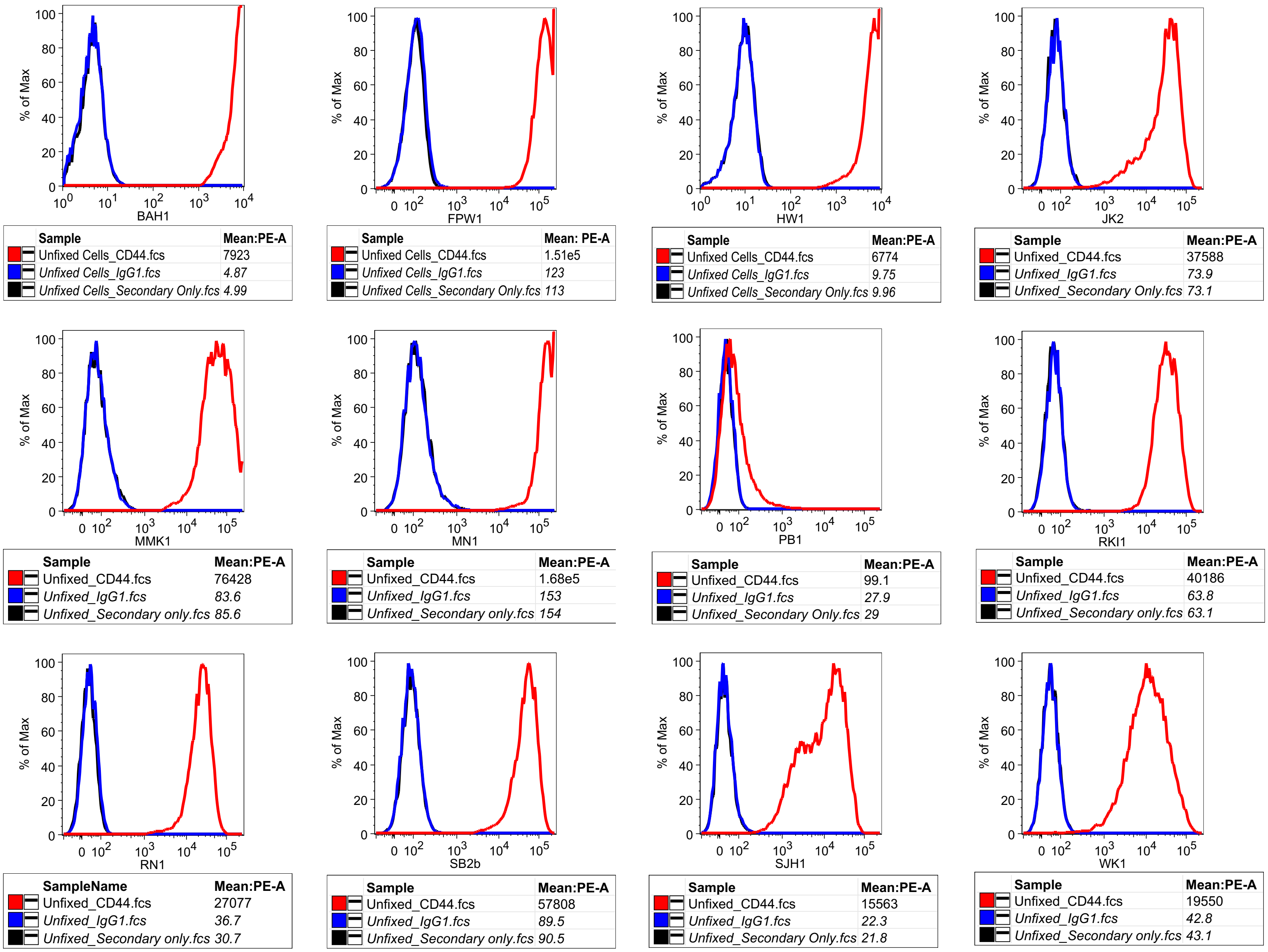
% of Max
BAH1
Sample | Mean:PE-A
Unfixed Cells_CD44.fcs 7923
Unfixed Cells_IgG1.fcs 4.87
Unfixed Cells_Secondary Only.fcs 4.99
FPW1
Sample | Mean: PE-A
Unfixed Cells_CD44.fcs 1.51e5
Unfixed Cells_IgG1.fcs 123
Unfixed Cells_Secondary Only.fcs 113
HW1
Sample | Mean:PE-A
Unfixed Cells_CD44.fcs 6774
Unfixed Cells_IgG1.fcs 9.75
Unfixed Cells_Secondary Only.fcs 9.96
JK2
Sample | Mean:PE-A
Unfixed_CD44.fcs 37588
Unfixed_IgG1.fcs 73.9
Unfixed_Secondary Only.fcs 73.1
MMK1
Sample | Mean:PE-A
Unfixed_CD44.fcs 76428
Unfixed_IgG1.fcs 83.6
Unfixed_Secondary only.fcs 85.6
MN1
Sample | Mean:PE-A
Unfixed_CD44.fcs 1.68e5
Unfixed_IgG1.fcs 153
Unfixed_Secondary only.fcs 154
PB1
Sample | Mean:PE-A
Unfixed_CD44.fcs 99.1
Unfixed_IgG1.fcs 27.9
Unfixed_Secondary Only.fcs 29
RKI1
Sample | Mean:PE-A
Unfixed_CD44.fcs 40186
Unfixed_IgG1.fcs 63.8
Unfixed_Secondary only.fcs 63.1
RN1
SampleName | Mean:PE-A
Unfixed_CD44.fcs 27077
Unfixed_IgG1.fcs 36.7
Unfixed_Secondary only.fcs 30.7
SB2b
Sample | Mean:PE-A
Unfixed_CD44.fcs 57808
Unfixed_IgG1.fcs 89.5
Unfixed_Secondary only.fcs 90.5
SJH1
Sample | Mean:PE-A
Unfixed_CD44.fcs 15563
Unfixed_IgG1.fcs 22.3
Unfixed_Secondary Only.fcs 21.8
WK1
Sample | Mean:PE-A
Unfixed_CD44.fcs 19550
Unfixed_IgG1.fcs 42.8
Unfixed_Secondary only.fcs 43.1

CD49f

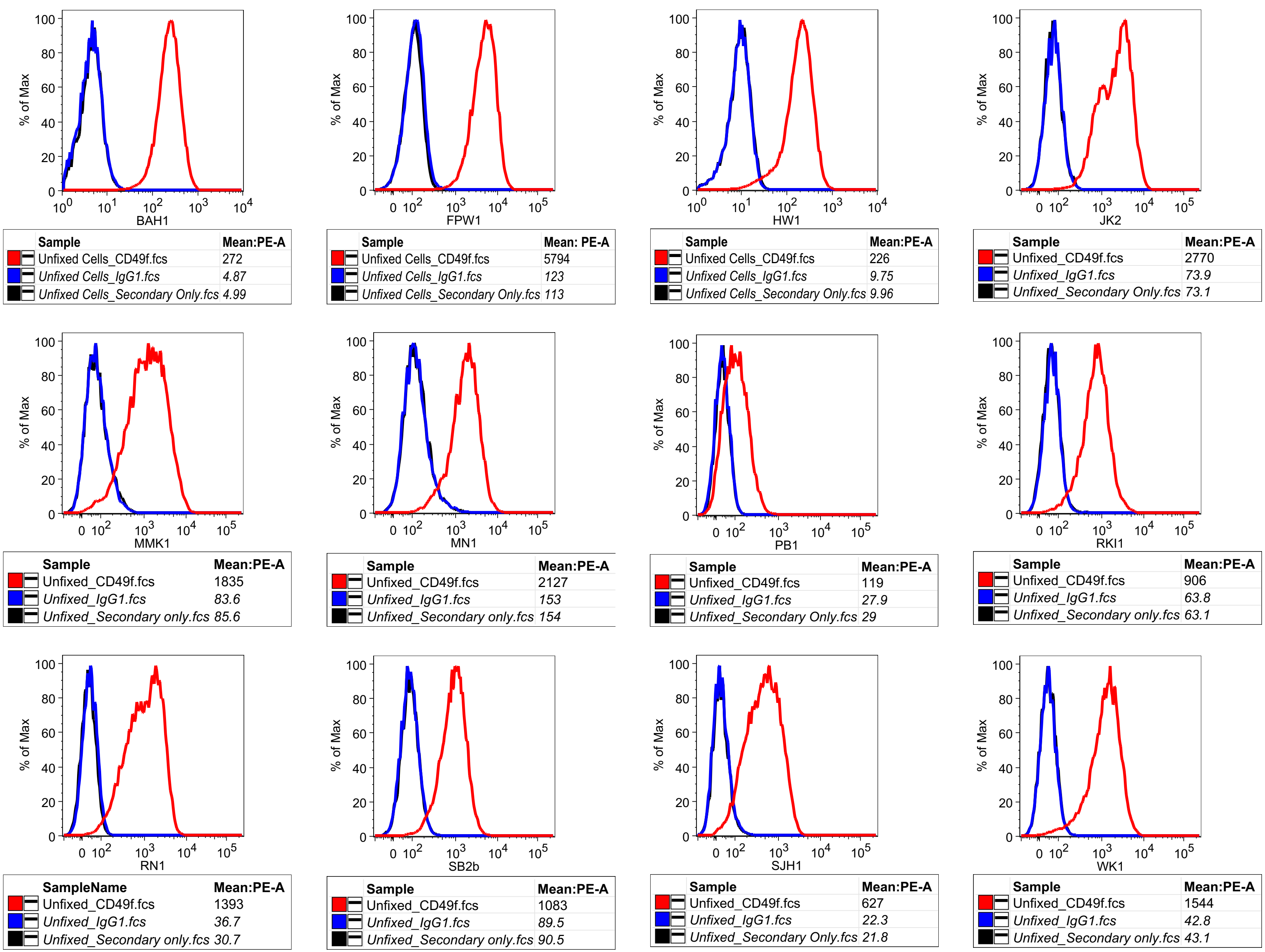

% of Max
BAH1
Sample Mean:PE-A
Unfixed Cells_CD49f.fcs 272
Unfixed Cells_IgG1.fcs 4.87
Unfixed Cells_Secondary Only.fcs 4.99
FPW1
Sample Mean: PE-A
Unfixed Cells_CD49f.fcs 5794
Unfixed Cells_IgG1.fcs 123
Unfixed Cells_Secondary Only.fcs 113
HW1
Sample Mean:PE-A
Unfixed Cells_CD49f.fcs 226
Unfixed Cells_IgG1.fcs 9.75
Unfixed Cells_Secondary Only.fcs 9.96
JK2
Sample Mean:PE-A
Unfixed_CD49f.fcs 2770
Unfixed_IgG1.fcs 73.9
Unfixed_Secondary Only.fcs 73.1
MMK1
Sample Mean:PE-A
Unfixed_CD49f.fcs 1835
Unfixed_IgG1.fcs 83.6
Unfixed_Secondary only.fcs 85.6
MN1
Sample Mean:PE-A
Unfixed_CD49f.fcs 2127
Unfixed_IgG1.fcs 153
Unfixed_Secondary only.fcs 154
PB1
Sample Mean:PE-A
Unfixed_CD49f.fcs 119
Unfixed_IgG1.fcs 27.9
Unfixed_Secondary Only.fcs 29
RKI1
Sample Mean:PE-A
Unfixed_CD49f.fcs 906
Unfixed_IgG1.fcs 63.8
Unfixed_Secondary only.fcs 63.1
RN1
SampleName Mean:PE-A
Unfixed_CD49f.fcs 1393
Unfixed_IgG1.fcs 36.7
Unfixed_Secondary only.fcs 30.7
SB2b
Sample Mean:PE-A
Unfixed_CD49f.fcs 1083
Unfixed_IgG1.fcs 89.5
Unfixed_Secondary only.fcs 90.5
SJH1
Sample Mean:PE-A
Unfixed_CD49f.fcs 627
Unfixed_IgG1.fcs 22.3
Unfixed_Secondary Only.fcs 21.8
WK1
Sample Mean:PE-A
Unfixed_CD49f.fcs 1544
Unfixed_IgG1.fcs 42.8
Unfixed_Secondary only.fcs 43.1

EGFR

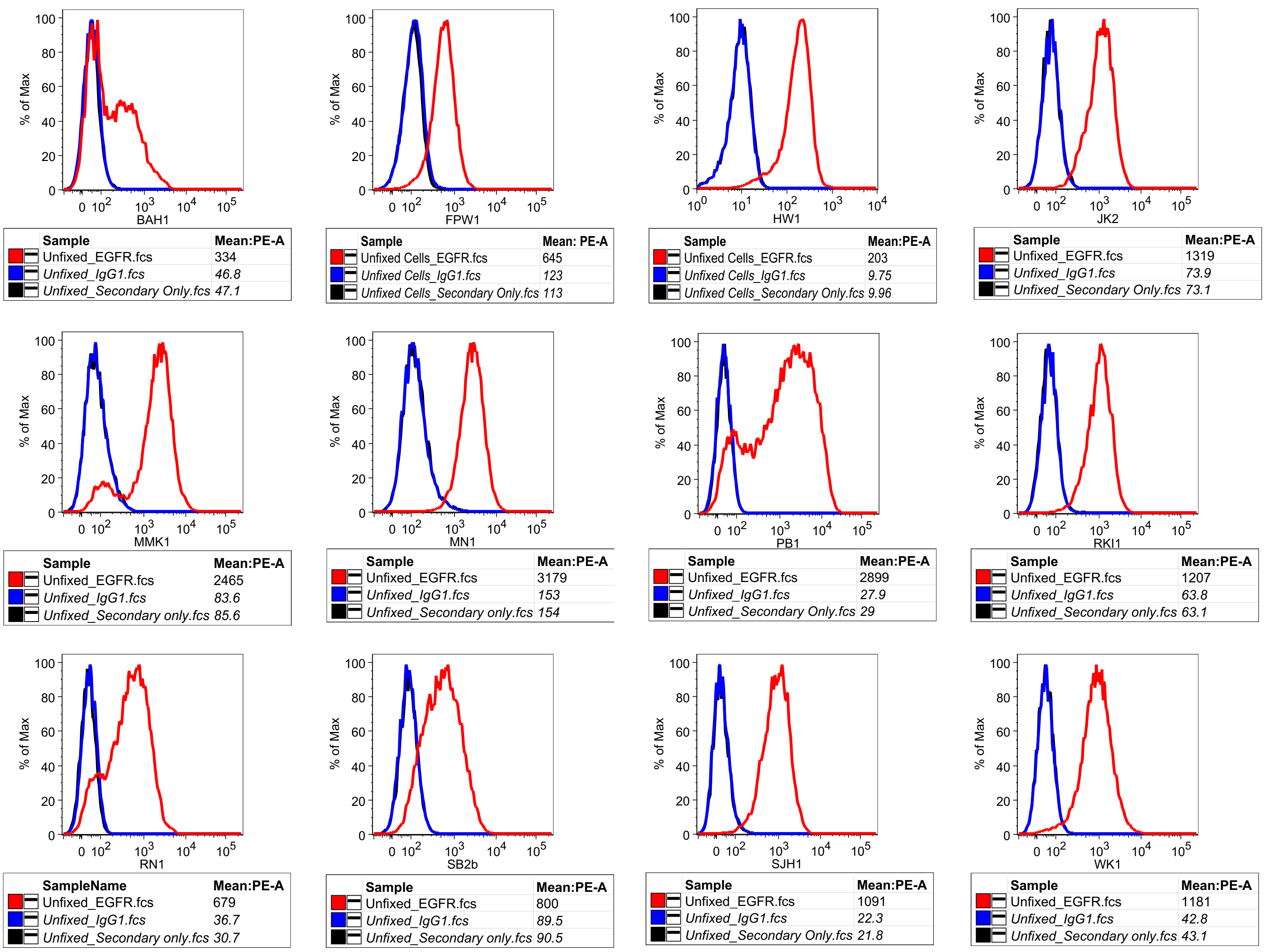

% of Max
BAH1
Sample | Mean:PE-A
Unfixed_EGFR.fcs 334
Unfixed_IgG1.fcs 46.8
Unfixed_Secondary Only.fcs 47.1
FPW1
Sample | Mean: PE-A
Unfixed Cells_EGFR.fcs 645
Unfixed Cells_IgG1.fcs 123
Unfixed Cells_Secondary Only.fcs 113
HW1
Sample | Mean:PE-A
Unfixed Cells_EGFR.fcs 203
Unfixed Cells_IgG1.fcs 9.75
Unfixed Cells_Secondary Only.fcs 9.96
JK2
Sample | Mean:PE-A
Unfixed_EGFR.fcs 1319
Unfixed_IgG1.fcs 73.9
Unfixed_Secondary Only.fcs 73.1
MMK1
Sample | Mean:PE-A
Unfixed_EGFR.fcs 2465
Unfixed_IgG1.fcs 83.6
Unfixed_Secondary only.fcs 85.6
MN1
Sample | Mean:PE-A
Unfixed_EGFR.fcs 3179
Unfixed_IgG1.fcs 153
Unfixed_Secondary only.fcs 154
PB1
Sample | Mean:PE-A
Unfixed_EGFR.fcs 2899
Unfixed_IgG1.fcs 27.9
Unfixed_Secondary Only.fcs 29
RKI1
Sample | Mean:PE-A
Unfixed_EGFR.fcs 1207
Unfixed_IgG1.fcs 63.8
Unfixed_Secondary only.fcs 63.1
RN1
SampleName | Mean:PE-A
Unfixed_EGFR.fcs 679
Unfixed_IgG1.fcs 36.7
Unfixed_Secondary only.fcs 30.7
SB2b
Sample | Mean:PE-A
Unfixed_EGFR.fcs 800
Unfixed_IgG1.fcs 89.5
Unfixed_Secondary only.fcs 90.5
SJH1
Sample | Mean:PE-A
Unfixed_EGFR.fcs 1091
Unfixed_IgG1.fcs 22.3
Unfixed_Secondary Only.fcs 21.8
WK1
Sample | Mean:PE-A
Unfixed_EGFR.fcs 1181
Unfixed_IgG1.fcs 42.8
Unfixed_Secondary only.fcs 43.1

EGFRvIII

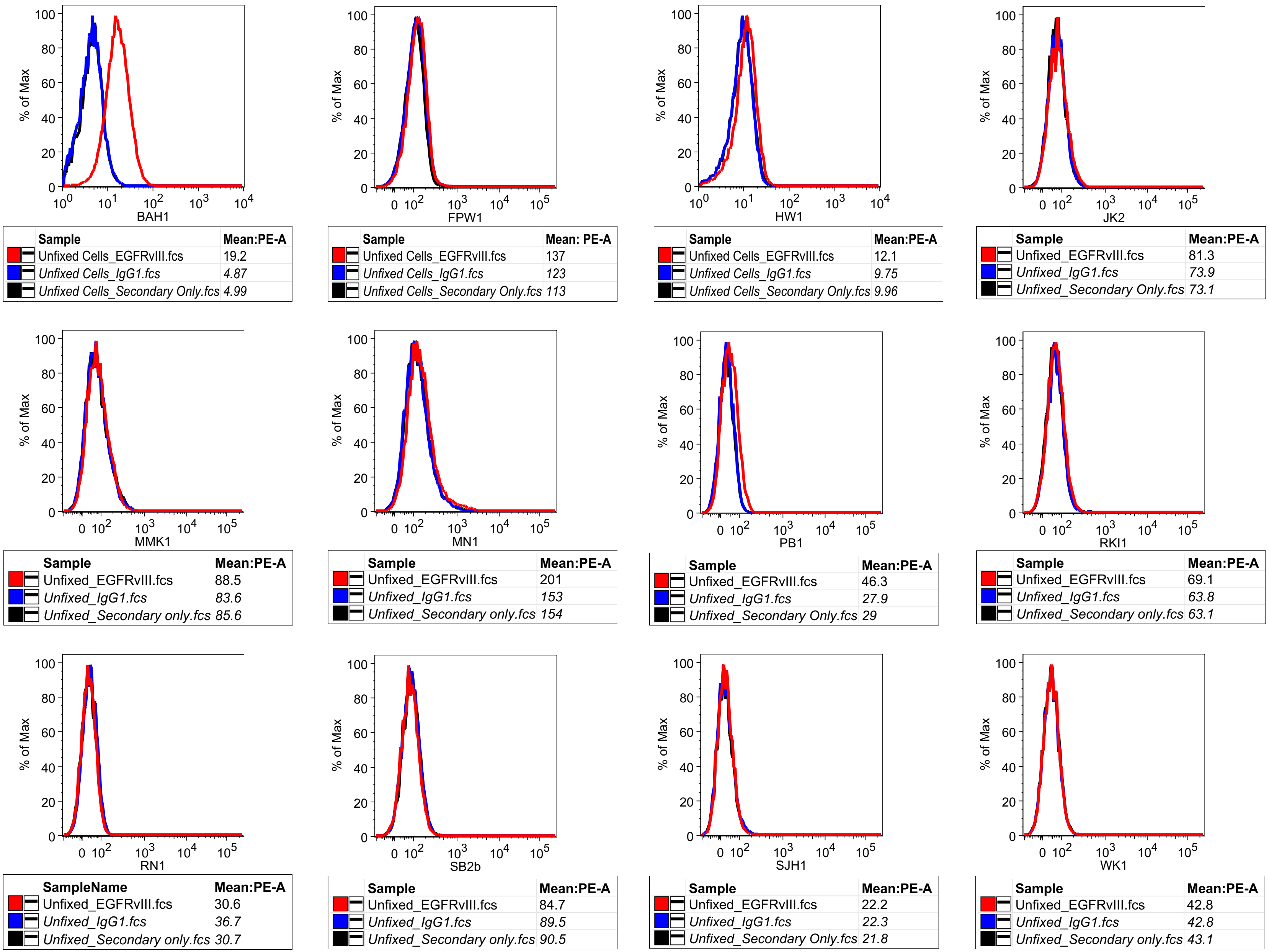
% of Max
BAH1
Sample | Mean:PE-A
Unfixed Cells_EGFRvIII.fcs 19.2
Unfixed Cells_IgG1.fcs 4.87
Unfixed Cells_Secondary Only.fcs 4.99
FPW1
Sample | Mean: PE-A
Unfixed Cells_EGFRvIII.fcs 137
Unfixed Cells_IgG1.fcs 123
Unfixed Cells_Secondary Only.fcs 113
HW1
Sample | Mean:PE-A
Unfixed Cells_EGFRvIII.fcs 12.1
Unfixed Cells_IgG1.fcs 9.75
Unfixed Cells_Secondary Only.fcs 9.96
JK2
Sample | Mean:PE-A
Unfixed_EGFRvIII.fcs 81.3
Unfixed_IgG1.fcs 73.9
Unfixed_Secondary Only.fcs 73.1
MMK1
Sample | Mean:PE-A
Unfixed_EGFRvIII.fcs 88.5
Unfixed_IgG1.fcs 83.6
Unfixed_Secondary only.fcs 85.6
MN1
Sample | Mean:PE-A
Unfixed_EGFRvIII.fcs 201
Unfixed_IgG1.fcs 153
Unfixed_Secondary only.fcs 154
PB1
Sample | Mean:PE-A
Unfixed_EGFRvIII.fcs 46.3
Unfixed_IgG1.fcs 27.9
Unfixed_Secondary Only.fcs 29
RKI1
Sample | Mean:PE-A
Unfixed_EGFRvIII.fcs 69.1
Unfixed_IgG1.fcs 63.8
Unfixed_Secondary only.fcs 63.1
RN1
SampleName | Mean:PE-A
Unfixed_EGFRvIII.fcs 30.6
Unfixed_IgG1.fcs 36.7
Unfixed_Secondary only.fcs 30.7
SB2b
Sample | Mean:PE-A
Unfixed_EGFRvIII.fcs 84.7
Unfixed_IgG1.fcs 89.5
Unfixed_Secondary only.fcs 90.5
SJH1
Sample | Mean:PE-A
Unfixed_EGFRvIII.fcs 22.2
Unfixed_IgG1.fcs 22.3
Unfixed_Secondary Only.fcs 21.8
WK1
Sample | Mean:PE-A
Unfixed_EGFRvIII.fcs 42.8
Unfixed_IgG1.fcs 42.8
Unfixed_Secondary only.fcs 43.1

PDGFRa

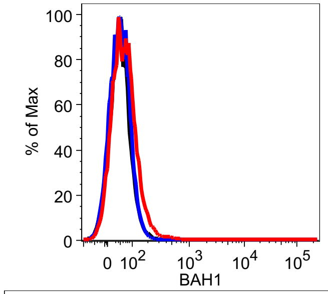

	Sample	Mean:PE-A
Red	Unfixed_PDGFRa.fcs	69.3
Blue	*Unfixed_IgG1.fcs*	*46.8*
Black	*Unfixed_Secondary Only.fcs*	*47.1*

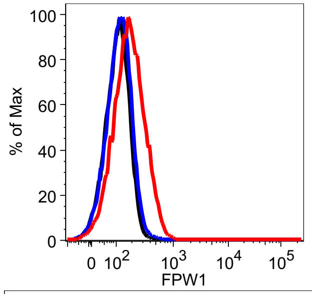

	Sample	Mean: PE-A
Red	Unfixed Cells_PDGFRa.fcs	203
Blue	*Unfixed Cells_IgG1.fcs*	*123*
Black	*Unfixed Cells_Secondary Only.fcs*	*113*

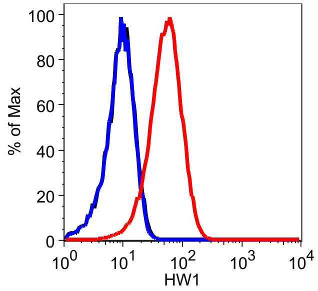

	Sample	Mean:PE-A
Red	Unfixed Cells_PDGFRa.fcs	61.2
Blue	*Unfixed Cells_IgG1.fcs*	*9.75*
Black	*Unfixed Cells_Secondary Only.fcs*	*9.96*

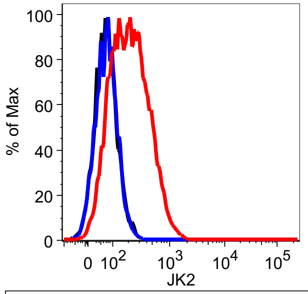

	Sample	Mean:PE-A
Red	Unfixed_PDGFRa.fcs	262
Blue	*Unfixed_IgG1.fcs*	*73.9*
Black	*Unfixed_Secondary Only.fcs*	*73.1*

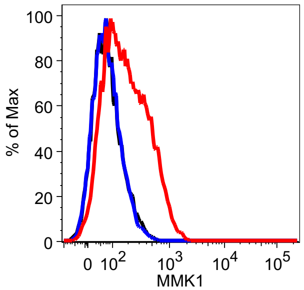

	Sample	Mean:PE-A
Red	Unfixed_PDGFRa.fcs	250
Blue	*Unfixed_IgG1.fcs*	*83.6*
Black	*Unfixed_Secondary only.fcs*	*85.6*

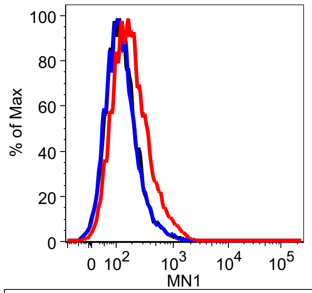

	Sample	Mean:PE-A
Red	Unfixed_PDGFRa.fcs	243
Blue	*Unfixed_IgG1.fcs*	*153*
Black	*Unfixed_Secondary only.fcs*	*154*

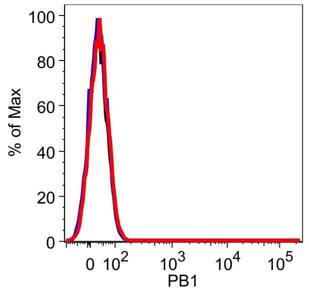

	Sample	Mean:PE-A
Red	Unfixed_PDGFRa.fcs	31.3
Blue	*Unfixed_IgG1.fcs*	*27.9*
Black	*Unfixed_Secondary Only.fcs*	*29*

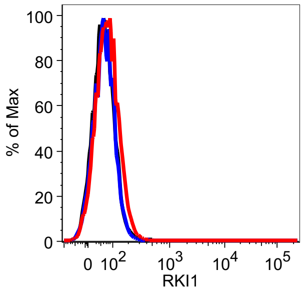

	Sample	Mean:PE-A
Red	Unfixed_PDGFRa_01.fcs	79.2
Blue	*Unfixed_IgG1.fcs*	*63.8*
Black	*Unfixed_Secondary only.fcs*	*63.1*

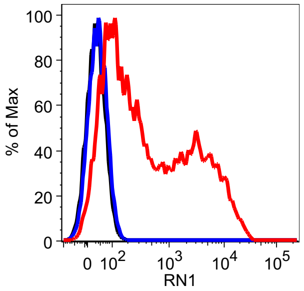

	SampleName	Mean:PE-A
Red	Unfixed_PDGFRa.fcs	2038
Blue	*Unfixed_IgG1.fcs*	*36.7*
Black	*Unfixed_Secondary only.fcs*	*30.7*

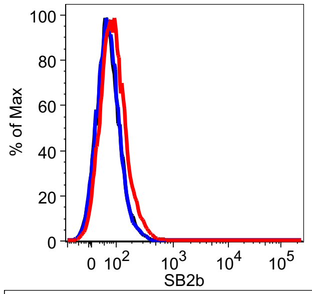

	Sample	Mean:PE-A
Red	SB2b_PDGFRa.fcs	95.3
Blue	*SB2b_IgG1.fcs*	*68.2*
Black	*SB2b_Secondary Only.fcs*	*68.8*

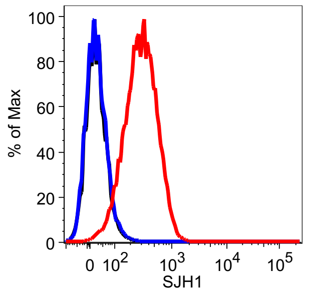

	Sample	Mean:PE-A
Red	Unfixed_PDGFRa.fcs	351
Blue	*Unfixed_IgG1.fcs*	*22.3*
Black	*Unfixed_Secondary Only.fcs*	*21.8*

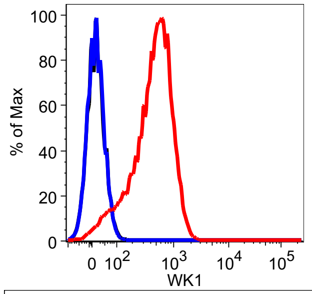

	Sample	Mean:PE-A
Red	WK1_PDGFRa.fcs	538
Blue	*WK1_IgG1.fcs*	*11.9*
Black	*WK1_Secondary Only.fcs*	*12.2*

EphA2

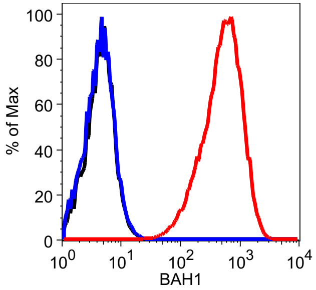

Sample	Mean:PE-A
Unfixed Cells_EphA2.fcs	647
Unfixed Cells_IgG1.fcs	*4.87*
Unfixed Cells_Secondary Only.fcs	*4.99*

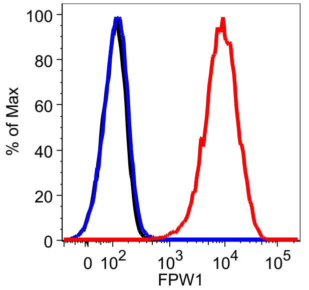

Sample	Mean: PE-A
Unfixed Cells_EphA2.fcs	10976
Unfixed Cells_IgG1.fcs	*123*
Unfixed Cells_Secondary Only.fcs	*113*

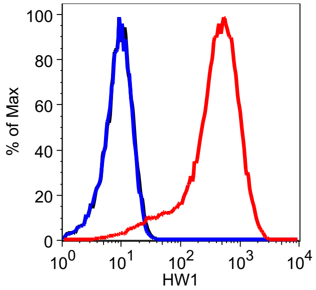

Sample	Mean:PE-A
Unfixed Cells_EphA2.fcs	530
Unfixed Cells_IgG1.fcs	*9.75*
Unfixed Cells_Secondary Only.fcs	*9.96*

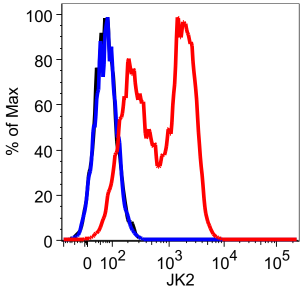

Sample	Mean:PE-A
Unfixed_EphA2.fcs	1188
Unfixed_IgG1.fcs	*73.9*
Unfixed_Secondary Only.fcs	*73.1*

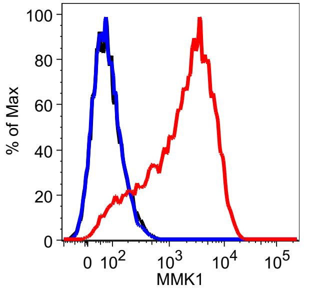

Sample	Mean:PE-A
Unfixed_EphA2.fcs	3091
Unfixed_IgG1.fcs	*83.6*
Unfixed_Secondary only.fcs	*85.6*

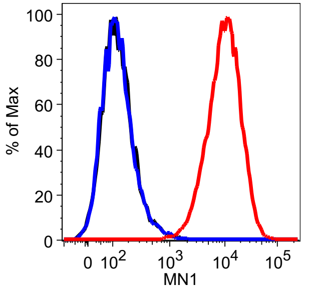

Sample	Mean:PE-A
Unfixed_EphA2.fcs	12769
Unfixed_IgG1.fcs	*153*
Unfixed_Secondary only.fcs	*154*

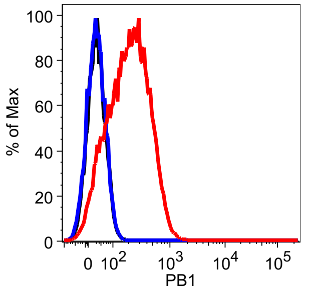

Sample	Mean:PE-A
Unfixed_EphA2.fcs	253
Unfixed_IgG1.fcs	*27.9*
Unfixed_Secondary Only.fcs	*29*

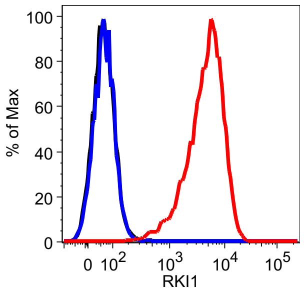

Sample	Mean:PE-A
Unfixed_EphA2.fcs	5697
Unfixed_IgG1.fcs	*63.8*
Unfixed_Secondary only.fcs	*63.1*

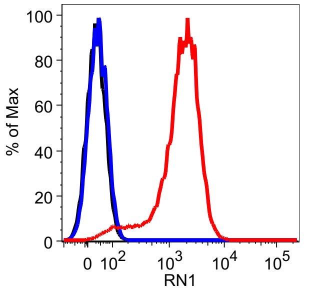

SampleName	Mean:PE-A
Unfixed_EphA2.fcs	2029
Unfixed_IgG1.fcs	*36.7*
Unfixed_Secondary only.fcs	*30.7*

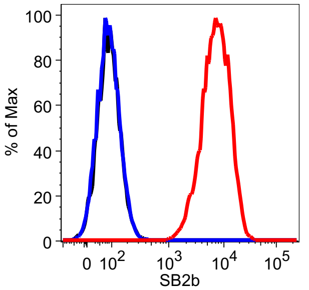

Sample	Mean:PE-A
Unfixed_EphA2.fcs	8660
Unfixed_IgG1.fcs	*89.5*
Unfixed_Secondary only.fcs	*90.5*

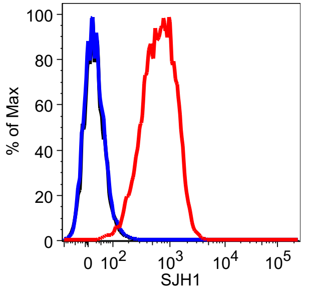

Sample	Mean:PE-A
Unfixed_EphA2.fcs	786
Unfixed_IgG1.fcs	*22.3*
Unfixed_Secondary Only.fcs	*21.8*

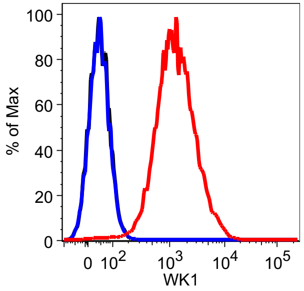

Sample	Mean:PE-A
Unfixed_EphA2.fcs	1798
Unfixed_IgG1.fcs	*42.8*
Unfixed_Secondary only.fcs	*43.1*

EphA3

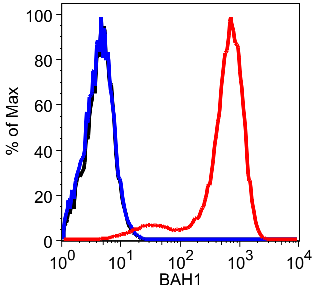

Sample	Mean:PE-A
Unfixed Cells_EphA3.fcs	695
Unfixed Cells_IgG1.fcs	*4.87*
Unfixed Cells_Secondary Only.fcs	*4.99*

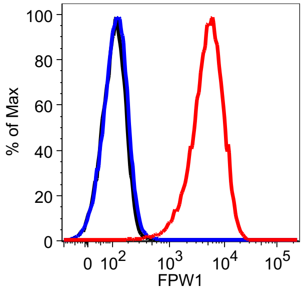

Sample	Mean: PE-A
Unfixed Cells_EphA3.fcs	6002
Unfixed Cells_IgG1.fcs	*123*
Unfixed Cells_Secondary Only.fcs	*113*

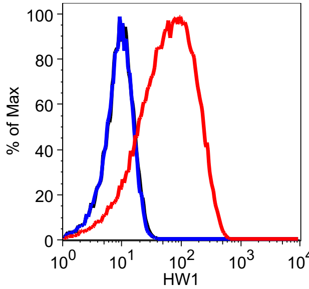

Sample	Mean:PE-A
Unfixed Cells_EphA3.fcs	91.5
Unfixed Cells_IgG1.fcs	*9.75*
Unfixed Cells_Secondary Only.fcs	*9.96*

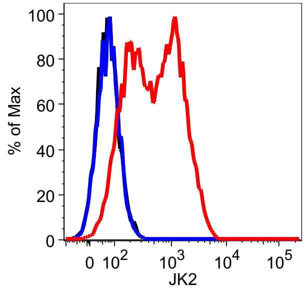

Sample	Mean:PE-A
Unfixed_EphA3.fcs	816
Unfixed_IgG1.fcs	*73.9*
Unfixed_Secondary Only.fcs	*73.1*

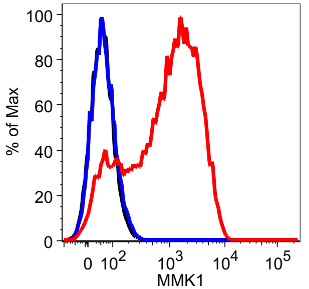

Sample	Mean:PE-A
MMK1_EphA3.fcs	1596
MMK1_IgG1.fcs	61.4
MMK1_Secondary Only.fcs	56.4

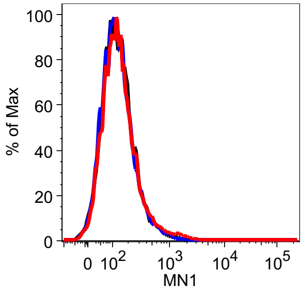

Sample	Mean:PE-A
Unfixed_EphA3.fcs	182
Unfixed_IgG1.fcs	*153*
Unfixed_Secondary only.fcs	*154*

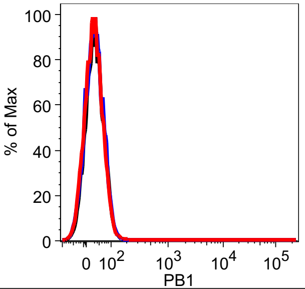

Sample	Mean:PE-A
Unfixed_EphA3.fcs	27
Unfixed_IgG1.fcs	*27.9*
Unfixed_Secondary Only.fcs	*29*

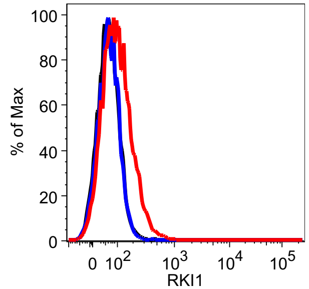

Sample	Mean:PE-A
Unfixed_EphA3.fcs	114
Unfixed_IgG1.fcs	*63.8*
Unfixed_Secondary only.fcs	*63.1*

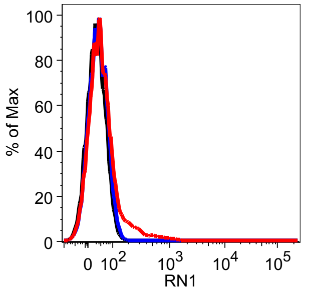

SampleName	Mean:PE-A
Unfixed_EphA3.fcs	75.2
Unfixed_IgG1.fcs	*36.7*
Unfixed_Secondary only.fcs	*30.7*

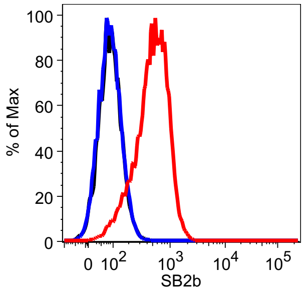

Sample	Mean:PE-A
Unfixed_EphA3.fcs	575
Unfixed_IgG1.fcs	*89.5*
Unfixed_Secondary only.fcs	*90.5*

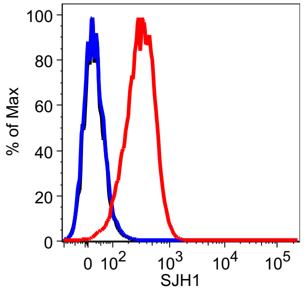

Sample	Mean:PE-A
Unfixed_EphA3.fcs	355
Unfixed_IgG1.fcs	*22.3*
Unfixed_Secondary Only.fcs	*21.8*

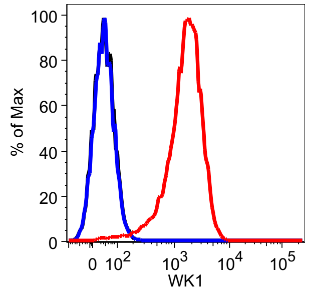

Sample	Mean:PE-A
Unfixed_EphA3.fcs	1887
Unfixed_IgG1.fcs	*42.8*
Unfixed_Secondary only.fcs	*43.1*

EphA4

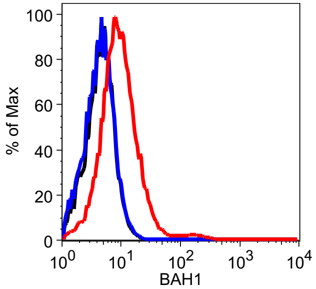

Sample	Mean:PE-A
Unfixed Cells_EphA4 (1A7).fcs	14
Unfixed Cells_IgG1.fcs	*4.87*
Unfixed Cells_Secondary Only.fcs	*4.99*

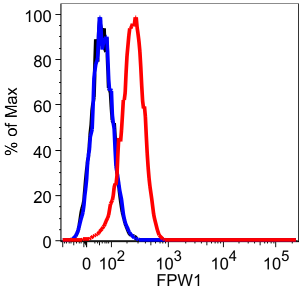

Sample	Mean:PE-A
Unfixed_EphA4 (1A7).fcs	260
Unfixed_IgG1.fcs	*62.9*
Unfixed_Secondary Only.fcs	*61.4*

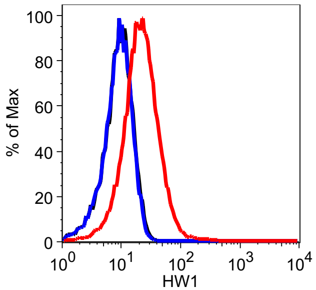

Sample	Mean:PE-A
Unfixed Cells_EphA4 (1A7).fcs	27.9
Unfixed Cells_IgG1.fcs	*9.75*
Unfixed Cells_Secondary Only.fcs	*9.96*

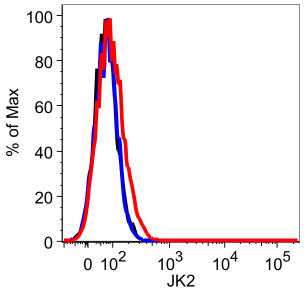

Sample	Mean:PE-A
Unfixed_EphA4 (1A7).fcs	103
Unfixed_IgG1.fcs	*73.9*
Unfixed_Secondary Only.fcs	*73.1*

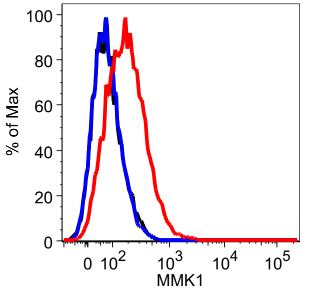

Sample	Mean:PE-A
Unfixed_EphA4 (1A7).fcs	237
Unfixed_IgG1.fcs	*83.6*
Unfixed_Secondary only.fcs	*85.6*

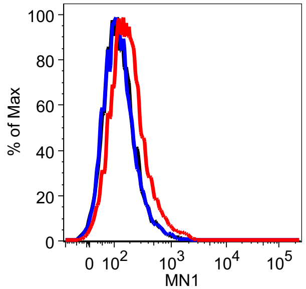

Sample	Mean:PE-A
Unfixed_EphA4 (1A7).fcs	234
Unfixed_IgG1.fcs	*153*
Unfixed_Secondary only.fcs	*154*

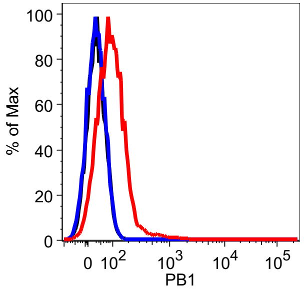

Sample	Mean:PE-A
Unfixed_EphA4 (1A7).fcs	113
Unfixed_IgG1.fcs	*27.9*
Unfixed_Secondary Only.fcs	*29*

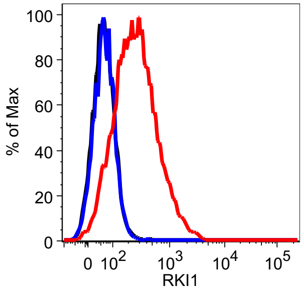

Sample	Mean:PE-A
Unfixed_EphA4 (1A7).fcs	371
Unfixed_IgG1.fcs	*63.8*
Unfixed_Secondary only.fcs	*63.1*

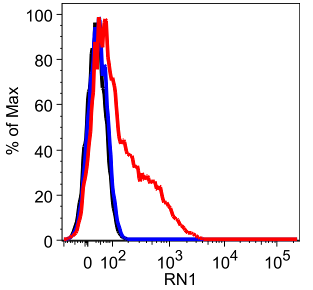

SampleName	Mean:PE-A
Unfixed_EphA4 (1A7).fcs	231
Unfixed_IgG1.fcs	*36.7*
Unfixed_Secondary only.fcs	*30.7*

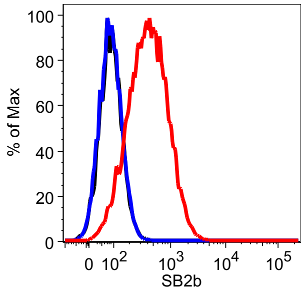

Sample	Mean:PE-A
Unfixed_EphA4 (1A7).fcs	534
Unfixed_IgG1.fcs	*89.5*
Unfixed_Secondary only.fcs	*90.5*

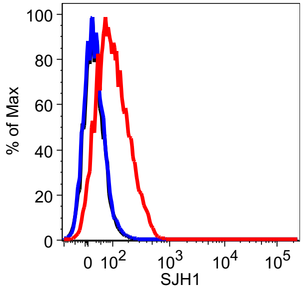

Sample	Mean:PE-A
Unfixed_EphA4 (1A7).fcs	116
Unfixed_IgG1.fcs	*22.3*
Unfixed_Secondary Only.fcs	*21.8*

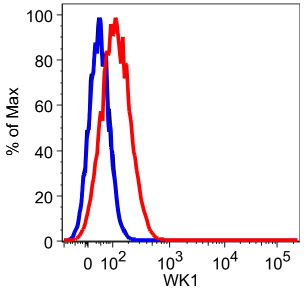

Sample	Mean:PE-A
Unfixed_EphA4 (1A7).fcs	131
Unfixed_IgG1.fcs	*42.8*
Unfixed_Secondary only.fcs	*43.1*

FGFR1

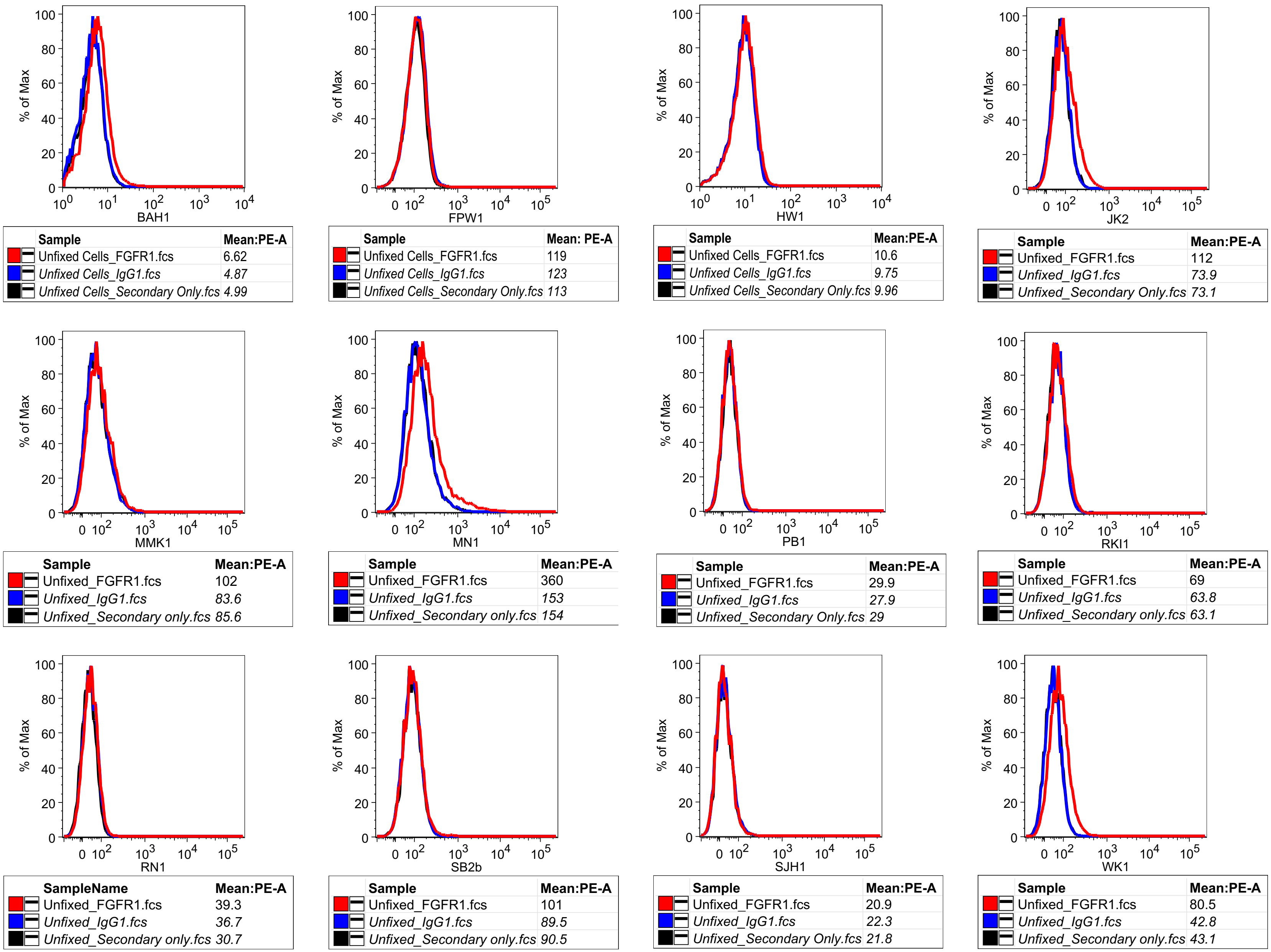

% of Max
BAH1
Sample Mean:PE-A
Unfixed Cells_FGFR1.fcs 6.62
Unfixed Cells_IgG1.fcs 4.87
Unfixed Cells_Secondary Only.fcs 4.99
FPW1
Sample Mean: PE-A
Unfixed Cells_FGFR1.fcs 119
Unfixed Cells_IgG1.fcs 123
Unfixed Cells_Secondary Only.fcs 113
HW1
Sample Mean:PE-A
Unfixed Cells_FGFR1.fcs 10.6
Unfixed Cells_IgG1.fcs 9.75
Unfixed Cells_Secondary Only.fcs 9.96
JK2
Sample Mean:PE-A
Unfixed_FGFR1.fcs 112
Unfixed_IgG1.fcs 73.9
Unfixed_Secondary Only.fcs 73.1
MMK1
Sample Mean:PE-A
Unfixed_FGFR1.fcs 102
Unfixed_IgG1.fcs 83.6
Unfixed_Secondary only.fcs 85.6
MN1
Sample Mean:PE-A
Unfixed_FGFR1.fcs 360
Unfixed_IgG1.fcs 153
Unfixed_Secondary only.fcs 154
PB1
Sample Mean:PE-A
Unfixed_FGFR1.fcs 29.9
Unfixed_IgG1.fcs 27.9
Unfixed_Secondary Only.fcs 29
RKI1
Sample Mean:PE-A
Unfixed_FGFR1.fcs 69
Unfixed_IgG1.fcs 63.8
Unfixed_Secondary only.fcs 63.1
RN1
SampleName Mean:PE-A
Unfixed_FGFR1.fcs 39.3
Unfixed_IgG1.fcs 36.7
Unfixed_Secondary only.fcs 30.7
SB2b
Sample Mean:PE-A
Unfixed_FGFR1.fcs 101
Unfixed_IgG1.fcs 89.5
Unfixed_Secondary only.fcs 90.5
SJH1
Sample Mean:PE-A
Unfixed_FGFR1.fcs 20.9
Unfixed_IgG1.fcs 22.3
Unfixed_Secondary Only.fcs 21.8
WK1
Sample Mean:PE-A
Unfixed_FGFR1.fcs 80.5
Unfixed_IgG1.fcs 42.8
Unfixed_Secondary only.fcs 43.1

FGFR2

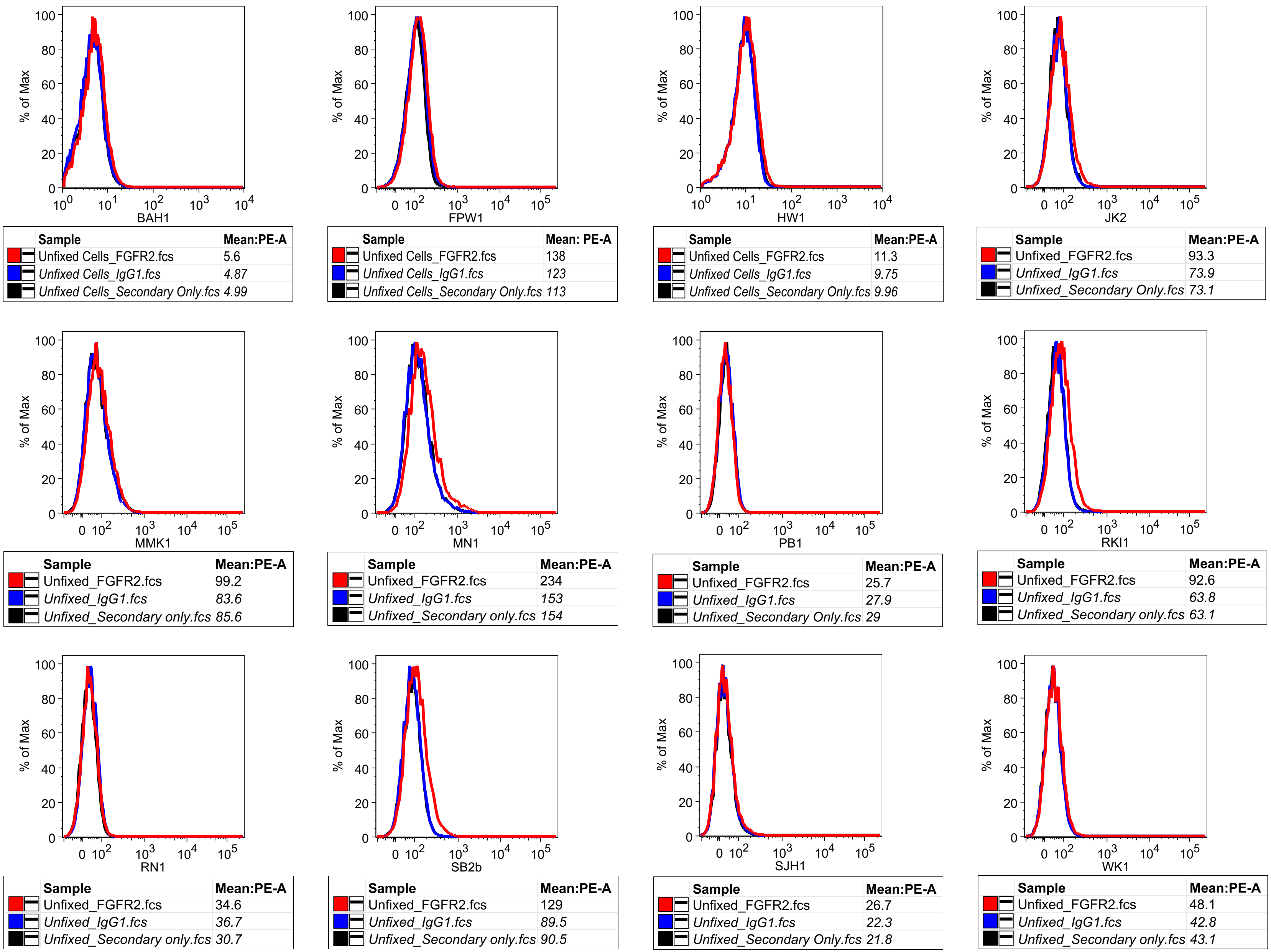
% of Max
BAH1
Sample | Mean:PE-A
Unfixed Cells_FGFR2.fcs 5.6
Unfixed Cells_IgG1.fcs 4.87
Unfixed Cells_Secondary Only.fcs 4.99
FPW1
Sample | Mean: PE-A
Unfixed Cells_FGFR2.fcs 138
Unfixed Cells_IgG1.fcs 123
Unfixed Cells_Secondary Only.fcs 113
HW1
Sample | Mean:PE-A
Unfixed Cells_FGFR2.fcs 11.3
Unfixed Cells_IgG1.fcs 9.75
Unfixed Cells_Secondary Only.fcs 9.96
JK2
Sample | Mean:PE-A
Unfixed_FGFR2.fcs 93.3
Unfixed_IgG1.fcs 73.9
Unfixed_Secondary Only.fcs 73.1
MMK1
Sample | Mean:PE-A
Unfixed_FGFR2.fcs 99.2
Unfixed_IgG1.fcs 83.6
Unfixed_Secondary only.fcs 85.6
MN1
Sample | Mean:PE-A
Unfixed_FGFR2.fcs 234
Unfixed_IgG1.fcs 153
Unfixed_Secondary only.fcs 154
PB1
Sample | Mean:PE-A
Unfixed_FGFR2.fcs 25.7
Unfixed_IgG1.fcs 27.9
Unfixed_Secondary Only.fcs 29
RKI1
Sample | Mean:PE-A
Unfixed_FGFR2.fcs 92.6
Unfixed_IgG1.fcs 63.8
Unfixed_Secondary only.fcs 63.1
RN1
Sample | Mean:PE-A
Unfixed_FGFR2.fcs 34.6
Unfixed_IgG1.fcs 36.7
Unfixed_Secondary only.fcs 30.7
SB2b
Sample | Mean:PE-A
Unfixed_FGFR2.fcs 129
Unfixed_IgG1.fcs 89.5
Unfixed_Secondary only.fcs 90.5
SJH1
Sample | Mean:PE-A
Unfixed_FGFR2.fcs 26.7
Unfixed_IgG1.fcs 22.3
Unfixed_Secondary Only.fcs 21.8
WK1
Sample | Mean:PE-A
Unfixed_FGFR2.fcs 48.1
Unfixed_IgG1.fcs 42.8
Unfixed_Secondary only.fcs 43.1

FGFR3

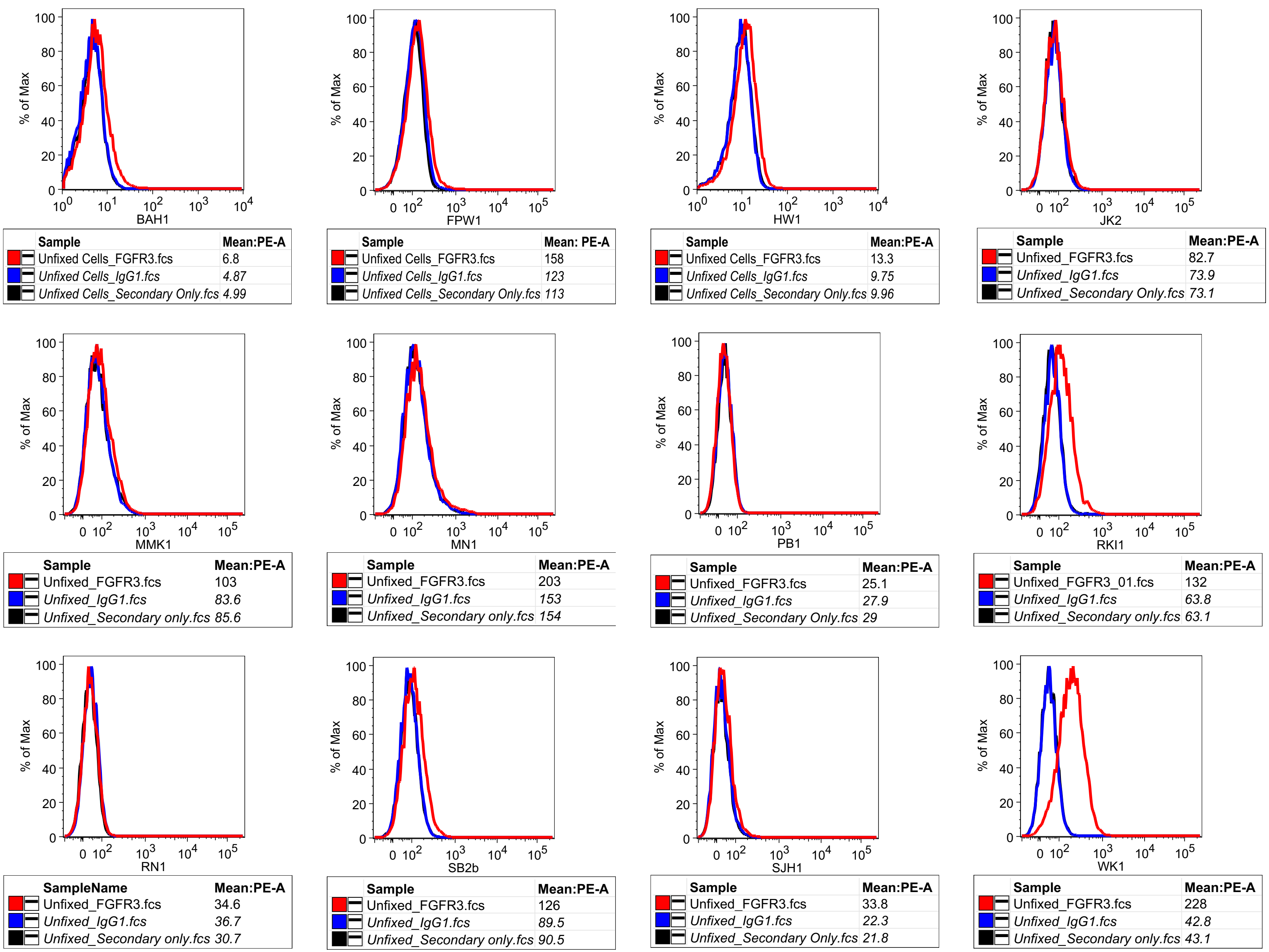
% of Max
BAH1
Sample Mean:PE-A
Unfixed Cells_FGFR3.fcs 6.8
Unfixed Cells_IgG1.fcs 4.87
Unfixed Cells_Secondary Only.fcs 4.99
FPW1
Sample Mean: PE-A
Unfixed Cells_FGFR3.fcs 158
Unfixed Cells_IgG1.fcs 123
Unfixed Cells_Secondary Only.fcs 113
HW1
Sample Mean:PE-A
Unfixed Cells_FGFR3.fcs 13.3
Unfixed Cells_IgG1.fcs 9.75
Unfixed Cells_Secondary Only.fcs 9.96
JK2
Sample Mean:PE-A
Unfixed_FGFR3.fcs 82.7
Unfixed_IgG1.fcs 73.9
Unfixed_Secondary Only.fcs 73.1
MMK1
Sample Mean:PE-A
Unfixed_FGFR3.fcs 103
Unfixed_IgG1.fcs 83.6
Unfixed_Secondary only.fcs 85.6
MN1
Sample Mean:PE-A
Unfixed_FGFR3.fcs 203
Unfixed_IgG1.fcs 153
Unfixed_Secondary only.fcs 154
PB1
Sample Mean:PE-A
Unfixed_FGFR3.fcs 25.1
Unfixed_IgG1.fcs 27.9
Unfixed_Secondary Only.fcs 29
RKI1
Sample Mean:PE-A
Unfixed_FGFR3_01.fcs 132
Unfixed_IgG1.fcs 63.8
Unfixed_Secondary only.fcs 63.1
RN1
SampleName Mean:PE-A
Unfixed_FGFR3.fcs 34.6
Unfixed_IgG1.fcs 36.7
Unfixed_Secondary only.fcs 30.7
SB2b
Sample Mean:PE-A
Unfixed_FGFR3.fcs 126
Unfixed_IgG1.fcs 89.5
Unfixed_Secondary only.fcs 90.5
SJH1
Sample Mean:PE-A
Unfixed_FGFR3.fcs 33.8
Unfixed_IgG1.fcs 22.3
Unfixed_Secondary Only.fcs 21.8
WK1
Sample Mean:PE-A
Unfixed_FGFR3.fcs 228
Unfixed_IgG1.fcs 42.8
Unfixed_Secondary only.fcs 43.1

c-MET

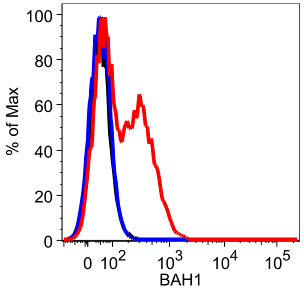

	Sample	Mean:PE-A
	Unfixed_c-MET.fcs	216
	Unfixed_IgG1.fcs	*46.8*
	Unfixed_Secondary Only.fcs	*47.1*

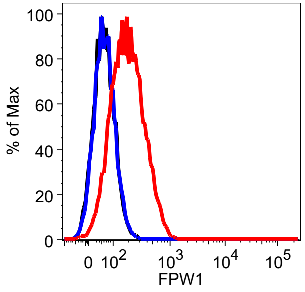

	Sample	Mean:PE-A
	Unfixed_c-MET.fcs	223
	Unfixed_IgG1.fcs	*62.9*
	Unfixed_Secondary Only.fcs	*61.4*

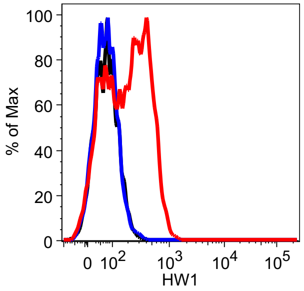

	Sample	Mean:PE-A
	Unfixed_c-MET.fcs	242
	Unfixed_IgG1.fcs	*73.6*
	Unfixed_Secondary Only.fcs	*75.1*

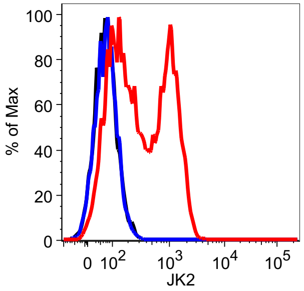

	Sample	Mean:PE-A
	Unfixed_c-MET.fcs	543
	Unfixed_IgG1.fcs	*73.9*
	Unfixed_Secondary Only.fcs	*73.1*

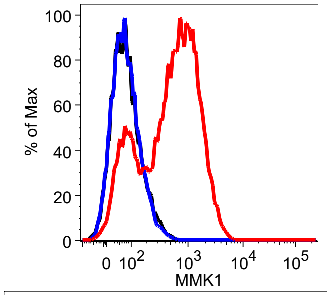

	Sample	Mean:PE-A
	Unfixed_c-MET.fcs	791
	Unfixed_IgG1.fcs	*83.6*
	Unfixed_Secondary only.fcs	*85.6*

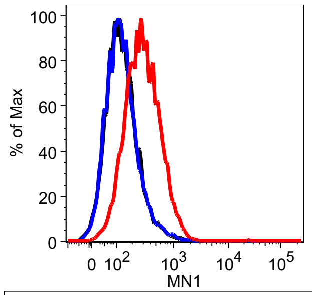

	Sample	Mean:PE-A
	Unfixed_c-MET.fcs	404
	Unfixed_IgG1.fcs	*153*
	Unfixed_Secondary only.fcs	*154*

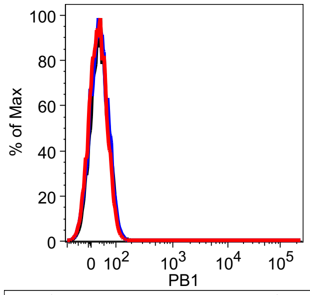

	Sample	Mean:PE-A
	Unfixed_c-MET.fcs	24.5
	Unfixed_IgG1.fcs	*27.9*
	Unfixed_Secondary Only.fcs	*29*

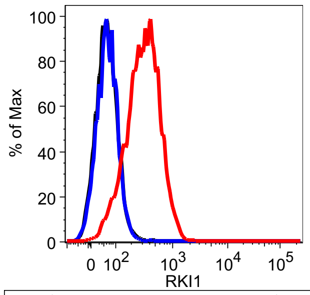

	Sample	Mean:PE-A
	Unfixed_c-MET.fcs	375
	Unfixed_IgG1.fcs	*63.8*
	Unfixed_Secondary only.fcs	*63.1*

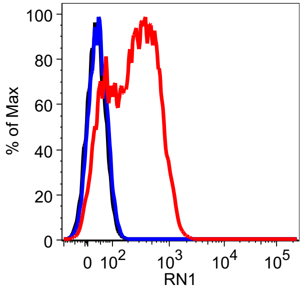

	SampleName	Mean:PE-A
	Unfixed_c-MET.fcs	327
	Unfixed_IgG1.fcs	*36.7*
	Unfixed_Secondary only.fcs	*30.7*

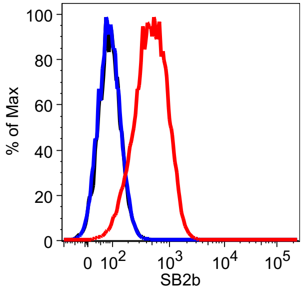

	Sample	Mean:PE-A
	Unfixed_c-MET.fcs	573
	Unfixed_IgG1.fcs	*89.5*
	Unfixed_Secondary only.fcs	*90.5*

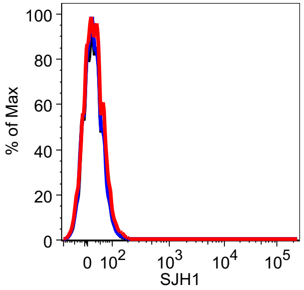

	Sample	Mean:PE-A
	Unfixed_c-MET.fcs	21.6
	Unfixed_IgG1.fcs	*18.3*
	Unfixed_Secondary Only.fcs	*18.9*

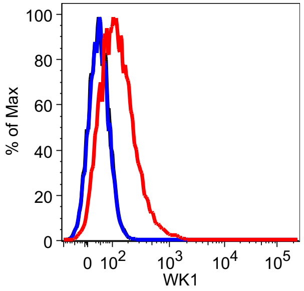

	Sample	Mean:PE-A
	Unfixed_c-MET.fcs	159
	Unfixed_IgG1.fcs	*42.8*
	Unfixed_Secondary only.fcs	*43.1*

SOX2

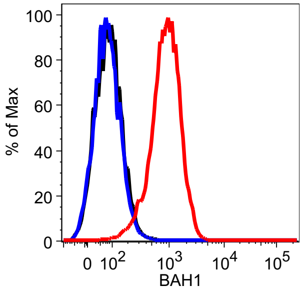

Sample	Mean:PE-A
Fixed_Sox2.fcs	1016
Fixed_IgG1.fcs	*83.5*
Fixed_Secondary.fcs	*89.5*

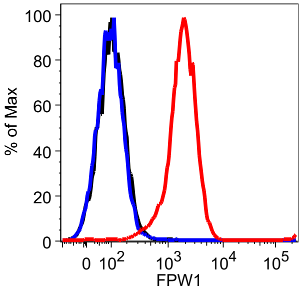

Sample	Mean:PE-A
Fixed_Sox2.fcs	3401
Fixed_IgG1.fcs	*148*
Fixed_Secondary Only.fcs	*216*

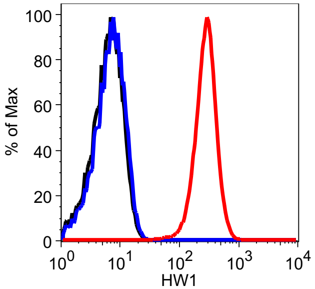

Sample	Mean:PE-A
Fixed Cells_Sox2.fcs	318
Fixed Cells_IgG1.fcs	*7.52*
Fixed Cells_Secondary Only.fcs	*7.07*

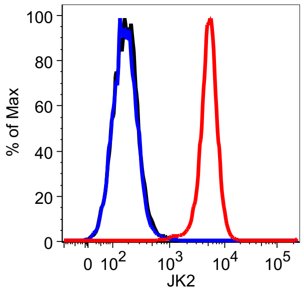

Sample	Mean:PE-A
Fixed_Sox2.fcs	6033
Fixed_IgG1.fcs	*251*
Fixed_Secondary Only.fcs	*197*

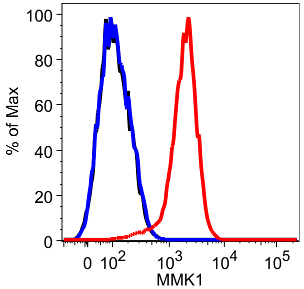

Sample	Mean:PE-A
Fixed_SOX2.fcs	2133
Fixed_IgG1.fcs	*131*
Fixed_Secondary.fcs	*128*

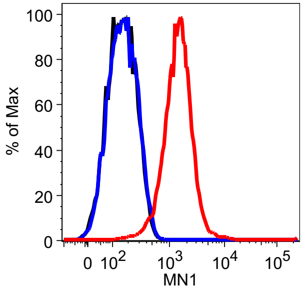

Sample	Mean:PE-A
Fixed_SOX2.fcs	2053
Fixed_IgG1.fcs	*183*
Fixed_Secondary.fcs	*172*

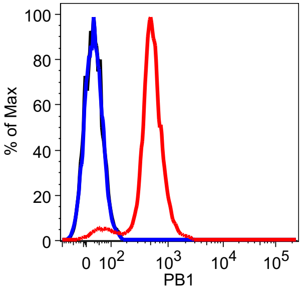

Sample	Mean:PE-A
Fixed_Sox2.fcs	555
Fixed_Secondary Only.fcs	*25.5*
Fixed_IgG1.fcs	*25.7*

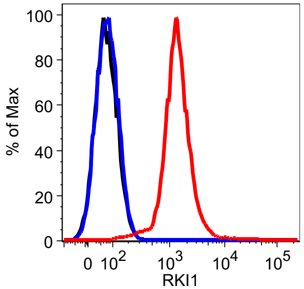

Sample	Mean:PE-A
Fixed_SOX2.fcs	1898
Fixed_IgG1.fcs	*78.2*
Fixed_Secondary.fcs	*75.4*

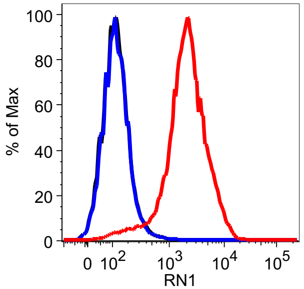

Sample	Mean:PE-A
Fixed_SOX2.fcs	2845
Fixed_IgG1.fcs	*135*
Fixed_Secondary.fcs	*132*

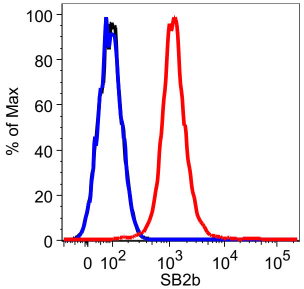

Sample	Mean:PE-A
Fixed_SOX2.fcs	1897
Fixed_IgG1.fcs	*93.6*
Fixed_Secondary.fcs	*93.6*

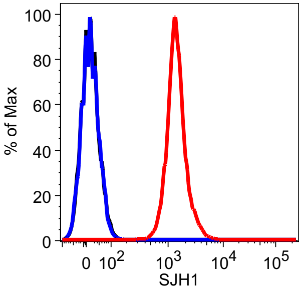

Sample	Mean:PE-A
Fixed_SOX2.fcs	1645
Fixed_IgG1.fcs	*12.7*
Fixed_Secondary Only.fcs	*13.1*

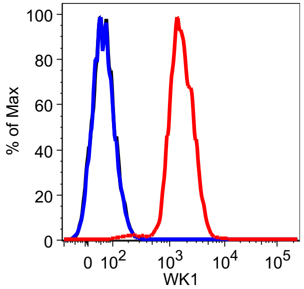

Sample	Mean:PE-A
Fixed_SOX2.fcs	2001
Fixed_IgG1.fcs	*67.4*
Fixed_Secondary.fcs	*64.1*

Nestin

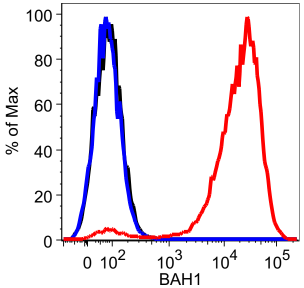

	Sample	Mean:PE-A
Red	Fixed_Nestin.fcs	26601
Blue	*Fixed_IgG1.fcs*	*83.5*
Black	*Fixed_Secondary.fcs*	*89.5*

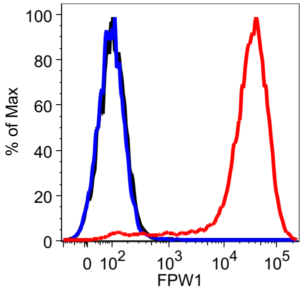

	Sample	Mean:PE-A
Red	Fixed_Nestin.fcs	39824
Blue	*Fixed_IgG1.fcs*	*148*
Black	*Fixed_Secondary Only.fcs*	*216*

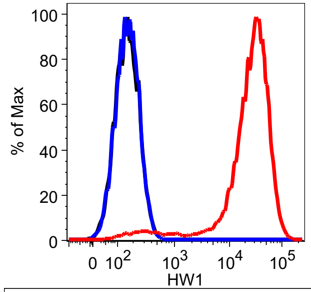

	Sample	Mean:PE-A
Red	Fixed_Nestin.fcs	32632
Blue	*Fixed_IgG1.fcs*	*166*
Black	*Fixed_Secondary Only.fcs*	*165*

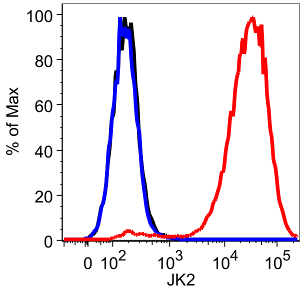

	Sample	Mean:PE-A
Red	Fixed_Nestin.fcs	38025
Blue	*Fixed_IgG1.fcs*	*251*
Black	*Fixed_Secondary Only.fcs*	*197*

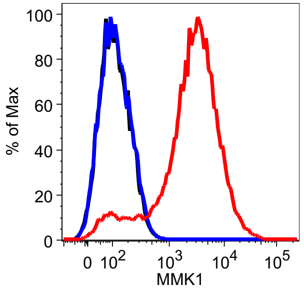

	Sample	Mean:PE-A
Red	Fixed_Nestin.fcs	4289
Blue	*Fixed_IgG1.fcs*	*131*
Black	*Fixed_Secondary.fcs*	*128*

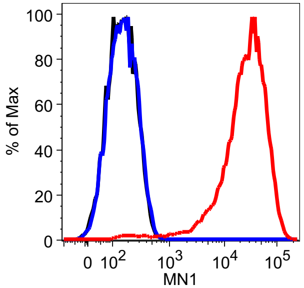

	Sample	Mean:PE-A
Red	Fixed_Nestin.fcs	34970
Blue	*Fixed_IgG1.fcs*	*183*
Black	*Fixed_Secondary.fcs*	*172*

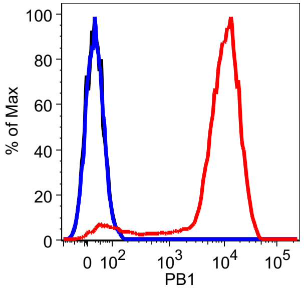

	Sample	Mean:PE-A
Red	Fixed_Nestin.fcs	11490
Blue	*Fixed_Secondary Only.fcs*	*25.5*
Black	*Fixed_IgG1.fcs*	*25.7*

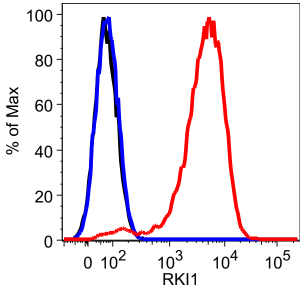

	Sample	Mean:PE-A
Red	Fixed_Nestin.fcs	5619
Blue	*Fixed_IgG1.fcs*	*78.2*
Black	*Fixed_Secondary.fcs*	*75.4*

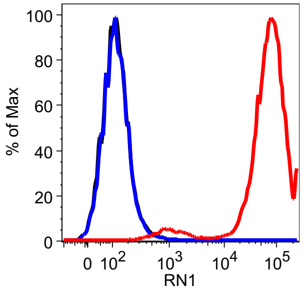

	Sample	Mean:PE-A
Red	Fixed_Nestin.fcs	86592
Blue	*Fixed_IgG1.fcs*	*135*
Black	*Fixed_Secondary.fcs*	*132*

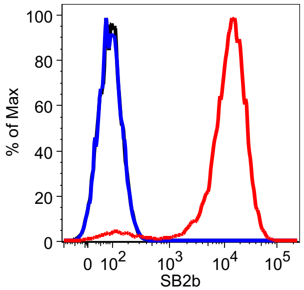

	Sample	Mean:PE-A
Red	Fixed_Nestin.fcs	15366
Blue	*Fixed_IgG1.fcs*	*93.6*
Black	*Fixed_Secondary.fcs*	*93.6*

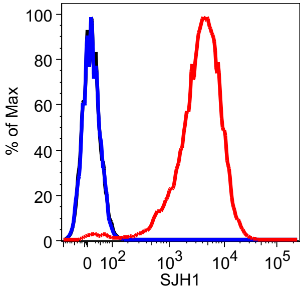

	Sample	Mean:PE-A
Red	Fixed_Nestin.fcs	5033
Blue	*Fixed_IgG1.fcs*	*12.7*
Black	*Fixed_Secondary Only.fcs*	*13.1*

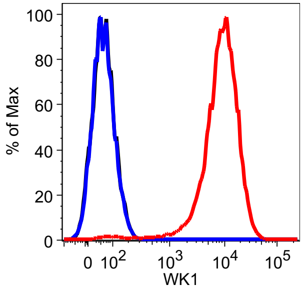

	Sample	Mean:PE-A
Red	Fixed_Nestin.fcs	11018
Blue	*Fixed_IgG1.fcs*	*67.4*
Black	*Fixed_Secondary.fcs*	*64.1*

GFAP

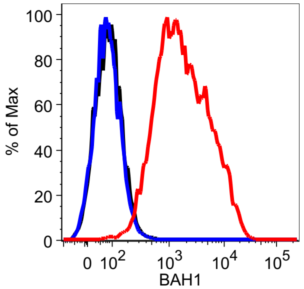

Sample	Mean:PE-A
Fixed_GFAP.fcs	3205
Fixed_IgG1.fcs	*83.5*
Fixed_Secondary.fcs	*89.5*

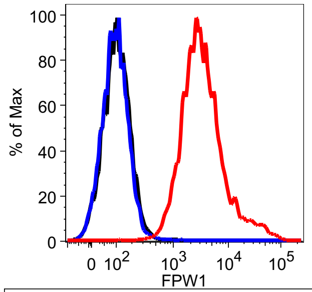

Sample	Mean:PE-A
Fixed_GFAP.fcs	6208
Fixed_IgG1.fcs	*148*
Fixed_Secondary Only.fcs	*216*

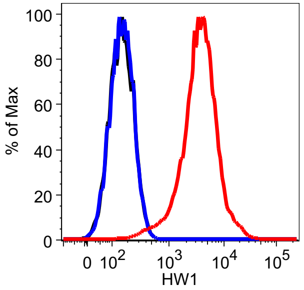

Sample	Mean:PE-A
Fixed_GFAP.fcs	4747
Fixed_IgG1.fcs	*166*
Fixed_Secondary Only.fcs	*165*

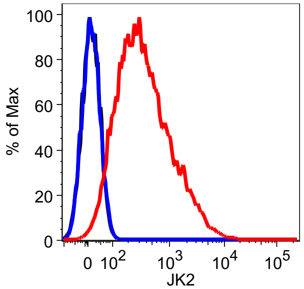

Sample	Mean:PE-A
Fixed_GFAP.fcs	715
Fixed_IgG1.fcs	*9.17*
Fixed_Secondary Only.fcs	*8.76*

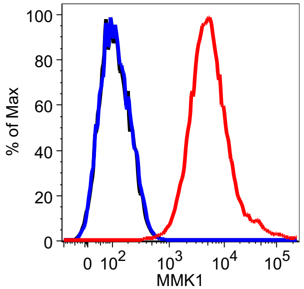

Sample	Mean:PE-A
Fixed_GFAP.fcs	9746
Fixed_IgG1.fcs	*131*
Fixed_Secondary.fcs	*128*

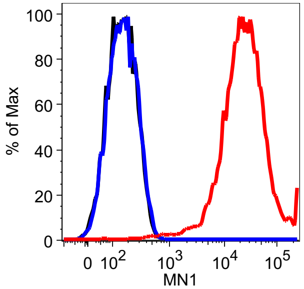

Sample	Mean:PE-A
Fixed_GFAP.fcs	36510
Fixed_IgG1.fcs	*183*
Fixed_Secondary.fcs	*172*

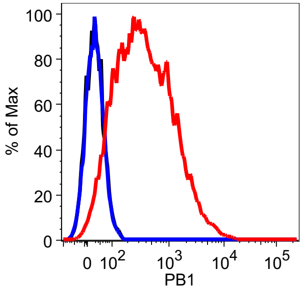

Sample	Mean:PE-A
Fixed_GFAP.fcs	733
Fixed_Secondary Only.fcs	*25.5*
Fixed_IgG1.fcs	*25.7*

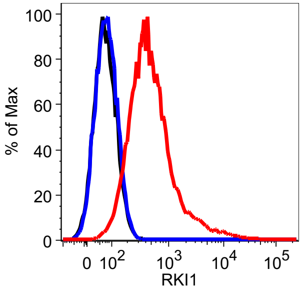

Sample	Mean:PE-A
Fixed_GFAP.fcs	1085
Fixed_IgG1.fcs	*78.2*
Fixed_Secondary.fcs	*75.4*

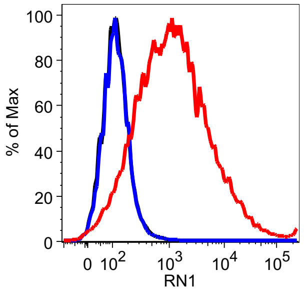

Sample	Mean:PE-A
Fixed_GFAP.fcs	5004
Fixed_IgG1.fcs	*135*
Fixed_Secondary.fcs	*132*

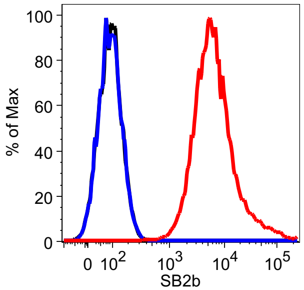

Sample	Mean:PE-A
Fixed_GFAP.fcs	11626
Fixed_IgG1.fcs	*93.6*
Fixed_Secondary.fcs	*93.6*

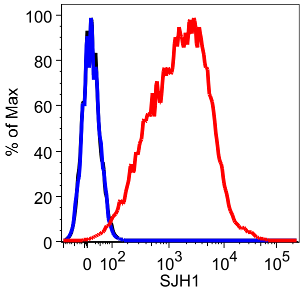

Sample	Mean:PE-A
Fixed_GFAP.fcs	3022
Fixed_IgG1.fcs	*12.7*
Fixed_Secondary Only.fcs	*13.1*

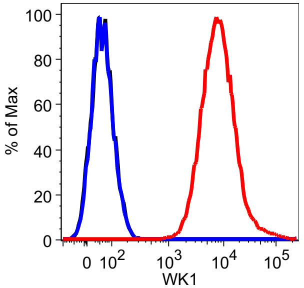

Sample	Mean:PE-A
Fixed_GFAP.fcs	11625
Fixed_IgG1.fcs	*67.4*
Fixed_Secondary.fcs	*64.1*

Beta-III Tubulin

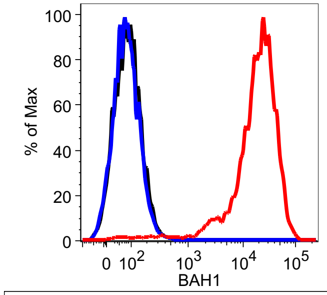

Sample	Mean:PE-A
Fixed_Beta-III Tubulin.fcs	24511
Fixed_IgG1.fcs	*83.5*
Fixed_Secondary.fcs	*89.5*

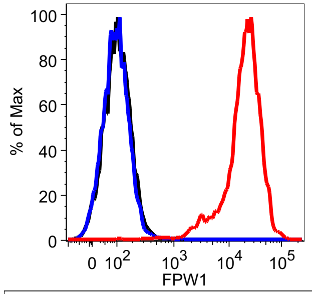

Sample	Mean:PE-A
Fixed_Beta-III Tubulin.fcs	24810
Fixed_IgG1.fcs	*148*
Fixed_Secondary Only.fcs	*216*

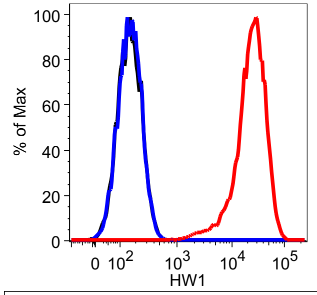

Sample	Mean:PE-A
Fixed_Beta-III Tubulin.fcs	28204
Fixed_IgG1.fcs	*166*
Fixed_Secondary Only.fcs	*165*

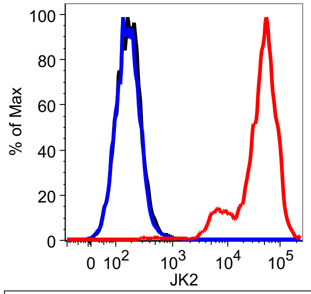

Sample	Mean:PE-A
Fixed_Beta-III Tubulin.fcs	52810
Fixed_IgG1.fcs	*251*
Fixed_Secondary Only.fcs	*197*

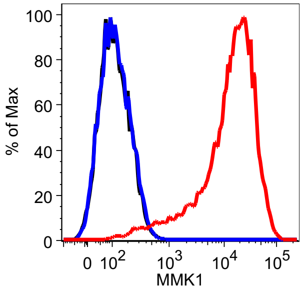

Sample	Mean:PE-A
Fixed_Beta-III Tubulin.fcs	20192
Fixed_IgG1.fcs	*131*
Fixed_Secondary.fcs	*128*

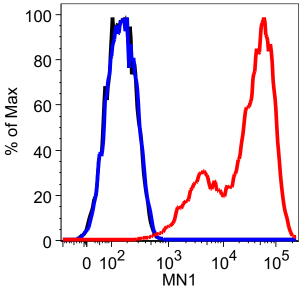

Sample	Mean:PE-A
Fixed_Beta-III Tubulin.fcs	44200
Fixed_IgG1.fcs	*183*
Fixed_Secondary.fcs	*172*

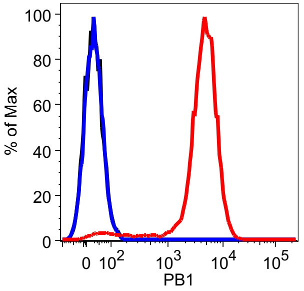

Sample	Mean:PE-A
Fixed_beta-III Tubulin.fcs	4993
Fixed_Secondary Only.fcs	*25.5*
Fixed_IgG1.fcs	*25.7*

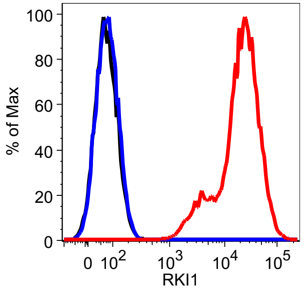

Sample	Mean:PE-A
Fixed_Beta-III Tubulin.fcs	26637
Fixed_IgG1.fcs	*78.2*
Fixed_Secondary.fcs	*75.4*

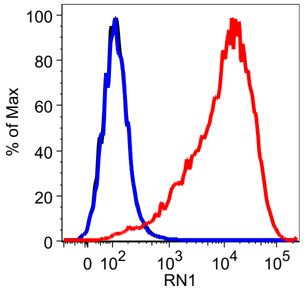

Sample	Mean:PE-A
Fixed_Beta-III Tubulin.fcs	16848
Fixed_IgG1.fcs	*135*
Fixed_Secondary.fcs	*132*

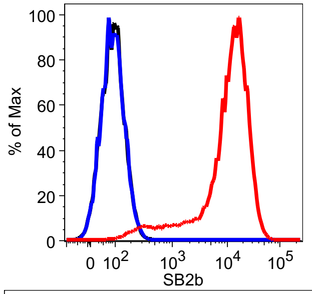

Sample	Mean:PE-A
Fixed_Beta-III Tubulin.fcs	14266
Fixed_IgG1.fcs	*93.6*
Fixed_Secondary.fcs	*93.6*

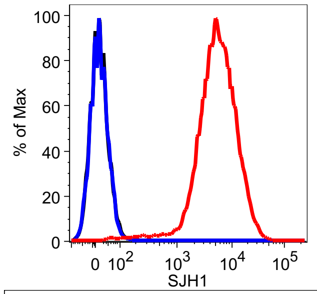

Sample	Mean:PE-A
Fixed_Beta-III Tubulin.fcs	7388
Fixed_IgG1.fcs	*12.7*
Fixed_Secondary Only.fcs	*13.1*

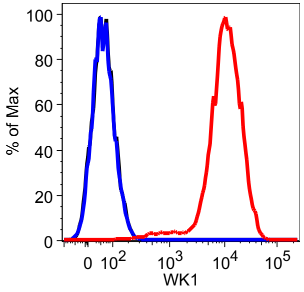

Sample	Mean:PE-A
Fixed_Beta-III Tubulin.fcs	13057
Fixed_IgG1.fcs	*67.4*
Fixed_Secondary.fcs	*64.1*

MBP

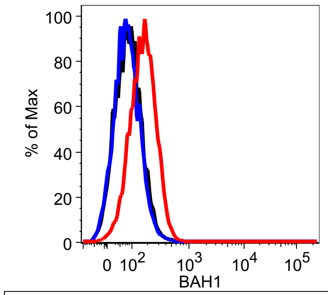

Sample	Mean:PE-A
Fixed_MBP.fcs	179
Fixed_IgG1.fcs	83.5
Fixed_Secondary.fcs	89.5

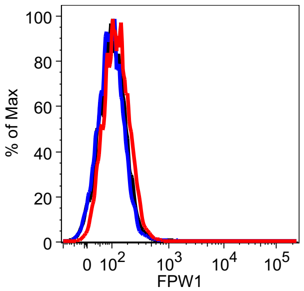

Sample	Mean:PE-A
Fixed_MBP.fcs	143
Fixed_IgG1.fcs	148
Fixed_Secondary Only.fcs	216

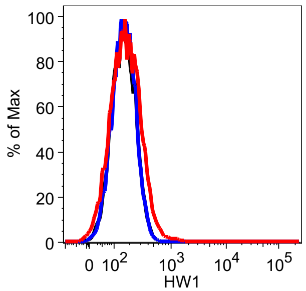

Sample	Mean:PE-A
Fixed_MBP.fcs	196
Fixed_IgG1.fcs	166
Fixed_Secondary Only.fcs	165

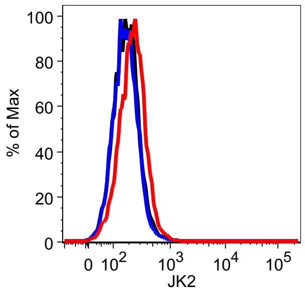

Sample	Mean:PE-A
Fixed_MBP.fcs	331
Fixed_IgG1.fcs	251
Fixed_Secondary Only.fcs	197

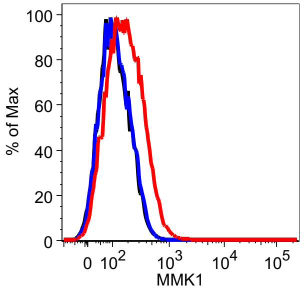

Sample	Mean:PE-A
Fixed_MBP.fcs	255
Fixed_IgG1.fcs	131
Fixed_Secondary.fcs	128

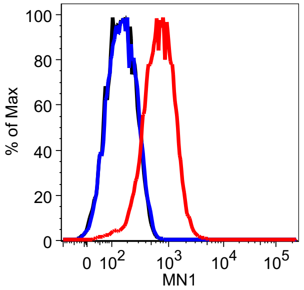

Sample	Mean:PE-A
Fixed_MBP.fcs	1038
Fixed_IgG1.fcs	183
Fixed_Secondary.fcs	172

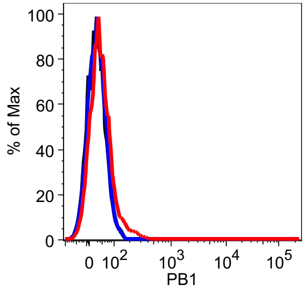

Sample	Mean:PE-A
Fixed_MBP.fcs	43.8
Fixed_Secondary Only.fcs	25.5
Fixed_IgG1.fcs	25.7

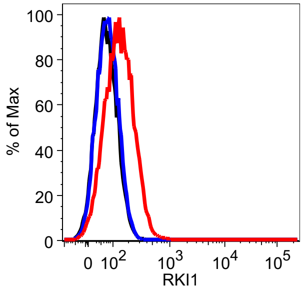

Sample	Mean:PE-A
Fixed_MBP.fcs	192
Fixed_IgG1.fcs	78.2
Fixed_Secondary.fcs	75.4

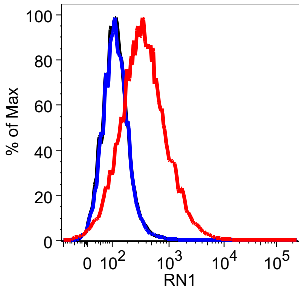

Sample	Mean:PE-A
Fixed_MBP.fcs	676
Fixed_IgG1.fcs	135
Fixed_Secondary.fcs	132

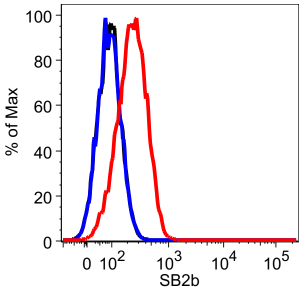

Sample	Mean:PE-A
Fixed_MBP.fcs	275
Fixed_IgG1.fcs	93.6
Fixed_Secondary.fcs	93.6

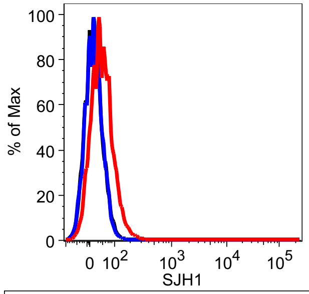

Sample	Mean:PE-A
Fixed_MBP.fcs	80.1
Fixed_IgG1.fcs	12.7
Fixed_Secondary Only.fcs	13.1

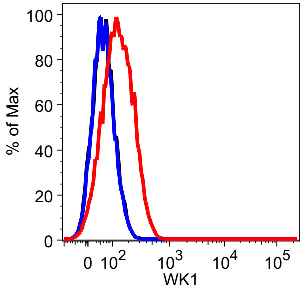

Sample	Mean:PE-A
Fixed_MBP.fcs	202
Fixed_IgG1.fcs	67.4
Fixed_Secondary.fcs	64.1
